# Knowledge translation strategies for dissemination with a focus on healthcare recipients: an overview of systematic reviews

**DOI:** 10.1186/s13012-020-0974-3

**Published:** 2020-03-04

**Authors:** Evelina Chapman, Michelle M. Haby, Tereza Setsuko Toma, Maritsa Carla de Bortoli, Eduardo Illanes, Maria Jose Oliveros, Jorge O. Maia Barreto

**Affiliations:** 1 Fundação Oswaldo Cruz, Fiocruz, Brasília, Brazil; 2 Departamento de Ciencias Químico Biológicas, Universidad de Sonora, Hermosillo, Sonora Mexico; 3 Centre for Health Policy, Melbourne School of Population and Global Health, The University of Melbourne, Melbourne, VIC Australia; 4 Instituto de Saúde, Secretaria de Estado da Saúde de São Paulo, São Paulo, Brazil; 5 School of Psychology Universidad Mayor, Santiago, Chile; 6 Departamento de Medicina Interna, Facultad de Medicina, Universidad de La Frontera, Temuco, Chile

**Keywords:** Knowledge translation, Research uptake, Consumers, Patients, Caregivers

## Abstract

**Background:**

While there is an ample literature on the evaluation of knowledge translation interventions aimed at healthcare providers, managers, and policy-makers, there has been less focus on patients and their informal caregivers. Further, no overview of the literature on dissemination strategies aimed at healthcare users and their caregivers has been conducted. The overview has two specific research questions: (1) to determine the most effective strategies that have been used to disseminate knowledge to healthcare recipients, and (2) to determine the barriers (and facilitators) to dissemination of knowledge to this group.

**Methods:**

This overview used systematic review methods and was conducted according to a pre-defined protocol. A comprehensive search of ten databases and five websites was conducted. Both published and unpublished reviews in English, Spanish, or Portuguese were included. A methodological quality assessment was conducted; low-quality reviews were excluded. A narrative synthesis was undertaken, informed by a matrix of strategy by outcome measure. The Health System Evidence taxonomy for “consumer targeted strategies” was used to separate strategies into one of six categories.

**Results:**

We identified 44 systematic reviews that describe the effective strategies to disseminate health knowledge to the public, patients, and caregivers. Some of these reviews also describe the most important barriers to the uptake of these effective strategies. When analyzing those strategies with the greatest potential to achieve behavioral changes, the majority of strategies with sufficient evidence of effectiveness were combined, frequent, and/or intense over time. Further, strategies focused on the patient, with tailored interventions, and those that seek to acquire skills and competencies were more effective in achieving these changes. In relation to barriers and facilitators, while the lack of health literacy or e-literacy could increase inequities, the benefits of social media were also emphasized, for example by widening access to health information for ethnic minorities and lower socioeconomic groups.

**Conclusions:**

Those interventions that have been shown to be effective in improving knowledge uptake or health behaviors should be implemented in practice, programs, and policies—if not already implemented. When implementing strategies, decision-makers should consider the barriers and facilitators identified by this overview to ensure maximum effectiveness.

**Protocol registration:**

PROSPERO: CRD42018093245.

Contributions to the literature

- Much evidence has been developed to ensure that the results of research are used by health policy-makers and practitioners. However, the challenges of research use for patients and the public are greater and there is less research in this area.
- This review is the first synthesis of systematic review evidence that can help ensure that research results are used by patients and the public and that is not limited to specific diseases.
- The use of Information and Communication Technologies is the new great challenge to increase access and to achieve greater equity in health, especially in low-middle income countries.

## Background

Knowledge translation (KT) is “the synthesis, exchange, and application of knowledge by relevant stakeholders to accelerate the benefits of global and local innovation in strengthening health systems and improving people’s health” [1]. The process of KT ensures that evidence from research is used by relevant stakeholders, including healthcare providers, managers, policy-makers, informal caregivers, patients, and the public in the improvement of health [2]. While there is an ample literature on the evaluation of interventions aimed at healthcare providers, managers, and policy-makers, there has been less focus on patients and their informal caregivers.

“Patient-mediated” KT interventions are those strategies that involve patients in their own healthcare and have the aim to improve patient knowledge, relationship with the provider, the appropriateness of health service use, satisfaction with the provision of care experience, adherence to the recommended treatment, and other health behaviors and outcomes [3].

The Canadian Institutes for Health Research (CIHR), a leader in the science and practice of knowledge translation, have recognized four key elements in the process of KT: synthesis, dissemination, exchange, and ethically sound application of knowledge. For this overview, we will be focusing on dissemination as a core strategy in KT. Dissemination involves identifying the appropriate audience and tailoring the message and medium to the audience [4]. Dissemination of health-related information is the active, tailored, and targeted distribution of information or interventions via determined channels using planned strategies to a specific public health or clinical practice audience, and has been characterized as a necessary but not sufficient antecedent of knowledge adoption and implementation [5]. According to CIHR, dissemination can include elements such as summaries for/briefings to stakeholders, educational sessions with patients, practitioners and/or policy makers, engaging knowledge users in developing and executing dissemination/implementation plans, tools creation, and media engagement. Dissemination can be done through different information and communication technologies (ICT) based or not on the internet, i.e., videos, websites, brochures, decision aids, or art pieces.

There are many models and theories to explain what makes KT for healthcare recipients (and providers) effective [6–9]. These theories have varying objectives, which range from information provision individually or to large audiences (e.g., mass media) to achieving behavior change through education or skills acquisition. When focusing on behavior change, the aim is to increase the capacity to use and apply evidence effectively, thus achieving better health outcomes including quality of life. Desired outcomes of these models include shared decision-making between patients, their families, and providers; patient-provider communication; self-efficacy; adherence; improved access; and cure or survival. Intermediate outcomes could include healthcare users’ improved health knowledge, health behaviors, and physiologic measures; patient satisfaction; and reduced costs [10].

Further, in KT processes addressed to patients and informal caregivers, it is important to consider determinants or barriers at the level of healthcare recipients, i.e., knowledge, language, and cultural differences, skills deficits, attitudes, access to care and motivation to change, among others [7, 10–12]. Also, it is usual practice to combine multicomponent dissemination strategies such as a combination of reach, motivation, or ability goals.

For the purpose of this overview, we have focused on dissemination strategies aimed at healthcare users and their caregivers in order to improve health and wellbeing. We used the taxonomy developed by Lavis et al. to organize the results, which includes six groups of strategies that are explained later [13].

This overview addressed two specific research questions:

1. How effective are the strategies that have been used to disseminate knowledge to healthcare recipients (both for the general public and patients)?
2. What are the barriers (and facilitators) to disseminate knowledge to healthcare recipients (both for the general public and patients)?

## Methods

This overview used systematic review methodology and adheres to the Preferred Reporting Items for Systematic Reviews and Meta-Analysis (PRISMA) statement [14]. A systematic review protocol was written and registered prior to undertaking the searches [15]. Deviations from the protocol are noted.

### Inclusion criteria for studies

Studies were selected based on the following inclusion criteria.

#### Types of studies

Systematic reviews (SRs) that included quantitative studies of any design that provided information on the effectiveness of dissemination strategies. SRs of qualitative studies that describe barriers and facilitators to uptake of research evidence were also included.

#### Types of participants

We included studies that involved healthcare recipients as the main focus, such as the general public, patients, caregivers, or patient groups. We excluded studies where other users, such as practitioners, policy-makers, educators, decision-makers, health care administrators, and community leaders, were the main focus. We also excluded studies where the dissemination strategy was directed to participants with a single health issue, e.g., multimedia interventions to promote HIV testing. This was to ensure a more general approach to strategies for dissemination of knowledge to healthcare recipients.

#### Types of interventions

SRs that evaluated KT dissemination strategies aimed at healthcare recipients or caregivers were included. The dissemination strategies were defined based on the Health System Evidence taxonomy [13] for “consumer targeted strategies” as follow:

- Information or education provision: strategies to enable consumers to know about their treatment and their health.
- Behavior change support: interventions which focus on the adoption or promotion of health and treatment behaviors at an individual level, such as adherence to medicines.
- Skills and competencies development: strategies that focus on the acquisition of skills relevant to self-management.
- (Personal) Support: interventions which provide assistance and encouragement to help patients cope with and manage their health and ongoing medical issues, such as counseling and follow up on treatment efficacy.
- Communication and decision-making facilitation: strategies to involve consumers in decision-making about healthcare.
- System participation: interventions to involve patients and/or caregivers in decision-making processes at a system level.

The dissemination element could be written on paper (i.e., pamphlets, flyers, booklets), verbal (i.e., using telephone), or written or verbal using ICT (i.e., e-health, m-health, websites, multimedia, telemedicine, patient reminder, etc.). The dissemination could be done individually, in groups, or massively.

#### Types of comparisons

There were no restrictions on types of comparisons.

#### Types of outcome measures

We included outcomes related to the effectiveness of dissemination strategies addressed to health-care recipients, caregivers, or the general public, including change in knowledge, understanding, perception, attitudes, adherence to health recommendations, and behavior changes. Other proposed results were health status, access, use of services, social outcomes, user satisfaction, costs, and cost-effectiveness. Additionally, we considered barriers to uptake of research evidence through dissemination strategies at the level of knowledge, competency, health literacy, attitudes, access to care, and motivation to change.

Publications in English, Spanish, or Portuguese were included and there were no restrictions on the year of publication. Both published and gray literature were included.

### Search strategy and sources of systematic reviews

A comprehensive search of ten databases and five websites was conducted. The databases searched for SRs were MEDLINE (Ovid); Embase (Ovid); ERIC (EBSCOHost); CINAHL (EBSCOHost); PsycINFO (Ovid); LILACS (BVSalud); and World Wide Science. The specialized sources of SRs were the Cochrane Library (including Cochrane Reviews, the Database of Abstracts of Reviews of Effects and Health Technology Assessment); Epistemonikos; and Health Systems Evidence.

Manual searches were conducted in Google and Google Scholar; EPPI-Center Systematic Reviews; Rx for Change (https://www.cadth.ca/rx-change); and 3ie–International Initiative for Impact Evaluation. In addition to the above sources that included gray literature, we manually searched the System for Information on Grey Literature in Europe (Open Grey—http://www.opengrey.eu).

Electronic searches were conducted between 21 and 23 May 2018 and supplementary searches (reference lists, contact with authors, and gray literature) were conducted in January 2019. Databases were searched using keywords from keyword areas related to the participants, the intervention, outcomes, and study type—combined using “AND.” Keywords were searched for in the title and abstract fields and using Medical Subject Headings (MeSH) terms where available (search terms and strategies for the electronic searches are in Additional file 1). Results were downloaded into the EndNote reference management program (version X8.2) and duplicates were removed.

### Screening and selection of studies

Titles and abstracts were screened independently according to the selection criteria by pairs of review authors (EC, JB, and MO). The full text of any potentially relevant papers was retrieved for closer examination. The inclusion criteria were then applied against the full text version of the papers independently by two reviewers (EC and MO). Disagreements regarding eligibility of studies were resolved by discussion, and a third reviewer (JB) consulted when necessary. All studies which initially appeared to meet the inclusion criteria but on inspection of the full text paper did not meet the inclusion criteria are listed in a table “Characteristics of excluded studies” together with reasons for their exclusion.

### Data extraction

Information extracted from included SRs were objectives, study designs and number of studies included, date of last search, intervention/strategy, participants, settings, country of studies, and financing source, as well as outcome measures, findings, barriers, research gaps, and theories or frameworks. Data extraction was shared between six reviewers (EC, MB, TT, EI, MH, and MO) and checked by a second reviewer (EC). Disagreements were resolved through discussion and consensus.

After extracting data from the included SRs, reviewers completed a matrix previously designed using the Health Systems Evidence taxonomy [13] for each of the six strategies down the left-hand side with the different outcome measures across the top. While we started with the list of outcome measures specified in the protocol we had to expand the matrix because we found more types of outcome measures than originally proposed. Classification was done by each reviewer (EC, MB, TT, EI, MH, and MO) and checked by a second reviewer (EC).

### Assessment of methodological quality

The methodological quality of included SRs was assessed independently by pairs of reviewers using A MeaSurement Tool to Assess systematic Reviews (AMSTAR) [16]. Disagreements in scoring were resolved by discussion and consensus. For this overview, SRs that achieved AMSTAR scores of 8 to 11 were considered high quality, scores of 4 to 7 medium quality, and scores of 0 to 3 low quality. SRs of low quality were excluded. We did not find any SRs of exclusively qualitative studies to inform barriers and facilitators so could not use the Confidence in the Evidence from Reviews of Qualitative research approach to assess the confidence of findings from qualitative syntheses [17] as proposed in the protocol. Instead, we found SRs with a mix of study designs that included qualitative research so the overall quality of these studies was evaluated with AMSTAR tool.

### Data analysis

Findings from the included publications were synthesized using tables and a narrative summary informed by the matrix of strategy by outcome measure. Meta-analysis was not possible because the included studies were heterogeneous in terms of the populations, strategies/interventions tested, and outcomes measured. Further, few studies informed effectiveness measures. Thus, to inform the main results, we developed effectiveness statements using four categories and standardized language as proposed by Ryan et al. [9]. The decision rules took into account the results, their statistical significance, and the quality and number of studies that support the result. The four categories are (1) sufficient evidence, (2) some evidence, (3) insufficient evidence, and (4) insufficient evidence to determine effectiveness (Additional file 2). Category 4 was used to inform research gaps.

## Results

### Search results

**Fig. 1:**
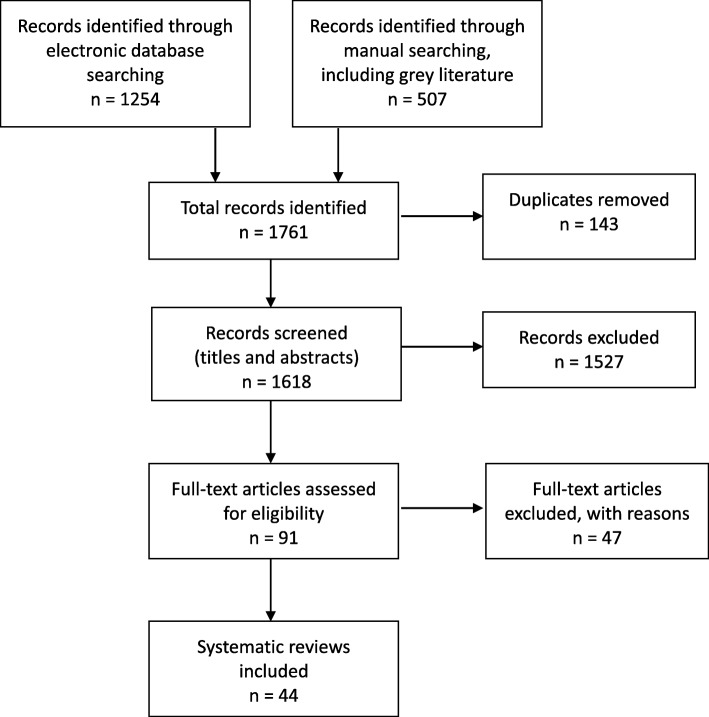
Study selection flow chart

Forty-four SRs met the inclusion criteria for the overview [3, 5, 7, 9–12, 18–54]. The selection process for SRs and the number of papers found at each stage are shown in Fig. 1. The reasons for exclusion of the 47 papers at full text stage are shown in Additional file 3.

### Characteristics of included studies and quality assessment

Details of the characteristics of the included SRs and AMSTAR scores are in Additional file 4 (Tables 1 and 2). Of the 44 SRs, 19 had AMSTAR scores of high quality [7, 9, 10, 12, 20, 21, 24, 31, 35, 36, 38, 43–45, 47–50, 54] and 25 were of medium quality [3, 5, 11, 18, 19, 22, 23, 25–30, 32–34, 37, 39–42, 46, 51–53]. Of the 44 SRs, 24 included both experimental and quasi-experimental designs [3, 5, 7, 9, 11, 12, 20, 21, 25, 27–30, 32–34, 36, 38, 41, 42, 49, 51, 53, 54], ten only included randomized controlled trials [18, 23, 24, 26, 35, 43, 47, 48, 50, 52], and ten included both quantitative and qualitative research [10, 19, 22, 31, 37, 39, 40, 44–46]. Seventeen SRs included only patients or caregivers [7, 9, 10, 20, 24, 31, 32, 34, 35, 37, 39, 40, 43, 44, 49, 50, 53], and the remaining 27 also included providers. Twenty SRs were informed by, or based on, a theory or framework [3, 7, 9, 11, 12, 18, 21, 25, 27, 29, 31, 33, 36, 38, 41, 42, 46, 49, 51, 54].

**Table 1:** Providing information or education—strategies with sufficient or some evidence to support their implementation

Sufficient evidence	Some evidence
Single strategies	
• In relation to alternative formats for presenting risks (in hypothetical scenarios) focused on either diagnostic or screening tests: consumers (and providers) understand formats with natural frequencies better than percentages [21]. • In relation to alternative formats for presenting risk reductions: there is no difference in understanding of relative risk reduction (RRR) compared to absolute risk reduction (ARR). However, RRR is perceived to be larger and more persuasive. RRR is better understood than number needed to treat (NNT) and RRR is perceived to be larger and more persuasive than NNT. ARR is better understood than NNT, with little or no difference in persuasiveness [21]. • When communicating the probability of adverse effects using leaflets on drugs for a particular condition, satisfaction is significantly higher for numbers vs. words (hypothetical scenario) [26].	• Information or education when delivered alone may improve knowledge but there is insufficient evidence for a reduction in adverse effects from drugs [9]. • Patient education and/or information as a single component or as part of a more complex intervention may be effective in improving immunization rates [9]. • Regular viewing of fictional medical television programs habits may improve perceptions of healthcare and healthcare workers [33]. • When communicating the probability of adverse effects using leaflets on drugs for a particular condition numbers vs words (hypothetical scenario) may improve the likelihood of medicines use for very common side effects [26].
Combined strategies	
None identified	• Information or education in combination with other interventions, such as self-management skills training, counseling, or as part of pharmacist delivered packages of care may improve adherence to medications, knowledge and clinical outcomes [9]. • Quality improvement strategies with an educational component targeting patients may decrease the proportion of patients receiving antibiotics, but with mixed results [9]. • Interventions before consultations designed to help patients with their information needs through video, audiotape and computer programs may improve patient satisfaction but there is insufficient evidence regarding their effect on anxiety [35]. • Multimedia or print information as modes of information dissemination and patient education may improve patient preference, knowledge, anxiety, and behavior. (Multimedia could include videotape or DVD, computer, film, slides, html, audiotape only or multiple videos). There was no clear difference in effect between print and multimedia [52].

*ARR* absolute risk reduction, *NNT* number needed to treat, *RRR* relative risk reduction

**Table 2:** Communication and decision-making facilitation—strategies with sufficient or some evidence to support their implementation

Sufficient evidence	Some evidence
Single strategies	
None identified	• Use of email for non-urgent messages between patients and professionals may improve participant satisfaction [24].
Combined strategies	
• Information Technology applications implemented to support Patient-Centered Care improve healthcare process outcomes (i.e., adherence to standards of care, use of resources, patient engagement, etc.), as well as shared decision-making or communication, telehealth communication, and satisfaction among patients and providers [10]. • Use of patient decision aids (written or electronic) improves patient knowledge, accuracy of risk perception, clarity about their personal values and participation in decision-making, and decrease decision conflict [48].	• Coaching plus patient decision aids (versus usual care) may improve knowledge and participation in decision-making. Coaching (versus patient decision aids) may improve values-choice agreement and satisfaction. Coaching plus patient decision aids (versus patient decision aids) showed no differences in knowledge, match between values and choice, participation in decision-making, satisfaction, or decision conflict [47]. • Patient information leaflets before consultation regarding screening or surgery or for medication information may improve patient satisfaction [49]. • One to one risk communication (not necessarily face to face) may be most productive if it includes individual risk estimates in the discussion between the professional and patient. Furthermore, patient decisions about treatments are more likely to change than attendance for screening or modification of risky behavior [28]. • Information Technology applications implemented to support patient-centered care may improve clinical outcomes. In particular, telehealth applications and care management tools may be most effective in improving clinical outcomes. Also tailored health Information Technology interventions aimed at increasing patient engagement during the clinical encounter may improve patient and provider satisfaction [10]. • Consumer health informatics applications (e.g. health risk assessments, decision aids, phones, laptops, CD-ROMs, personal digital assistants/smartphones, short message service (SMS), chat groups or discussion) may effectively engage consumers, enhance traditional clinical interventions, and improve both intermediate and clinical health outcomes [31]. • Delayed prescribing as a strategy to reduce widespread antibiotic resistance may be effective in decreasing antibiotic use, but has mixed effects on clinical outcomes, adverse events and satisfaction [9]. • Education and enhanced follow-up; facilitators working with physicians to encourage preventive services; and pharmaceutical care services – may improve adherence and knowledge [9].

*SMS* short message service

The different strategies tested, and types of communication or dissemination tested in each of the SRs are shown in Additional file 5. When reviewing the included SRs, we found outcome measures that were not included in the protocol (or in the “Methods” section of this review). These included shared decision-making between patients, their families, and providers, patient-provider communication, self-efficacy and/or self-management, awareness, beliefs, clinical results, coverage, use of services, empowerment, less suffering or anxiety, persuasion, safety, social support and influence, quality of life, health status and wellbeing, hospitalizations, length of consultation, participation in health, sustainability, choice, addiction to media, and readability. More details are in Additional file 6.

### Effectiveness statements

The effects of interventions are presented below by strategy according to the adopted taxonomy [13]. The SRs were divided into those testing the specific strategy alone (single) or in combination with other strategies (combined). Many reviews evaluated interventions involving multiple strategies and so contributed evidence to more than one category. More details are in Additional file 5, Table 1. The effectiveness statements are presented in Tables 1, 2, 3, 4, and 5 for those with “sufficient” or “some evidence” (categories 1 and 2 in Additional file 2). Those with “insufficient evidence” (category 3) are in Additional file 7 and those with “insufficient evidence to determine” (category 4) were used to inform the research gaps (Additional file 8).

**Table 3:** Acquiring skills and competencies—strategies with sufficient or some evidence to support their implementation

Sufficient evidence	Some evidence
Single strategies	
None identified	None identified
Combined strategies	
• People self-managing antithrombotics (self-testing and self-adjusting therapy based on a predetermined dose schedule) decreases thromboembolic events and mortality; and there is some evidence that self-management improves clinical outcomes, but with mixed results [9]. • Self-monitoring (self-testing and calling clinic for the appropriate dose adjustment) of antithrombotic is effective in reducing major hemorrhages [9]. • In hypertension, there is also sufficient evidence that home blood pressure monitoring is generally effective to improve clinical markers for hypertension, medicines overuse, and therapeutic inertia [9].	• A home safety toolkit for caregivers of patients with Alzheimer’s improve home safety, risky behavior, caregiver self-efficacy, and caregiver strain [53]. • Strategies that focus on the acquisition of skills and competencies may improve adherence to medicines and clinical outcomes, but results are mixed [9]. • Patient-controlled analgesia may increase analgesic consumption and decrease pain scores, although with mixed results [9]. • Structured patient-controlled analgesia education may improve knowledge, but there is insufficient evidence that it improves postoperative pain control [9]. • Packaged resources or guidelines providing information and/or activation (e.g. information or tools to prompt action for actively managing a condition) are potential sources of self-management support for patients [5]. • Intensive mixed strategy health literacy interventions that promote adherence and facilitate self-management may reduce use of health care services (emergency room visits and hospitalizations) [11]. • Mixed strategy health literacy interventions including individual or group counseling may improve self-management behaviors (e.g. physical activity, foot care, medication adherence, and glucose self-monitoring) [11].

**Table 4:** Behavior change support—strategies with sufficient or some evidence to support their implementation

Sufficient evidence	Some evidence
Single strategies	
None identified	• Video-assisted presentations for patient education may modify behaviors [18]. • Computerized prescribing support interventions can be effectively implemented and may change provider behaviour, but they may be ineffective for improving patient outcomes [9].
Combined strategies	
• “Patient-mediated Knowledge Translation” interventions (defined as strategies that inform, educate and engage patients in their own health care) using print and/or electronic materials before, during or after the consultation improve one or more measures of patient knowledge, decision-making, communication, and behavior [3]. • Internet and mobile phone-based Information technology platforms for delivering behavior change interventions improve health outcomes (e.g., weight loss) and health behaviors across different disease categories [42]. • Interventions using social networking sites (e.g., Facebook, Twitter), specific websites, and email as part of multi-component interventions improve behavior-related outcomes [36]. • Tailored SMS messages combined with other interventions improve targeted behavior changes [30, 50, 54]. • Patient-interactive computer-generated or computer operated interventions—in clinical encounters “in absentia”—as extensions of face-to-face patient care, combined with print materials or telephone positively affect health behavior change [41]. • Text messaging as a tool for behavior change in disease prevention and management improves health behaviors (e.g. smoking cessation by smokers, and blood glucose monitoring and reporting via text message in diabetics) and clinical outcomes (e.g. greater weight loss in obese adults, and greater decrease in hemoglobin A1c levels in adolescents and adult diabetics) [27].	• When attributes of health information are framed negatively (e.g., chance of mortality with cancer) understanding may be better than when the same information is framed positively (e.g., chance of survival with cancer). However, perception may be better when it is positively framed [20]. • When goals of health information are framed as loss messages (e.g., “if you do not undergo screening test for cancer, your survival will be shortened”) there may be a more positive perception of effectiveness for screening messages and may be more persuasive for treatment messages than when framed as gain messages [20]. • Use of patient portals allowing patients to access their personal health information (and may also offer functions and services to enhance medical treatment) may lead to a quicker decrease in office visit rates and slower increase in the number of telephone contacts; increase in number of email messages sent; changes of the medication regimens; and better adherence to treatments [23]. • Online social network health behavior interventions may improve health behaviors [38]. • Reminders, lay health worker interventions, home visits plus vaccination, free vaccination, facilitators working with physicians and financial incentives to physicians may improve immunization rates [9]. • Simplified dosing regimens, reminders, cues and/or organizers, reminder packaging, material incentives, support and education, support and motivation, education and training, or information and counseling interventions may improve medicines adherence, but with mixed results [9]. • Other interventions involving pharmacists directly (such as expanded roles encompassing disease education and medicines management) may improve adherence, numbers of prescribed medicines and clinical outcomes, although results are mixed [9]. • SMS reminders and Multimedia Messaging Service may improve adherence to preventive care [50]. Web-based compared to non-Web-based interventions may improve knowledge or behavior change outcomes in many health conditions. Interventions were delivered using Web-based devices and could include: multimedia, classroom, internet support, help seeking strategies, interactive tools, home-based computer network, computer assisted clinic or Kiosk [51].

*SMS* short message service

**Table 5:** (Personal) support—strategies with sufficient or some evidence to support their implementation

Sufficient evidence	Some evidence
Single strategies	
None identified	None identified
Combined strategies	
• For acute conditions, patient information leaflets improve adherence to treatment in the short-term. For chronic diseases, invasive procedures or screening situations, their impact on adherence varies depending on the context, how they are given and the invasiveness of the intervention [49].	• The provision of counseling of patients and/or physicians by pharmacists may improve adherence, but there is insufficient evidence to support more intensive patient care by pharmacists [9].

#### Providing information or education

Forty-one reviews included this strategy (Additional file 5, Table 1) but only 17 provided evidence that was useful for the development of the effectiveness statements [7, 9, 11, 12, 21, 26, 28, 29, 33–35, 37, 39, 40, 44, 49, 52]. Seven of these 17 reviews were of high quality [7, 9, 12, 21, 35, 44, 49]. The remaining 24 SRs were used to inform the research gaps (Additional file 8). The effectiveness statements are presented in Table 1 and Additional file 7.

#### Communication and decision-making facilitation

Twenty-seven reviews included this strategy (Additional file 5, Table 1) but only 11 provided evidence that was useful for the development of the effectiveness statements [9, 10, 24, 28, 30, 31, 46–50]. Eight of these 11 reviews were of high quality [9, 10, 24, 31, 47–50]. The effectiveness statements are presented in Table 2 and Additional file 7.

#### Acquiring skills and competencies

Twenty-six reviews included this strategy but only five provided evidence that was useful for the development of the effectiveness statements [5, 9, 11, 22, 53]. One of these five reviews was of high quality [9]. See Table 3 and Additional file 7 for the effectiveness statements.

#### Behavior change support

Thirty-nine reviews included this strategy but only 19 provided evidence that was useful for the development of the effectiveness statements [3, 9, 11, 18–20, 23–25, 27, 30, 36, 38, 41–43, 50, 51, 54]. Seven of these reviews were of high quality [9, 20, 21, 24, 36, 38, 43, 50]. See Table 4 and Additional file 7 for the effectiveness statements.

#### Personal support

Thirty reviews included this strategy but only two provided evidence that was useful for the development of the effectiveness statements [9, 49]—both were of high quality. See Table 5 and Additional file 7 for the effectiveness statements.

#### Consumer system participation

Twenty-eight reviews included this strategy but only six provided evidence that was useful for the development of the effectiveness statements [10, 19, 23, 32, 42, 45]—two of these were of high quality [10, 45]. In relation to consumer system participation, no single strategies were identified. For the combined strategies, none had sufficient evidence and only one had some evidence of effectiveness, with the resulting effectiveness statement:

- The use of social media and telemonitoring (ICT platforms) for promoting patient engagement and delivering behavior change interventions may improve health outcomes [42].

The combined strategies with insufficient evidence are listed in Additional file 7.

### Barriers

We did not find any SRs of exclusively qualitative studies. Of the 44 included SRs, ten included qualitative research among other study designs. For the synthesis of barriers (and facilitators) to KT to healthcare participants, 31 SRs contributed information. The barriers identified were grouped following the type of communication used for the intervention or strategy (Additional file 5, Table 2). While this method of grouping barriers was not originally stated in the protocol, we found it to be the most logical way to group them due to the way in which barriers were reported in the included systematic reviews.

None of the SRs identified barriers to verbal communication specifically. However, in relation to patient advisory councils (which may use both verbal and electronic communication), the main barrier described was that the implementation takes a significant amount of time and resources for recruitment, holding meetings, and providing follow up [45].

For written information that does not require the internet, concerns were raised about motivation and awareness [29, 49], health literacy [40, 53], and comprehension and understanding [29, 40]. Other possible barriers that should be considered are the reliability and trustworthiness of the information [29], personal needs [49], and text complexity and design [40].

For information technology interventions in general, barriers raised include health literacy, privacy and information quality concerns, access to technology, and information design [42].

Computer-based strategies, whether internet-based or not, present as barriers difficulties in the management of technologies, mainly for the elderly [19], e-literacy [25, 31, 51], privacy concerns, consumer’s personal feelings, socioeconomic factors [31, 41], and health literacy [31, 44]. Other barriers include reliability and trustworthiness in the information, lack of time and personal impairment [31], motivation and awareness, and information that does not meet personal needs [49], text complexity and lack of access to information [44].

Strategies that use multimedia not based on the internet bring difficulties like motivation, awareness, information that does not respond to personal need or without sufficient detail [49], problems of communication [35], and health literacy [18, 52] for their implementation.

Internet-based multimedia strategies face barriers such as e-literacy [12, 25, 51], health literacy [11, 37, 44], motivation, awareness [12, 29], concerns about reliability and trustworthiness [29, 37], complexity of the text [37, 44], consumer’s personal feelings, information overload, and information that does not match personal needs [37]. Other barriers were lack of internet access and personal skills [12, 24, 44, 51], and comprehension, understanding, and self-management when self-management interventions packaged with guidelines are used [5].

Most of the studies did not discuss issues such as ethnicity, income level, or homelessness, which are important when considering the use of an internet-based technology to deliver an outpatient intervention. The long-term effects on individual persistence with chosen therapies and cost-effectiveness of the use of internet-based therapies and hardware and software development require continued evaluation [51]. Recent SRs have mentioned inequities such as lack of access to technology [42]. However, one review noted that a benefit of social media is that it can widen access to those who may not easily access health information via traditional methods, such as younger people, ethnic minorities, and lower socioeconomic groups [39].

For social media interventions, barriers on the individual level include health literacy [7, 42] and the risk of a deterioration in the relationship between health professionals and patients [7, 39], including the inability to meet the patients’ emotional and information needs [46]. Other concerns include how the information is presented [7, 21], privacy, information quality, lack of internet access, trustworthiness in the information, information overload, and stigma about certain conditions [39]. Another highlighted barrier was the fact that the social content is used more than the educational content, i.e., participants use the social media to interact with other users more than as a means for self-education [38].

m-health (with mobile phone) strategies raise as the main barriers to its implementation issues such as e-literacy and lack of internet access [10, 27], health literacy [34, 44], socioeconomic factors [27, 50], privacy concerns, lack of personal skills [10], text complexity [44], and the time-consuming nature of the technology [54]. Another potential limitation of m-health could be that the delivery of interventions can be interrupted if the mobile phone is stolen or lost. However, the same limitations exist with many other forms of communication (e.g., postal mail may be delivered to the wrong address, email boxes may be too full to receive messages) [27].

Barriers to implementation of telemedicine are also related to e-literacy, privacy concerns, lack of internet access, and personal skills [10].

For the implementation of patient decision aids, which can include pamphlets, videos, or web-based tools, barriers detected include decision aids that do not meet the needs of the population, clinicians unwilling to use them, and clinicians and healthcare consumers without skills for shared decision-making [48].

## Discussion

For this overview, we identified 44 SRs that describe the effective strategies to disseminate health knowledge to the public, patients, and caregivers. Some of these SRs also describe the most important barriers to the uptake of these effective strategies. The reviews that tested more general strategies were selected instead of those directed to a particular condition or setting. To our knowledge, this is the first overview of SRs addressing this objective.

While we reported the strategies and results according to the taxonomy adapted from the Health System Evidence database [13], we found that many strategies overlapped for both the type of intervention and the outcome measures. For example, interventions providing information or education could report outcomes related to behavior change or self-efficacy, and the primary intention could have been to increase knowledge. Situations like these were frequent and could be due to the use of combined strategies or to characteristics of the intervention itself, its intensity, frequency, or duration. The strategies reported in the included SRs could be directed to individuals or groups, in print or verbally, face to face, or remotely. In addition, interventions could range from single (e.g., a written information leaflet) to combined strategies. We considered a strategy to be combined when it used two or more verbal, print, or remote health information strategies (e.g., video, computer, and slide show presentations [11]), or different electronic communication types (based or not on the internet), such as telemedicine, ICT applications or ICT platforms [10, 42], or social networking like Facebook or Twitter [39, 46].

We found few SRs with a meta-analysis that could inform the magnitude of effects. Thus, an overall meta-analysis for each of the strategies could not be conducted, which is why we chose to adopt the approach proposed by Ryan et al. [9] and have presented the findings as evidence statements.

A key objective of the included interventions was to inform, improve knowledge, or to change health behaviors. To achieve behavioral changes, different strategies were used, such as training, coaching, or text messages. Factors that affected the effectiveness of the intervention included its frequency, intensity, and follow-up time. These factors are important to consider when implementing the chosen intervention strategies, including the applicability of the intervention in different modes of implementation and contexts.

When analyzing those strategies with the greatest potential to achieve behavioral changes, the majority of strategies with sufficient evidence of effectiveness were combined, frequent, and/or intense over time. Further, strategies focused on the patient, with tailored interventions, and those that seek to acquire skills and competencies were more effective in achieving these changes. Many of these strategies used toolkits or different platforms, based or not on the internet. Examples of strategies based on the internet include social networks, specific portals, tailored text messaging, or email [23, 27, 30, 36, 42, 43, 50, 54]. Examples of strategies that are not always based on the internet are the use of videos, telephone calls, telemedicine, and telemonitoring [9, 10, 41, 48, 49, 53].

Other examples of effective tailored interventions, such as those designed to improve communication or participation in decision-making between patients and healthcare providers, were the use of patient decision aids and patient information leaflets, provided electronically or not [10, 48, 49]. Interestingly, when coaching was added to patient decision aids, we found some evidence for improvements in knowledge and participation. Also, coaching, when compared to patient decision aids alone, increased values-choice agreement and improved satisfaction with the decision-making process [47]. In relation to satisfaction, we also found some evidence for improvement in patient satisfaction for interventions through multimedia before consultations designed to help patients with their information needs [35].

With regard to caregivers, in particular of patients with Alzheimer’s disease, we found good evidence for the effect of a home safety toolkit for improvements in home safety, risky behavior, and caregiver self-efficacy [53]. For interventions that involved patients and/or caregivers in decision-making processes at a system level, we did not find sufficient evidence to make any statements. Further, few studies included a follow-up period longer than 1 year or reported retention rate, thus it is not known if behavior change results are sustained over time [32, 36, 42, 51].

Our second research question focused on barriers to the dissemination of knowledge to healthcare recipients, which are important to consider when implementing chosen intervention strategies. The barriers most frequently mentioned were related to ICT or to the information itself. For ICT, the main concerns were access to the technologies, including availability of the internet. On a personal level, the lack of skills for managing new technologies, privacy issues, lack of time, and deterioration of the doctor-patient relationship were also mentioned, especially when using social media or websites. As for the information itself, the lack of understanding or comprehension, the volume of information, text complexity and its design, information that did not meet the needs of the patients, and trustworthiness were the key barriers mentioned. While inequities were mentioned and were often related to the lack of health literacy or e-literacy, the benefits of social media were also emphasized, for example by widening access to health information, particularly for ethnic minorities and lower socioeconomic groups.

### Strengths and limitations of the overview

Strengths of our overview were that only reviews of medium or high quality were included, as well as our focus on strategies that translated health information to patients and caregivers through different strategies and types of dissemination. Further, we focused on more general interventions rather than specific interventions, which are already abundant in the scientific literature and could be among the list of SRs that were excluded. While we did not find SRs of qualitative studies to analyze barriers to a better implementation of dissemination interventions, we did find considerable information and analysis of barriers in many of the included SRs. These included good quality studies on health literacy. Further, we were able to identify many research gaps that are detailed in Additional file 8.

Limitations of our overview include limitations in the included SRs, such as the lack of clear description of the interventions, setting or samples, and outcomes in some reviews. Further, not all of the included SRs used theories or frameworks to inform the strategies. Finally, due to the heterogeneity in the interventions and outcomes, a meta-analysis was not possible.

Achieving improvements in knowledge uptake or health behaviors is difficult and the literature of effectiveness for the different strategies in the clinical field has been presented using a range of frameworks, theories, or taxonomies. While work is underway to develop consistent taxonomies for the design and reporting of behavior change and dissemination and implementation interventions, such as the behavior change wheel [55], the theoretical domains framework [56, 57], and other taxonomies [58], these are not consistently applied in the existing literature. Further, few have been developed for patients or their caregivers, and there is more of a focus on implementation rather than dissemination. None of the developed frameworks were suitable for our context (https://dissemination-implementation.org/viewAll_di.aspx). Thus, given that this overview was aimed at healthcare decision-makers, we chose to use the Health Systems Evidence taxonomy of Lavis and colleagues [13]. The advantage of using this taxonomy is that it makes it easier for healthcare decision-makers to find, understand, and use the evidence contained in the overview. Further, while there is debate about how best to measure the effectiveness of complex behavior change interventions [59, 60], these authors acknowledge that further work is needed. Until that work is conducted and consensus achieved, systematic reviews of randomized controlled trials (and other designs), as used in this overview, are the currently accepted best method.

## Conclusions

This overview of systematic reviews has shown that a variety of dissemination strategies aimed at healthcare users and their caregivers can improve health and wellbeing in different ways. However, implementation of our findings will need to consider the particular context in which a strategy is to be implemented. This overview will help decision-makers choose the most effective dissemination strategies and will also inform them as to the factors that they should consider when implementing those strategies.

### Implications for practice and policy

Those interventions that have been shown to be effective in improving knowledge uptake or health behaviors should be implemented in practice, programs, and policies—if not already implemented. The benefits of strategies such as e-health and m-health, including telemedicine, should be considered for knowledge dissemination and to improve health behaviors—especially in populations with lack of access to traditional sources of healthcare, including in remote or rural areas. The application of distance technology may strengthen the continuity of care between patient and clinician by improving access and supporting the coordination of healthcare activities from a single source. When designing KT strategies, not only the effectiveness of the strategy but also the characteristics of the interventions should be taken into account, such as the type of dissemination (electronic or not), frequency, intensity, and follow-up time. It is also important to ensure that the content of the messages is addressed to people with low literacy, low numeracy, and low e-literacy. The knowledge disseminated should be readable, comprehensible, relevant, consistent, unambiguous, and credible for patients. Moreover, patients should be invited to participate in its design. All of these strategies are likely to increase the success of the dissemination.

### Implications for research

Future research should focus on the areas identified as research gaps in Additional file 8. In addition, researchers should ensure that the interventions tested are well described in their papers. Likewise, systematic reviewers should also ensure that they include a clear description of the interventions, settings, samples, and outcomes included in their reviews to facilitate their evaluation and implementation by decision-makers.

## Supplementary information

**Additional file 1.** Search terms and results.

**Additional file 2.** Evidence rating scheme (based on Ryan et al. 2014 [9]).

**Additional file 3 **Excluded studies (*N* = 47).

**Additional file 4.** Characteristics of included studies and AMSTAR quality assessment. Table 1 Characteristics of included systematic reviews. Table 2 Quality of included systematic reviews (AMSTAR).

**Additional file 5.** Strategies and types of communication or dissemination. Table 1 Strategies/Interventions (adapted from Lavis et al. 2015 [13]). Table 2 Types of communication or dissemination. Table 3 Characteristics of interventions (details).

**Additional file 6.** Types of outcome measures.

**Additional file 7.** Strategies categorized as having insufficient evidence.

**Additional file 8.** Research gaps.

## Data Availability

All files supporting the conclusions of this article are included within the article or in Additional files 1, 2, 3, 4, 5, and 6.

## Abbreviations

AMSTAR: A MeaSurement Tool to Assess systematic Reviews
ARR: Absolute risk reduction
CIHR: Canadian Institutes for Health Research
ICT: Information and Communication Technologies
KT: Knowledge translation
MeSH: Medical Subject Headings
NNT: Number needed to treat
PRISMA: Preferred Reporting Items for Systematic Reviews and Meta-Analyses
RRR: Relative risk reduction
SMS: Short message service
SR: Systematic review

## References

[1] Pablos-Mendez A, Shademani R (2006). Knowledge translation in global health. J Contin Educ Heal Prof.

[2] WHO (2012). Knowledge translation framework for Ageing and health.

[3] Gagliardi AR, Legare F, Brouwers MC, Webster F, Badley E, Straus S (2016). Patient-mediated knowledge translation (PKT) interventions for clinical encounters: a systematic review. Implement Sci.

[4] Graham ID, Tetroe JM (2009). Getting evidence into policy and practice: perspective of a health research funder. J Can Acad Child Adolesc Psychiatry.

[5] Vernooij RW, Willson M, Gagliardi AR, members of the Guidelines International Network Implementation Working G (2016). Characterizing patient-oriented tools that could be packaged with guidelines to promote self-management and guideline adoption: a meta-review. Implement Sci.

[6] Albrecht L, Archibald M, Arseneau D, Scott SD (2013). Development of a checklist to assess the quality of reporting of knowledge translation interventions using the workgroup for intervention development and evaluation research (WIDER) recommendations. Implement Sci.

[7] McCormack L, Sheridan S, Lewis M, Boudewyns V, Melvin CL, Kistler C, et al. Communication and dissemination strategies to facilitate the use of health-related evidence. Evidence Report/Technology Assessment No. 213. AHRQ Publication No. 13(14)-E003-EF. Rockville: Agency for Healthcare Research and Quality; 2013. 10.23970/ahrqepcerta213 PMC4781094 24423078

[8] Pantoja T, Opiyo N, Lewin S, Paulsen E, Ciapponi A, Wiysonge CS (2017). Implementation strategies for health systems in low-income countries: an overview of systematic reviews. Cochrane Database Syst Rev.

[9] Ryan R, Santesso N, Lowe D, Hill S, Grimshaw J, Prictor M (2014). Interventions to improve safe and effective medicines use by consumers: an overview of systematic reviews. Cochrane Database Syst Rev.

[10] Finkelstein J, Knight A, Marinopoulos S, Gibbons MC, Berger Z, Aboumatar H, et al. Enabling patient-centered care through health information technology. Evidence Report/Technology Assessment No. 206. AHRQ Publication No. 12-E005-EF. Rockville: Agency for Healthcare Research and Quality. 2012.

[11] Berkman ND, Sheridan SL, Donahue KE, Halpern DJ, Viera A, Crotty K, et al. Health literacy interventions and outcomes: an updated systematic review. Evidence Report/Technology Assesment No. 199. AHRQ Publication Number 11-E006. Rockville: Agency for Healthcare Research and Quality. 2011.

[12] Car J, Lang B, Colledge A, Ung C, Majeed A. Interventions for enhancing consumers’ online health literacy. Cochrane Database Syst Rev. 2011;(6). Art. No.: CD007092. 10.1002/14651858.CD007092.pub2 PMC6464831 21678364

[13] Lavis JN, Wilson MG, Moat KA, Hammill AC, Boyko JA, Grimshaw JM (2015). Developing and refining the methods for a ‘one-stop shop’ for research evidence about health systems. Health Res Policy Syst.

[14] Moher D, Liberati A, Tetzlaff J, Altman DG, Group P (2009). Preferred reporting items for systematic reviews and meta-analyses: the PRISMA statement. PLoS Med.

[15] Chapman E, Barreto JOM. Knowledge translation overview: strategies for dissemination with a focus on recipient health care. PROSPERO 2018 CRD42018093245. Available from: http://www.crd.york.ac.uk/PROSPERO/display_record.php?ID=CRD42018093245.

[16] Shea BJ, Grimshaw JM, Wells GA, Boers M, Andersson N, Hamel C (2007). Development of AMSTAR: a measurement tool to assess the methodological quality of systematic reviews. BMC Med Res Methodol.

[17] Lewin S, Glenton C, Munthe-Kaas H, Carlsen B, Colvin CJ, Gulmezoglu M (2015). Using qualitative evidence in decision making for health and social interventions: an approach to assess confidence in findings from qualitative evidence syntheses (GRADE-CERQual). PLoS Med.

[18] Abu Abed M, Himmel W, Vormfelde S, Koschack J (2014). Video-assisted patient education to modify behavior: a systematic review. Patient Educ Couns.

[19] Akesson KM, Saveman BI, Nilsson G (2007). Health care consumers’ experiences of information communication technology--a summary of literature. Int J Med Inform.

[20] Akl EA, Oxman AD, Herrin J, Vist GE, Terrenato I, Sperati F, et al. Framing of health information messages. Cochrane Database Syst Rev. 2011;(12). Art. No.: CD006777. 10.1002/14651858.CD006777.pub2 PMC12926860 22161408

[21] Akl EA, Oxman AD, Herrin J, Vist GE, Terrenato I, Sperati F (2011). Using alternative statistical formats for presenting risks and risk reductions. Cochrane Database Syst Rev.

[22] Ammentorp J, Uhrenfeldt L, Angel F, Ehrensvard M, Carlsen EB, Kofoed PE (2013). Can life coaching improve health outcomes?—a systematic review of intervention studies. BMC Health Serv Res.

[23] Ammenwerth E, Schnell-Inderst P, Hoerbst A (2012). The impact of electronic patient portals on patient care: a systematic review of controlled trials. J Med Internet Res.

[24] Atherton H, Sawmynaden P, Sheikh A, Majeed A, Car J (2012). Email for clinical communication between patients/caregivers and healthcare professionals. Cochrane Database Syst Rev.

[25] Bekker HL, Winterbottom AE, Butow P, Dillard AJ, Feldman-Stewart D, Fowler FJ (2013). Do personal stories make patient decision aids more effective? A critical review of theory and evidence. BMC Med Inform Decis Mak.

[26] Buchter R, Fechtelpeter D, Knelangen M, Ehrlich M, Waltering A (2014). Words or numbers? Communicating risk of adverse effects in written consumer health information: a systematic review and meta-analysis. BMC Med Inform Decis Mak.

[27] Cole-Lewis H, Kershaw T (2010). Text messaging as a tool for behavior change in disease prevention and management. Epidemiol Rev.

[28] Edwards A, Hood K, Matthews E, Russell D, Russell I, Barker J (2000). The effectiveness of one-to-one risk communication interventions in health care: a systematic review. Med Decis Mak.

[29] Faber M, Bosch M, Wollersheim H, Leatherman S, Grol R (2009). Public reporting in health care: how do consumers use quality-of-care information? A systematic review. Med Care.

[30] Fjeldsoe BS, Marshall AL, Miller YD (2009). Behavior change interventions delivered by mobile telephone short-message service. Am J Prev Med.

[31] Gibbons MC, Wilson RF, Samal L, Lehman CU, Dickersin K, Lehmann HP, et al. Impact of consumer health informatics applications. Evidence Report/Technology Assessment No. 188. AHRQ Publication No. 09(10)-E019. Rockville: Agency for Healthcare Research and Quality. 2009. PMC4780989 20629477

[32] Health Quality Ontario (2013). Electronic tools for health information exchange: an evidence-based analysis. Ont Health Technol Assess Ser.

[33] Hoffman BL, Shensa A, Wessel C, Hoffman R, Primack BA (2017). Exposure to fictional medical television and health: a systematic review. Health Educ Res.

[34] Ketelaar NA, Faber MJ, Flottorp S, Rygh LH, Deane KH, Eccles MP. Public release of performance data in changing the behaviour of healthcare consumers, professionals or organisations. Cochrane Database Syst Rev. 2011;(11). Art. No.: CD004538. 10.1002/14651858.CD004538.pub2 PMC4204393 22071813

[35] Kinnersley P, Edwards A, Hood K, Cadbury N, Ryan R, Prout H, et al. Interventions before consultations for helping patients address their information needs. Cochrane Database Syst Rev. 2007;(3). Art. No.: CD004565. 10.1002/14651858.CD004565.pub2 PMC9036848 17636767

[36] Laranjo L, Arguel A, Neves AL, Gallagher AM, Kaplan R, Mortimer N (2015). The influence of social networking sites on health behavior change: a systematic review and meta-analysis. J Am Med Inform Assoc.

[37] Loudon K, Santesso N, Callaghan M, Thornton J, Harbour J, Graham K (2014). Patient and public attitudes to and awareness of clinical practice guidelines: a systematic review with thematic and narrative syntheses. BMC Health Serv Res.

[38] Maher CA, Lewis LK, Ferrar K, Marshall S, De Bourdeaudhuij I, Vandelanotte C (2014). Are health behavior change interventions that use online social networks effective? A systematic review. J Med Internet Res.

[39] Moorhead SA, Hazlett DE, Harrison L, Carroll JK, Irwin A, Hoving C (2013). A new dimension of health care: systematic review of the uses, benefits, and limitations of social media for health communication. J Med Internet Res.

[40] Pires C, Vigário M, Cavaco A (2015). Readability of medicinal package leaflets: a systematic review. Rev Saude Publica.

[41] Revere D, Dunbar PJ (2001). Review of computer-generated outpatient health behavior interventions: clinical encounters “in absentia”. J Am Med Inform Assoc.

[42] Sawesi S, Rashrash M, Phalakornkule K, Carpenter JS, Jones JF (2016). The impact of information technology on patient engagement and health behavior change: a systematic review of the literature. JMIR Med Inform.

[43] Sawmynaden P, Atherton H, Majeed A, Car J (2012). Email for the provision of information on disease prevention and health promotion. Cochrane Database Syst Rev.

[44] Schipper K, Bakker M, De Wit M, Ket JC, Abma TA (2016). Strategies for disseminating recommendations or guidelines to patients: a systematic review. Implement Sci.

[45] Sharma AE, Knox M, Mleczko VL, Olayiwola JN (2017). The impact of patient advisors on healthcare outcomes: a systematic review. BMC Health Serv Res.

[46] Smailhodzic E, Hooijsma W, Boonstra A, Langley DJ (2016). Social media use in healthcare: a systematic review of effects on patients and on their relationship with healthcare professionals. BMC Health Serv Res.

[47] Stacey D, Kryworuchko J, Bennett C, Murray MA, Mullan S, Legare F (2012). Decision coaching to prepare patients for making health decisions: a systematic review of decision coaching in trials of patient decision AIDS. Med Decis Mak.

[48] Stacey D, Legare F, Lewis K, Barry MJ, Bennett CL, Eden KB (2017). Decision aids for people facing health treatment or screening decisions. Cochrane Database Syst Rev.

[49] Sustersic M, Gauchet A, Foote A, Bosson JL (2017). How best to use and evaluate patient information leaflets given during a consultation: a systematic review of literature reviews. Health Expect.

[50] Vodopivec-Jamsek V, de Jongh T, Gurol-Urganci I, Atun R, Car J (2012). Mobile phone messaging for preventive health care. Cochrane Database Syst Rev.

[51] Wantland DJ, Portillo CJ, Holzemer WL, Slaughter R, McGhee EM (2004). The effectiveness of web-based vs. non-web-based interventions: a meta-analysis of behavioral change outcomes. J Med Internet Res.

[52] Wilson EA, Makoul G, Bojarski EA, Bailey SC, Waite KR, Rapp DN (2012). Comparative analysis of print and multimedia health materials: a review of the literature. Patient Educ Couns.

[53] Yamada J, Shorkey A, Barwick M, Widger K, Stevens BJ (2015). The effectiveness of toolkits as knowledge translation strategies for integrating evidence into clinical care: a systematic review. BMJ Open.

[54] Zhao J, Freeman B, Li M (2016). Can Mobile phone apps influence People’s health behavior change? An evidence review. J Med Internet Res.

[55] Michie S, van Stralen MM, West R (2011). The behaviour change wheel: a new method for characterising and designing behaviour change interventions. Implement Sci.

[56] Atkins L, Francis J, Islam R, O'Connor D, Patey A, Ivers N (2017). A guide to using the theoretical domains framework of behaviour change to investigate implementation problems. Implement Sci.

[57] Cane J, O’Connor D, Michie S (2012). Validation of the theoretical domains framework for use in behaviour change and implementation research. Implement Sci.

[58] Michie S, Wood CE, Johnston M, Abraham C, Francis JJ, Hardeman W (2015). Behaviour change techniques: the development and evaluation of a taxonomic method for reporting and describing behaviour change interventions (a suite of five studies involving consensus methods, randomised controlled trials and analysis of qualitative data). Health Technol Assess.

[59] Campbell M, Fitzpatrick R, Haines A, Kinmonth AL, Sandercock P, Spiegelhalter D (2000). Framework for design and evaluation of complex interventions to improve health. BMJ..

[60] Michie S, West R, Sheals K, Godinho CA (2018). Evaluating the effectiveness of behavior change techniques in health-related behavior: a scoping review of methods used. Transl Behav Med.

