# Supplementary material for: Knowledge translation strategies for dissemination with a focus on healthcare recipients: an overview of systematic reviews
Source: Implement Sci. 2020 Mar 4;15:14. doi: 10.1186/s13012-020-0974-3 (PMC7057470; doi:10.1186/s13012-020-0974-3)
Supplement: Supplementary file 1 — Additional file 1. Search terms and results. [file 13012_2020_974_MOESM1_ESM.docx]

## Additional file 1 - Search terms and results

### Keyword areas for searching

| **Keyword area** | **Search terms** |
| --- | --- |
| 1. Participants | Patient* OR consumer |
| 1. Intervention | “information dissemination” OR “competency-based education” OR “culturally competent care” OR “consumer health information” OR “consumer advocacy” OR “knowledge management” OR “dissemination strategy” OR “knowledge uptake” OR “knowledge transfer” |
| 1. Outcomes | (health knowledge, attitudes, practice) OR “patient education” OR “consumer behavior” OR “health literacy” OR “cultural competency” OR “communication barriers” OR (community-institutional relations) OR (Health Services Accessibility) OR “behavior change” OR “health behavior” OR barriers OR facilitators OR “research uptake” |
| 1. Systematic reviews | “systematic review” OR “meta analysis” OR MEDLINE |

Keyword areas were combined using AND

### Search results for each source

| **Database** | **Keyword areas searched** | **Date searched** | **No. refs found** | **No. refs after duplicates removed** |
| --- | --- | --- | --- | --- |
| **Electronic Databases** |  |  |  |  |
| CINAHL (EBSCOHost) | 1,2,3,4 | 21/5/18 | 32 | 26 |
| Embase (Ovid) | 1,2,3,4 | 21/5/18 | 392 | 331 |
| ERIC (EBSCOHost) | 1,2,4 | 21/5/18 | 7 | 7 |
| LILACS (BVSalud) | 2,4 | 21/5/18 | 59 | 58 |
| MEDLINE (Ovid) | 1,2,3,4 | 21/5/18 | 121 | 119 |
| PsycINFO (Ovid) | 1,2,4 | 23/5/18 | 26 | 13 |
| World Wide Science | 1,2,4 | 23/5/18 | 357 | 324 |
| ***Specialized sources of systematic reviews*** |  |  |  |  |
| Cochrane Database of Systematic Reviews | 2 | 21/5/18 | 14 | 14 |
| Database of Abstracts of Reviews of Effects (DARE) | 2 | 21/5/18 | 33 | 33 |
| Epistemonikos | 1,2 | 22/5/18 | 72 | 65 |
| Health Systems Evidence | 2 | 21/5/18 | 112 | 98 |
| Health Technology Assessment (HTA) | 2 | 21/5/18 | 29 | 28 |
| **Total:** |  |  | **1254** | **1116** |
| **Manual searches** |  |  |  |  |
| Google and Google Scholar  11 first pages | 1,2,3,4 | 20/01/19 | 18 | 18 |
| System for Information on Grey Literature in Europe (Open grey – <http://www.opengrey.eu>) | 2,4 | 23/05/18 | 12 | 12 |
| EPPI-Center Systematic Reviews (https://eppi.ioe.ac.uk/cms/Default.aspx?tabid=62) | NA |  | 292 | 292 |
| Rx for change (<https://www.cadth.ca/rx-change>) | 1 | 21/05/18 | 99 | 99 |
| 3ie - International Initiative for Impact Evaluation | 1 | 20/01/19 | 81 | 81 |
| **Total:** |  |  | **502** | **502** |
| **TOTAL:** |  |  |  | **1618** |

### Search strategies

**CINAHL – 21 May 2018**

| **#** | **Query** | **Results** |
| --- | --- | --- |
| **S5** | S1 AND S2 AND S3 AND S4 | **32** |
| **S4** | MW patient* OR MW consumer | 575,362 |
| **S3** | MW systematic review OR MW meta analysis OR AB MEDLINE | 98,105 |
| **S2** | MW health knowledge OR MW health attitudes OR MW health practice OR MW patient education OR MW (consumer behavior or consumer behaviour ) OR MW health literacy OR MW cultural competency OR MW communication barriers OR MW community-institutional relations OR MW health services accessibility OR MW (behaviour change OR behavior change) OR MW (health behavior OR health behaviour) OR MW research uptake | 189,134 |
| **S1** | MW information dissemination OR MW competency-based education OR MW culturally competent care OR MW consumer health information OR MW consumer advocacy OR MW knowledge management OR MW dissemination strategies OR MW knowledge uptake OR MW knowledge transfer | 18,470 |

**EMBASE (Ovid) – 21 May 2018**

Database: Embase Classic+Embase <1947 to 2018 May 18>

Search Strategy:

--------------------------------------------------------------------------------

1 information dissemination.mp. or exp Information Dissemination/ (19318)

2 competency-based education.mp. or exp Competency-Based Education/ (81218)

3 culturally competent care.mp. or exp Culturally Competent Care/ (5055)

4 consumer health information.mp. or Consumer Health Information/ (3750)

5 consumer advocacy.mp. or exp Consumer Advocacy/ (3169)

6 knowledge management.mp. or Knowledge Management/ (2333)

7 knowledge uptake.mp. (85)

8 knowledge transfer.mp. (1777)

9 dissemination strategy.mp. (180)

10 1 or 2 or 3 or 4 or 5 or 6 or 7 or 8 or 9 (115333)

11 health knowledge, attitudes, practice.mp. or exp Health Knowledge, Attitudes, Practice/ (101421)

12 exp PATIENT EDUCATION AS TOPIC/ or exp PATIENT EDUCATION HANDOUT/ (103755)

13 (consumer behavior or consumer behaviour).mp. or exp Consumer Behavior/ (4148)

14 health literacy.mp. or exp Health Literacy/ (10171)

15 cultural competency.mp. or exp Cultural Competency/ (5767)

16 communication barriers.mp. or exp Communication Barriers/ (1286)

17 community-institutional relations.mp. (24)

18 health services accessibility.mp. or exp Health Services Accessibility/ (2830343)

19 (behavior change or behaviour change).mp. (37091)

20 (health behavior or health behaviour).mp. or exp Health Behavior/ (364618)

21 barriers.mp. (129845)

22 facilitators.mp. (13265)

23 research uptake.mp. (73)

24 11 or 12 or 13 or 14 or 15 or 16 or 17 or 18 or 19 or 20 or 21 or 22 or 23 (3304999)

25 Patient*.mp. (9439316)

26 consumer.mp. (81393)

27 25 or 26 (9499422)

28 (systematic review or meta-analysis or MEDLINE).mp. (403421)

29 10 and 24 and 27 and 28 (**392**)

***************************

**ERIC – 21 May 2018**

| **#** | **Query** | **Results** |
| --- | --- | --- |
| **S5** | S1 AND S3 AND S4 | **7** |
| **S4** | TI ( patient* OR consumer ) OR AB ( patient* OR consumer ) OR SU ( patient* OR consumer ) | 29,959 |
| **S3** | TI ( systematic review OR meta analysis ) OR AB ( systematic review OR meta analysis OR MEDLINE ) OR SU ( systematic review OR meta analysis OR MEDLINE) | 7,382 |
| **S2** | TI ( health knowledge OR health attitudes OR health practice OR patient education OR (consumer behavior or consumer behaviour ) OR health literacy OR cultural competency OR communication barriers OR community-institutional relations OR health services accessibility OR (behaviour change OR behavior change) OR (health behavior OR health behaviour) OR research uptake ) OR AB ( health knowledge OR health attitudes OR health practice OR patient education OR (consumer behavior or consumer behaviour ) OR health literacy OR cultural competency OR communication barriers OR community-institutional relations OR health services accessibility OR (behaviour change OR behavior change) OR (health behavior OR health behaviour) OR research uptake ) OR SU ( health knowledge OR health attitudes OR health practice OR patient education OR (consumer behavior or consumer behaviour ) OR health literacy OR cultural competency OR communication barriers OR community-institutional relations OR health services accessibility OR (behaviour change OR behavior change) OR (health behavior OR health behaviour) OR research uptake ) | 28,546 |
| **S1** | TI ( information dissemination OR competency-based education OR culturally competent care OR consumer health information OR consumer advocacy OR knowledge management OR dissemination strategies OR knowledge uptake OR knowledge transfer ) OR AB ( information dissemination OR competency-based education OR culturally competent care OR consumer health information OR consumer advocacy OR knowledge management OR dissemination strategies OR knowledge uptake OR knowledge transfer ) OR SU ( information dissemination OR competency-based education OR culturally competent care OR consumer health information OR consumer advocacy OR knowledge management OR dissemination strategies OR knowledge uptake OR knowledge transfer ) | 28,998 |

**LILACS – 21 May 2018**

(tw:("information dissemination" OR "competency-based education" OR "culturally competent care" OR "consumer health information" OR "consumer advocacy" OR "knowledge management" OR "dissemination strategy" OR "knowledge uptake" OR "knowledge transfer")) AND (tw:("systematic review" OR "meta analysis" OR medline )) AND (instance:"regional") AND ( db:("LILACS"))

**MEDLINE (Ovid) – 21 May 2018**

Database: Ovid MEDLINE(R) Epub Ahead of Print, In-Process & Other Non-Indexed Citations, Ovid MEDLINE(R) Daily and Ovid MEDLINE(R) <1946 to Present>

Search Strategy:

--------------------------------------------------------------------------------

1 information dissemination.mp. or exp Information Dissemination/ (14984)

2 competency-based education.mp. or exp Competency-Based Education/ (3730)

3 culturally competent care.mp. or exp Culturally Competent Care/ (1247)

4 consumer health information.mp. or Consumer Health Information/ (3484)

5 consumer advocacy.mp. or exp Consumer Advocacy/ (3345)

6 knowledge management.mp. or Knowledge Management/ (1214)

7 knowledge uptake.mp. (52)

8 knowledge transfer.mp. (1337)

9 dissemination strategy.mp. (145)

10 1 or 2 or 3 or 4 or 5 or 6 or 7 or 8 or 9 (28852)

11 health knowledge, attitudes, practice.mp. or exp Health Knowledge, Attitudes, Practice/ (96253)

12 exp PATIENT EDUCATION AS TOPIC/ or exp PATIENT EDUCATION HANDOUT/ (84438)

13 (consumer behavior or consumer behaviour).mp. or exp Consumer Behavior/ (20244)

14 health literacy.mp. or exp Health Literacy/ (7520)

15 cultural competency.mp. or exp Cultural Competency/ (5151)

16 communication barriers.mp. or exp Communication Barriers/ (6369)

17 community-institutional relations.mp. (10250)

18 health services accessibility.mp. or exp Health Services Accessibility/ (100233)

19 (behavior change or behaviour change).mp. (11768)

20 (health behavior or health behaviour).mp. or exp Health Behavior/ (286424)

21 barriers.mp. (111083)

22 facilitators.mp. (10711)

23 research uptake.mp. (60)

24 11 or 12 or 13 or 14 or 15 or 16 or 17 or 18 or 19 or 20 or 21 or 22 or 23 (631861)

25 Patient*.mp. (6226137)

26 consumer.mp. (67030)

27 25 or 26 (6275890)

28 (systematic review or meta-analysis or MEDLINE).mp. (250552)

29 10 and 24 and 27 and 28 (**121**)

***************************

**PsycINFO (Ovid) – 23 May 2018**

Database: PsycINFO <1806 to May Week 2 2018>

Search Strategy:

--------------------------------------------------------------------------------

1 information dissemination.mp. or exp Information Dissemination/ (1885)

2 competency-based education.mp. or exp Competency-Based Education/ (194)

3 culturally competent care.mp. or exp Culturally Competent Care/ (359)

4 consumer health information.mp. or Consumer Health Information/ (112)

5 consumer advocacy.mp. or exp Consumer Advocacy/ (128)

6 knowledge management.mp. or Knowledge Management/ (4735)

7 knowledge uptake.mp. (21)

8 knowledge transfer.mp. (3349)

9 dissemination strategy.mp. (60)

10 1 or 2 or 3 or 4 or 5 or 6 or 7 or 8 or 9 (10095)

11 health knowledge, attitudes, practice.mp. or exp Health Knowledge, Attitudes, Practice/ (5)

12 exp PATIENT EDUCATION AS TOPIC/ or exp PATIENT EDUCATION HANDOUT/ (0)

13 (consumer behavior or consumer behaviour).mp. or exp Consumer Behavior/ (27985)

14 health literacy.mp. or exp Health Literacy/ (3540)

15 cultural competency.mp. or exp Cultural Competency/ (1248)

16 communication barriers.mp. or exp Communication Barriers/ (869)

17 community-institutional relations.mp. (2)

18 health services accessibility.mp. or exp Health Services Accessibility/ (71)

19 (behavior change or behaviour change).mp. (18029)

20 (health behavior or health behaviour).mp. or exp Health Behavior/ (29945)

21 barriers.mp. (50436)

22 facilitators.mp. (7520)

23 research uptake.mp. (19)

24 11 or 12 or 13 or 14 or 15 or 16 or 17 or 18 or 19 or 20 or 21 or 22 or 23 (128566)

25 Patient*.mp. (683209)

26 consumer.mp. (54494)

27 25 or 26 (734926)

28 (systematic review or meta-analysis or MEDLINE).mp. (46217)

29 10 and 24 and 27 and 28 (11)

30 10 and 27 and 28 (**26**)

***************************

**World Wide Science – 23 May 2018**

Title: knowledge translation AND systematic review AND (patient OR user)

Limit to: articles

N= **357** papers

***Specialized sources of systematic reviews***

**Cochrane Library (Cochrane Database of Systematic Reviews, DARE, HTA) – 21 May 2018**

"information dissemination" or "competency-based education" or "culturally competent care" or "consumer health information" or "consumer advocacy" or "knowledge management" or "dissemination strategy" or "knowledge uptake" or "knowledge transfer":ti,ab,kw (Word variations have been searched)

**Epistemonikos – 22 May 2018**

(title:(information dissemination) OR abstract:(information dissemination)) OR (title:(competency-based education) OR abstract:(competency-based education)) OR (title:(culturally competent care) OR abstract:(culturally competent care)) OR (title:(consumer health information) OR abstract:(consumer health information)) OR (title:(consumer advocacy) OR abstract:(consumer advocacy)) OR (title:(knowledge management) OR abstract:(knowledge management)) OR (title:(dissemination strategies) OR abstract:(dissemination strategies)) OR (title:(knowledge uptake) OR abstract:(knowledge uptake)) OR (title:(knowledge transfer) OR abstract:(knowledge transfer)) AND ((title:(patient*) OR abstract:(patient*)) OR (title:(consumer) OR abstract:(consumer)))

Filters: Publication Type – systematic review N=69; Publication Type – broad synthesis N=3

Total N=**72**

**Health Systems Evidence – 21 May 2018**

"information dissemination" OR "competency-based education" OR "culturally competent care" OR "consumer health information" OR "consumer advocacy" OR "knowledge management" OR "dissemination strategy" OR "knowledge uptake" OR "knowledge transfer"

Filters:

Consumer-targeted strategy:

- Information or education provision
- Behaviour change support
- Skills and competencies development
- (Personal) Support
- Communication and decision-making facilitation
- System participation

Document Type:

- Overviews of systematic reviews
- Systematic reviews of effects
- Systematic reviews addressing other questions

N=**112**
