# Supplementary material for: Knowledge translation strategies for dissemination with a focus on healthcare recipients: an overview of systematic reviews
Source: Implement Sci. 2020 Mar 4;15:14. doi: 10.1186/s13012-020-0974-3 (PMC7057470; doi:10.1186/s13012-020-0974-3)
Supplement: Supplementary file 2 — Additional file 2. Evidence rating scheme (based on Ryan et al. 2014 [9]). [file 13012_2020_974_MOESM2_ESM.docx]

## Additional file 2: Evidence rating scheme (based on Ryan et al. 2014^[[1]](#footnote-1)^)

| **Summary statement** | **Translation** |
| --- | --- |
| Sufficient evidence | Evidence to make a decision about the effect of the intervention(s) in relation to a specific outcome(s). This includes evidence of an effect in terms of (i) benefit or (ii) harm. Statistically significant results are considered to represent sufficient evidence on which to base decisions, but a judgement of sufficient evidence is also made based on the number of studies/participants included in the analysis for a particular outcome. A rating of sufficient evidence is often based on meta-analysis producing a statistically significant pooled result that is based on a large number of included studies/participants. This judgement may also be made based on the number of studies and/or study participants showing a statistically significant result – for example (in a narrative synthesis) a result where 12 studies of a total of 14 for a specific outcome showed a statistically significant effect of an intervention would be considered to represent sufficient evidence. |
| Some evidence | Less conclusive evidence to make a decision about the effects of a particular intervention(s) in relation to a specific outcome(s). This may be based on narrative syntheses of review results. In this case, the result is qualified according to the findings of the review - for example, ‘some evidence (5 studies of 9) reported a positive effect of .....’ (This would be based on a more equivocal set of results than those obtained for ’sufficient evidence’ above. For example, while 12/14 statistically significant studies would be classed as ’sufficient evidence’, 5/9 statistically significant studies is more equivocal and would be classed as ‘some evidence’). This may also be based on a statistically significant result obtained in a small number of studies; a statistically significant result obtained from studies with a small number of participants; or a statistically significant result obtained from studies of low quality. |
| Insufficient evidence | Not enough evidence to support decisions about the effects of the intervention(s) on the basis of the included studies. This should be interpreted as ‘no evidence of effect’, rather than ’evidence of no effect’. Statistically non-significant results are considered to represent insufficient evidence. Where the number of studies is small, and/or the number of participants included in the studies is small, insufficient evidence might reflect underpowering of the included studies to be able to detect an effect of the intervention. Where the number of studies is large, and/or the number of participants included in these studies is large, ’insufficient evidence’ may reflect underlying ineffectiveness of the intervention to affect the outcomes being examined. In such cases the intervention may additionally be described as ‘generally ineffective’ in order to separate such results from those cases where insufficient evidence is used to describe results but this is based on a small number of studies and/or participants (where non-significant results may reflect underpowering of studies rather than ineffectiveness). |
| Insufficient evidence to determine | Not enough evidence to be able to determine whether an intervention is effective or not on the basis of the included studies. This statement is about reporting gaps in the evidence (i.e. where there are too few studies to be able to determine effects), rather than the situation of the summary statement above, which is about ineffectiveness (e.g. several studies reporting a statistically non-significant result). It is likely to arise when the numbers of included studies is very small. |

1. Ryan R, Santesso N, Lowe D, Hill S, Grimshaw J, Prictor M, et al. Interventions to improve safe and effective medicines use by consumers: An overview of systematic reviews. Cochrane Database of Systematic Reviews. 2014;4:CD007768. [↑](#footnote-ref-1)
