# Supplementary material for: Knowledge translation strategies for dissemination with a focus on healthcare recipients: an overview of systematic reviews
Source: Implement Sci. 2020 Mar 4;15:14. doi: 10.1186/s13012-020-0974-3 (PMC7057470; doi:10.1186/s13012-020-0974-3)
Supplement: Supplementary file 3 — Additional file 3 Excluded studies (N = 47). [file 13012_2020_974_MOESM3_ESM.docx]

##

## Additional file 3. Excluded studies (N=47)

| **Study** | **Reason for exclusion** |
| --- | --- |
| Abaza H, Marschollek M. mHealth application areas and technology combinations. A comparison of literature from high and low/middle income countries. Methods of Information in Medicine. 2017;56(7):e105-e22. | Low quality |
| Abhyankar P, Volk RJ, Blumenthal-Barby J, Bravo P, Buchholz A, Ozanne E, et al. Balancing the presentation of information and options in patient decision aids: an updated review. BMC Med Inf Decis Mak. 2013;13 Suppl 2:S6. | Not a systematic review |
| Aranda-Jan CB, Mohutsiwa-Dibe N, Loukanova S. Systematic review on what works, what does not work and why of implementation of mobile health (mHealth) projects in Africa. BMC Public Health. 2014;14(188). | Low quality |
| Atherton H, Huckvale C, Car J. Communicating health promotion and disease prevention information to patients via email: a review. J Telemed Telecare. 2010;16(4):172-5. | Low quality |
| Balas EA, Weingarten S, Garb CT, Blumenthal D, Boren SA, Brown GD. Improving preventive care by prompting physicians. Arch Intern Med. 2000;160(3):301-8. | Not a focus of our overview |
| Balatsoukas P, Kennedy CM, Buchan I, Powell J, Ainsworth J. The Role of Social Network Technologies in Online Health Promotion: A Narrative Review of Theoretical and Empirical Factors Influencing Intervention Effectiveness. J Med Internet Res. 2015;17(6):e141. | Low quality |
| Barac R, Stein S, Bruce B, Barwick M. Scoping review of toolkits as a knowledge translation strategy in health. 2014. | Low quality |
| Barello S, Triberti S, Graffigna G, Libreri C, Serino S, Hibbard J, et al. eHealth for patient engagement: A systematic review. Frontiers in Psychology. 2015;6:2013. | Low quality |
| Bosch-Capblanch X, Abba K, Prictor M, Garner P. Contracts between patients and healthcare practitioners for improving patients' adherence to treatment, prevention and health promotion activities. Cochrane Database Syst Rev. 2007(2):CD004808. | Not a focus of our overview |
| Buhi ER, Trudnak TE, Martinasek MP, Oberne AB, Fuhrmann HJ, McDermott RJ. Mobile phone-based behavioural interventions for health: A systematic review. Health Education Journal. 2012;72(5):564-83. | Low quality |
| Chan Y, Nagurka R, Bentley S, Ordonez E, Sproule W. Medical utilization of kiosks in the delivery of patient education: a systematic review (Provisional abstract). Database of Abstracts of Reviews of Effects [Internet]. 2014; (2):[1-8 pp.]. Available from: http://cochranelibrary-wiley.com/o/cochrane/cldare/articles/DARE-12014050174/frame.html. | Not a systematic review |
| Cornet VP, Holden RJ. Systematic review of smartphone-based passive sensing for health and wellbeing. J Biomed Inform. 2018;77:120-32. | Low quality |
| Coylewright M, Branda M, Inselman JW, Shah N, Hess E, LeBlanc A, et al. Impact of sociodemographic patient characteristics on the efficacy of decision AIDS: a patient-level meta-analysis of 7 randomized trials. Circ Cardiovasc Qual Outcomes. 2014;7(3):360-7. | Not a systematic review |
| Coylewright M, Branda M, Shah N, Hess E, LeBlanc A, Montori V, et al. Shared decision-making results in knowledge transfer across diverse patient subgroups: An encounter-level meta-analysis of decision aid trials. Journal of the American College of Cardiology. 2012:E1847. | Low quality |
| Diviani N, van den Putte B, Giani S, van Weert JC. Low health literacy and evaluation of online health information: a systematic review of the literature. J Med Internet Res. 2015;17(5):e112. | Low quality |
| Donkin L, Christensen H, Naismith SL, Neal B, Hickie IB, Glozier N. A systematic review of the impact of adherence on the effectiveness of e-therapies. J Med Internet Res. 2011;13(3):e52. | Low quality |
| Fagerlin A, Ubel PA, Smith DM, Zikmund-Fisher BJ. Making numbers matter: present and future research in risk communication. American Journal of Health Behavior. 2007;31:S47-56. | Not a systematic review |
| Farrell EH, Whistance RN, Phillips K, Morgan B, Savage K, Lewis V, et al. Systematic review and meta-analysis of audio-visual information aids for informed consent for invasive healthcare procedures in clinical practice. Patient Educ Couns. 2014;94(1):20-32. | Is not a focus of our overview |
| Feldman-Stewart D, O'Brien MA, Clayman ML, Davison BJ, Jimbo M, Labrecque M, et al. Providing information about options in patient decision aids. BMC Med Inf Decis Mak. 2013;13 Suppl 2:S4. | Not a systematic review |
| Fernandez-Luque L, Staccini P. All that Glitters Is not Gold: Consumer Health Informatics and Education in the Era of Social Media and Health Apps. Findings from the Yearbook 2016 Section on Consumer Health Informatics. Yearb. 2016(1):188-93. | Not a systematic review |
| Gagliardi AR, Brouwers MC, Palda VA, Lemieux-Charles L, Grimshaw JM. How can we improve guideline use? A conceptual framework of implementability. 2011. | Not a systematic review |
| Gurman TA, Rubin SE, Roess AA. Effectiveness of mHealth behavior change communication interventions in developing countries: a systematic review of the literature. J Health Commun. 2012;17 Suppl 1:82-104. | Low quality |
| Hall CS, Fottrell E, Wilkinson S, Byass P. Assessing the impact of mHealth interventions in low- and middle-income countries - what has been shown to work? Global Health Action. 2014;7(0). | Low quality |
| Hardiker NR, Grant MJ. Factors that influence public engagement with eHealth: A literature review. Int J Med Inf. 2011;80(1):1-12. | Low quality |
| Higgins O, Sixsmith J, Barry MM, Domegan C. A literature review on health information-seeking behaviour on the web: a health consumer and health professional perspective. Stockholm: ECDC; 2011. | Not a systematic review |
| Horvat L, Horey D, Romios P, Kis-Rigo J. Cultural competence education for health professionals. Cochrane Database Syst Rev. 2014(5):CD009405. | Is not a focus of our overview |
| Househ M, Borycki E, Kushniruk A. Empowering patients through social media: the benefits and challenges. Health Inform J. 2014;20(1):50-8. | Not a systematic review |
| Ishikawa H, Yano E. Patient health literacy and participation in the health-care process. Health Expect. 2008;11(2):113-22. | Not a systematic review |
| Kim H, Xie B. Health literacy in the eHealth era: A systematic review of the literature. Patient Education and Counseling. 2017;100(6):1073-82. | Low quality |
| Kinnersley P, Phillips K, Savage K, Kelly MJ, Farrell E, Morgan B, et al. Interventions to promote informed consent for patients undergoing surgical and other invasive healthcare procedures. Cochrane Database Syst Rev. 2013;2013(7):1-247. | Is not a focus of our overview |
| Krishna S, Balas EA, Boren SA, Maglaveras N. Patient acceptance of educational voice messages: a review of controlled clinical studies. Methods Inf Med. 2002;41(5):360-9. | Not a systematic review |
| Kurtzman ET, Greene J. Effective presentation of health care performance information for consumer decision making: A systematic review. Patient Educ Couns. 2016;99(1):36-43. | Not a systematic review |
| Lewis D. Computer-based approaches to patient education: a review of the literature. Journal of the American Medical Informatics Association : JAMIA. 1999;6(4):272-82. | Not a systematic review |
| Lewis D. Computers in patient education. Comput Inform Nurs. 2003;21(2):88-96. | Not a systematic review |
| Lin H, Wu X. Intervention strategies for improving patient adherence to follow-up in the era of mobile information technology: a systematic review and meta-analysis. PLoS ONE. 2014;9(8):e104266. | Is not a focus of our overview |
| Lundahl B, Moleni T, Burke BL, Butters R, Tollefson D, Butler C, et al. Motivational interviewing in medical care settings: a systematic review and meta-analysis of randomized controlled trials. Patient Educ Couns. 2013;93(2):157-68. | Is not a focus of our overview |
| Nhavoto JA, Gronlund A. Mobile technologies and geographic information systems to improve health care systems: A literature review. JMIR mHealth and uHealth. 2014;2(2):e21. | Low quality |
| Or CK, Karsh BT. A systematic review of patient acceptance of consumer health information technology. J Am Med Inform Assoc. 2009;16(4):550-60. | Low quality |
| Sadowski C, Hussain G. Evaluating readability and legibility of patient health information: A review of available tools. Canadian Pharmacists Journal. 2017;150 (4):S40. | Not a systematic review |
| Santo A, Laizner AM, Shohet L. Exploring the value of audiotapes for health literacy: A systematic review. Patient Education and Counseling. 2005;58(3 SPEC. ISS.):235-43. | Low quality |
| Shahmoradi L, Safadari R, Jimma W. Knowledge Management Implementation and the Tools Utilized in Healthcare for Evidence-Based Decision Making: A Systematic Review. Ethiop. 2017;27(5):541-58. | Low quality |
| Smith CA. Consumer language, patient language, and thesauri: a review of the literature. J Med Libr Assoc. 2011;99(2):135-44. | Not a systematic review |
| Staccini P, Fernandez-Luque L. Health Social Media and Patient-Centered Care: Buzz or Evidence? Findings from the Section "Education and Consumer Health Informatics" of the 2015 Edition of the IMIA Yearbook. Yearb. 2015;10(1):160-3. | Not a systematic review |
| Theis SL, Johnson JH. Strategies for teaching patients: a meta-analysis. Clin Nurse Spec. 1995;9(2):100-5, 20. | Low quality |
| Tugwell P, Robinson V, Grimshaw J, Santesso N. Systematic reviews and knowledge translation. Bull World Health Organ. 2006;84(8):643-51. | Not a systematic review |
| Wallace J, Byrne C, Clarke M. Improving the uptake of systematic reviews: a systematic review of intervention effectiveness and relevance. BMJ Open. 2014;4(10):e005834. | Is not a focus of our overview |
| Ye J, Rust G, Fry-Johnson Y, Strothers H. E-mail in patient-provider communication: A systematic review. Patient Education and Counseling. 2010;80(2):266-73. | Not a systematic review |
