# Supplementary material for: Knowledge translation strategies for dissemination with a focus on healthcare recipients: an overview of systematic reviews
Source: Implement Sci. 2020 Mar 4;15:14. doi: 10.1186/s13012-020-0974-3 (PMC7057470; doi:10.1186/s13012-020-0974-3)
Supplement: Supplementary file 4 — Additional file 4. Characteristics of included studies and AMSTAR quality assessment. Table 1 Characteristics of included systematic reviews. Table 2 Quality of included systematic reviews (AMSTAR). [file 13012_2020_974_MOESM4_ESM.docx]

## Additional file 4: Characteristics of included studies and AMSTAR quality assessment

### Table 1. Characteristics of included systematic reviews

| **Author** | **Topic (Objectives)** | **Informed by theory / framework** | **Study designs included and number** | **Date of most recent search** | **Interventions*** | **Participants** | **Settings** | **Country(ies)** | **Financing source** |
| --- | --- | --- | --- | --- | --- | --- | --- | --- | --- |
| Abu Abed 2014 | Evaluate the efficacy of video-assisted patient education to modify behavior. | Yes | 20 randomized control trials | October 2013 | Video-assisted patient education to modify behavior. Variable in length, content and structure | The articles addressed 12 different diseases or health-related problems. | Video-watching at the study site or at home. | not described | None |
| Akesson 2006 | To describe consumers’ subjective experiences of electronic information and communication resources with reference to health and illness. |  | 12 studies of medium and high quality. Seven quantitative (2 descriptive and 5 RCT) and 5 qualitative studies. | April 2004 | e-mail 12 months, Computer 5-10 min/year, Telecommunication weekly 1 year | Strategy 1: 98 participants: families with very low birth weight babies; elderlies' families and/or carriers; rural women with breast cancer, patients suffering from dementia. Strategy 1: 2872 participants: oncology patients and families; diabetic patients; breast-feeding women; patients with back pain.  Strategy 3: 1378 participants: retirement home nurses and patients; hypertensive patients; diabetic and non-diabetic children. | No details about settings. Were outpatients in general and with multiple conditions. | (1) support and help: 5 studies (1 USA, 1 England, 1 Sweden, 1 Canada, 1 Finland), (2) education and information: 4 studies (2 USA, 1 Canada),  (3) telecommunication instead of on-site visiting:3 studies (USA) | Kalmar e-Health Institute and the Department of Health and Behavioral Science, Kalmar University, Sweden |
| Akl 2011a^[[1]](#footnote-1)^ | To evaluate the effects of attribute (positive versus negative) framing and of goal (gain versus loss) framing of the same health information, on understanding, perception of effectiveness, persuasiveness, and behavior of providers, policy makers, and consumers. | Yes | 35 studies and 51 comparisons that included: Randomized controlled trials (44), quasi-randomized controlled trials (8),and cross-over study (1) | October 2007 | Positively or negatively framed and targeted messages. | 16,342 participants (all health consumers). Topics: Cancer, sexual education, diet, prevention behaviors and high cholesterol. | Variables and included: graduate and undergraduate students, adults healthy, employees, low income neighborhoods, volunteers, beach goers, genetic counselling services, health clinics, women with abnormal Pap test or mammograms, patients with colorectal cancer or breast cancer, outpatient respiratory and cardiac clinics. | Not reported. | None |
| Akl 2011b^[[2]](#footnote-2)^ | To evaluate the effects of using alternative statistical presentations of the same risks and risk reductions on understanding, perception, persuasiveness and behaviour of health professionals, policy makers, and consumers. |  | 35 studies reporting 83 comparisons. Randomized and non-randomized controlled parallel and cross-over studies were included. No study was conducted with policy makers. | October 2007 | Statistical format in hypothetical scenario. Same evidence was presented in different formats to participants (Consumers, policy-makers and providers). Risk (frequencies, percentages and probabilities); Risk reduction (RRR, ARR, NNT) | 20 were conducted with health consumers, 14 with providers, and 1 with both. Consumers included patients, the general public, and students. Students of health professions were considered as consumers. | Hypothetical scenarios | Not consistently reported | Internal sources: State University of New York at Buffalo, NY, USA. Salary support, infrastructure; Italian National Cancer Institute, Regina Elena, Rome, Italy. - Salary support. External sources: Norwegian Research Council, Norway. - Salary support; HJS is funded by a European Commission: The human factor, mobility and Marie Curie Actions. Scientist Reintegration Grant: IGR 42194 - GRADE. Salary support |
| Ammentorp 2013 | To assess the health-related outcomes of life coaching interventions conducted with patients in the form of individual telephone coaching, individual face-to-face coaching, group coaching, or coaching that combines some or all of these methods. |  | Studies were intervention studies using quantitative or qualitative methods to evaluate the outcome of the coaching or a combination of the methods. 5 studies (6 articles) were included - 2 RCTs, 1 case study, 1 pre-post intervention study, and 1 intervention study (not clearly defined). | January 2013 | Coaching: 6 to 14 sessions. | Diabetes patients were the focus of the intervention in three, patients with spinocerebellar degeneration were in one study and cancer patients in the last study. Four were in adults and one study was in adolescents. | Not clear. Two studies used telephone for coaching, and three used both face-to-face and telephone coaching, with one of these studies also using group coaching. | 3 USA, 2 Japan, 1 Denmark | None declared. The authors declared that they have no competing interests. The affiliations of all authors are Universities. |
| Ammenwerth 2012 | Review the impact of electronic patient portals on patient care. |  | 5 articles presented evaluations of 4 different patient portals.  Study designs included 4 RCT and 1 retrospective matched- control study. | April 2012 | Portal, e-mail. 2 to 13 months | (1) 180 patients undergoing IVF treatment, (2) 244 diabetes mellitus patients, (3) 81 patients with congestive heart failure and (4) 6402 general patients in the portal. | Patients from 4 different health services that have internet access. | Portals were located in: Netherlands (1); USA (3) | Partly supported by the COMET Center Oncotyrol which is funded by the Austrian Federal Ministries of BMVIT/BMWFJ (via FFG) and the Tiroler Zukunftsstiftung/Standortagentur Tirol (SAT). |
| Atherton 2012 | To assess the effects of healthcare professionals and patients using email to communicate with each other, on patient outcomes, health service performance, service efficiency and acceptability. |  | Nine trials enrolling 1733 patients (Seven RCTs; two cluster-RCT) | July 2010 | e-mail to communicate | Sample sizes ranged from n = 16 to n = 606 participants. | Studies were conducted in a variety of healthcare settings across primary, secondary (outpatient settings) and tertiary care (perioperative surgical settings for head and neck surgery), and in the community (rehabilitation center). Two studies were in physicians. Eight were set in urban areas and one in a mixed urban/rural population. | All studies were conducted in high income countries: USA (5), Norway (2), Canada (1), Australia (1). | Internal sources:  Department of Primary Care and Public Health, Imperial College London, UK.  NIHR Collaboration  for Leadership in Applied Health Research & Care (CLAHRC) Scheme.  External sources:  Medical Research Council, UK.  NHS Connecting for Health Evaluation Programme (NHS CFHEP 001), UK.  National School of Primary Care Research, UK. |
| Bekker 2013 | To examine the evidence to support the addition of personal stories to patient decision aid interventions. | Yes | 11 articles reporting findings from 13 studies. Experimental (9 RCT), comparative no randomized (1) and quasi-experimental (1) studies comparing the effect of the PtDA intervention with and without a personal story component on people’s healthcare decision making. | 2005 to 2012 | PtDAs designed to inform and support experiences of healthcare | The informed decision making: 1688 participants of general population, 1694 students. The informed choice: 2022 (489 African- American women) participants, general population. The informed engagement: 450 (149, low literacy; 301, high literacy) patients eligible for prostate cancer screening, 100 female patients with early stage breast cancer, 200 general population; 14 pairs of patients (and their caregiver) of general population, 76 participants of general population and 50 patients with malignant glioma. | Screening for prostate, breast, and colorectal cancers; treatment decisions between angioplasty / bypass surgery, mastectomy / breast conserving surgery, peritoneal dialysis / hemodialysis; and end-of-life level of care in cancer and in dementia. PtDA interventions were delivered directly via access to web links or face-to-face with computer support. | The informed decision-making studies: 3 articles (2 USA, 1UK). The informed choice studies: 2 articles (USA). The informed engagement studies: 6 articles (USA) | Partially funded by an unrestricted grant from the Informed Medical Decisions Foundation, Leeds Institute of Health Sciences, School of Medicine, University of Leeds (Bekker). Administrative and editorial support was also provided by The University of Texas MD Anderson Cancer Center. |
| Berkman 2011 | This is a HTA. Review the relationship of health literacy to various outcomes and disparities and interventions to improve low health literacy. Key Question 1. Outcomes: Are health literacy skills related to use of health care services, health outcomes, costs of health care, and disparities in health outcomes or health care service use? Key Question 2. Interventions: For individuals with low health literacy skills, what are the effective interventions? | Yes | 81 studies (95 articles) addressed Key Question 1 and 42 studies (45 articles) addressed Key Question 2. These 42 studies were of good- or fair-quality: 27 RCTs, 2 cluster randomized trials, and 13 quasi-experimental studies. | May 2010 | Interventions for health literacy using alternative document design or numerical presentation, additive or alternative pictorial representations, alternative media, and combination of alternative readability and document design. Counselling, flyers, labels, symbols, numerical information, tutorials. | The study populations have different proportions of individuals with low health literacy or low numeracy. Twenty-one studies examined the effect of interventions specifically in low-health-literacy subgroups. Other studies examined intervention effects in populations that included both low- and high-health-literacy or -numeracy individuals; these studies provide only supportive evidence about the effect of interventions to mitigate the effects of low literacy. | Primary care clinic, outpatient infectious disease clinic, urology clinic, university-based, urban tertiary care hospitals, nephrology clinic (public health clinic), two general medicine clinics/firms Institute for Human Development, households, urban outpatient clinics, family practice clinics affiliated with an urban academic teaching hospital, healthcare services, University-based graduate orthodontic clinics , diabetes education class at one Hospital, internal medicine outpatient clinics, cardiology clinic, pediatric allergy clinic, clinics in a community health network , diverse settings: a faith-based organization, an adult basic education center, and a general internal medicine ambulatory care clinic, participants recruited in 3 hospital rheumatology departments, urban emergency department. | Mostly from USA. Others countries: UK, Canada, Germany, New Zealand. Some studies not reported. | The Agency for Healthcare Research and Quality (AHRQ), USA |
| Büchter 2014 | The comparative effects of words versus numbers in communicating the probability of adverse effects or harms of treatments to consumers in written health information. |  | 10 studies (7 articles) - all RCTs. Many studies used a factorial design. | November 2012 | Words Vs numbers in risk communication (Hypothetical scenario). Leaflets on drugs for a particular condition | 1736 participants, all volunteers. In all but one study participants were recruited from the general population or via a cancer website and confronted with a hypothetical scenario. Participants had a variety of educational backgrounds and all were adults. | Hypothetical, fictional or real scenarios. Treatment effects were communicated through written health information only | UK | The authors did not receive any funding for this work apart from their salary. All authors are purveyors and proponents of evidence based consumer health information. |
| Car 2011 | To assess the effects of interventions for enhancing consumers’ online health literacy (skills to search, evaluate and use online health information). | Yes | Two studies, 1 RCT) and 1 controlled before and after (CBA) | March 2008. | Online health information addressing health literacy (skills to search, evaluate and use online health information). Groups of six to ten participants “Adult education style", discussions, 90 to 120 min for 4 weeks. One study had 9 months of follow-up | 470 participants. 1 RCT with 448 people living with HIV/AIDS (320 male; 116 female; 12 trans gender); (399 African American; 36 White American; 13 other) and 1 CBA with 22 healthy adults (18 female and 4 male). | Both studies took place in specialist computer canters placed in community venues in the USA. | USA (2) | Partial financial contribution from The Department of Primary Care and Public Health, Imperial College London. External sources: NHS Connecting for Health Evaluation Programme (NHS CFHEP 001), UK. |
| Cole-Lewis 2010 | To assess the effectiveness of behavior change interventions for disease management and prevention delivered primarily through text messaging | Yes | 12 studies represented by 17 articles. Nine RCT, 2 randomized crossover trials and one quasi-experimental trial. | June 2009 | Text messaging, 3 to 12 months, 5 per day to 1/weekly, uni and bidirectional | 2425 participants: 246 diabetic adolescents, 165 diabetic adults, 191 overweight adults, 1705 smokers, 16 asthmatics adults, 102 healthy adults. Gender was nearly equally distributed in most studies, with the exception of 3 studies in which females were greatly overrepresented or underrepresented. | General population in the disease prevention studies and from clinics in the disease management studies. Only one recruited healthy individuals, whereas the rest were targeted toward people with a specific disease or condition. | Canada (1), Finland (1), New Zealand (2), United States (2),France (1), South Korea (2), Scotland (1), Croatia (1), and Austria (1). | Supported by the National Institute of Mental Health (T32 MH020031) |
| Edwards 2000 | To assess whether risk-communication interventions are associated with changes in patient knowledge, attitudes, and behaviors, and to identify aspects of these interventions that modify these effects. |  | 51 studies were RCTs and 31 as non-RCTs. Note: study designs were not specified for inclusion criteria nor details given in results. 96 studies met inclusion criteria but only 82 were used in the meta-analysis. | 1996 | One to one risk communication (not necessarily face to face) | Of the 96 publications that fulfilled the inclusion criteria, 19 related to coronary heart disease risk, 15 to smoking, 14 to breast screening, 9 to HIV risk, 6 to accident prevention, 5 to cervical screening, and 28 others. | Settings included family practice, other primary care, secondary care, outreach (i.e., proactive seeking of patients), and workplace. | Not specified. | None specified. |
| Faber 2009 | The review assessed 3 main questions: 1. What is the weight given to quality-of-care information by consumers in the process of choice? 2. What is the effect of the presentation format on consumer choice based on quality-of-care information? 3. What is the influence of quality-of-care information on consumer choice in a real-world setting? | Yes | 12 RCT, 2 CBA | January 2008 | Quality of care information based on real or hypothetical performance. Three types (A, B, C). Report cards, mails, different formats (framing information, costs of health plan, highlighted information, etc.) | The participants were in a role of health care consumers, as opposed to patients seeking health care services. The number of participants ranged from 34 to 13,078 (the median number of participants was 316). | All studies were performed in the United States after 1993, the year in which the release of HEDIS (Health Plan Employer Data and Information Set) introduced the first systematic approach for public reporting of health plans. In 4 studies, the experiment was conducted in a real-world setting, with actual quality information and dissemination of the information by personal mailings. The other 10 studies used laboratory settings with hypothetical quality information. Except for 2 studies that used hospital quality-of-care information, settings with quality-of-care information within HPs were studied. | USA | None specified. All authors declared that they have no conflict of interest, including specific financial interests and relationships and affiliations relevant to the subject matter or material discussed in the manuscript. Affiliations of authors are Universities. |
| Finkelstein 2012 | The impact of health information technology (IT) that supports patient-centered care (PCC). Key Questions: Key Question 1. Are health IT applications effective in improving the following outcomes, and how do the outcomes vary by type of health IT application? Key Question 2. What are barriers or facilitators that may impact implementation and use of health IT applications to enable PCC? Key Question 3. What knowledge or evidence deficits exist regarding needed information to support estimates of cost, benefit, impact, sustainability, and net value with regard to enabling PCC through health IT? Key Question 4. What critical information regarding the impact of health IT applications implemented to enable PCC is needed to give consumers, their families, clinicians, and developers a clear understanding of the value proposition particular to them? |  | 327 articles that were applicable to Key Questions 1 and/or 2—184 RCTs for Key Question 1, 206 for Key Question 2, and 63 articles that were applicable to both Key Questions 1 and 2. The 206 articles addressing barriers and facilitators were for the most part RCTs, qualitative studies, and usability studies. KQ3 and KQ4: scarce evidence. | July 2010 | Health Information Technologies | Study populations have varied from as few as 10 patients to more than 1,000. The studies have targeted physicians, nurses, and patients and have used many different types of health IT. The interventions were addressed to many medical conditions. | KQ1a: Settings have included hospitals, outpatient practices, and patients’ homes.KQ2: Studies focused on a wide variety of clinical conditions, including diabetes mellitus, cardiovascular disease, heart failure, COPD, cancer, asthma, mental health, sickle cell disease, and chronic pain. | Australia(8),Austria(2),Belgium(1),Canada(15),China(2), Denmark(1), Finland(3), France(3), Germany(6), Greece(1), India(1), Israel(3), Italy(6), Japan(2), Korea (Republic of)(1), Netherlands(9), New Zealand(1), Norway(6), Portugal(1), Singapore(1),Spain(4), Sweden(2), Switzerland(1),Taiwan(2), England(31), Northern Ireland(2), Scotland(3, Wales(1), USA (191) | Agency for Healthcare Research and Quality U.S. Department of Health and Human Services |
| Fjeldsoe 2009 | Analyzes the application of SMS for delivering health behavior change interventions. |  | 14 studies (6 RCT, 1 a clustered randomized comparative trial, 1 randomized crossover trial, and six were pre–post design studies). | March 2008 | Tailored SMS messages. 6 to 12 months, e-mail, interactive Website, self-monitoring or accelerometer, brochures | Smokers, healthy adults, adults, adolescents and pediatric diabetes patients, asthma patients, patients with uncontrolled hypertension and patients diagnosed with bulimia nervosa. | Outpatient clinics, public health clinics, Healthcare Centers, pediatric clinic, diabetes clinics, hospitals. | New Zealand, Washington DC, Bedfordshire United Kingdom, Korea, Finland, Scottish. Vienna, Austria. South Korea, Croatia, Spain, Toronto, U.S and England | No financial disclosures were reported. |
| Gagliardi 2016 | To identify and describe effective strategies for Patient-mediated Knowledge Translation (PKT) during clinical encounters. | Yes | 12 RCT, 4 cohort studies | September 2014 | Patient mediated KT for clinical encounters. Including brochures, booklets, before or after consultation | 3767 adults participants. All were arthritis and cancer patients from primary care clinics, hospitals or clinics and living homes. | Clinical encounters (outpatients with arthritis or cancer) | USA (10), UK (2), Canada (1), China (1), France (1), Netherlands (1) | The Canadian Institutes of Health Research |
| Gibbons 2009 | 1) Impact of Consumers Health Information (CHI) applications on health outcomes, and 2) barriers that clinicians, developers, consumers, and their families or caregivers encounter that limit utilization or implementation of CHI applications.  Sub key Q1 outcomes: a) health care process outcomes, b) intermediate outcomes, c) relationship-centered outcomes, d) clinical outcomes. | Yes | 162 articles: 137 RCT for objective 1, 31 studies (24 non-validated surveys and 7 qualitative studies) for the objective 2, and 6 articles for both objective 1 and objective 2. | June 2009 | Consumer health informatic (CHI). Interactive Website-based app or Web-based tailored education Websites, Computer generated tailored feedback APP, interactive computer programs and personal monitoring devices | In terms of participant age groups, 77% (76/99) of studies reporting age of participants targeted adult CHI users. Approximately 12% of studies targeted adolescents/teens, 3% of studies targeted seniors and another 3% of studies targeted children, 5% of studies targeted participants from overlapping age groups. Of studies reporting the race of the participants 92% (49/53) of the studies employed populations that were greater than 50 percent white/Caucasian. There was only one study with greater than 50 percent African-American participants and no studies with a majority of participants who were Hispanic, American Indian/Alaska Native, or Asian/Pacific Islander. | In terms of intervention delivery setting or location, 58% of studies evaluated CHI applications that were used in the home or residence, a minority of evaluations were completed in schools (15%), clinical settings (17%), communities (3%), online (5%) or kiosks (2%). | Not reported | Agency for Healthcare Research and Quality, U.S. Department of Health and Human Services. |
| Health Quality Ontario 2013 | Examine the impact of eTools for health information exchange in the context of care coordination for individuals with chronic diseases in the community. |  | A total of 11 citations were included (4 randomized controlled trials and 7 observational studies) | April 2012 | Electronic Tools for Health Information Exchange, addressed to providers in the context of care coordination. Electronic medical records (eTools). Note: provider to provider, patients are indirectly involved | Mostly health service's staff in communication with other services attending different patients: with coronary artery disease (1); diabetes (7); heart failure (1); and multiple chronic conditions (2). | The eTools applied in each study were unique, as were the conditions under which they were applied.  Some were used to coordinate care between hospital-based and outpatient/community-based health care providers; some were applied in a community setting to help coordinate care between primary care physician and other health care professionals (e.g., nurses and pharmacists); the rest were applied in multiple care coordination efforts and/or did not specify their points of care coordination communication. | Australia (1),  Netherlands (1), United Kingdom (1), United States (8). | Ontario Government |
| Hoffman 2017 | Assess the ability of medical television programs to affect important public health outcomes such as viewers’ health-related knowledge, perceptions and/or behavior. | Yes | 19 studies (15 surveys, 4 longitudinal -2 RTC) Many included studies used convenience instead of random samples, because recruitment advertisements for studies involving medical television are likely to attract viewers who may have more favorable views of medical television. | February 2015 | Fictional television. Regular viewing habits | One study had adolescent participants, and the remaining eighteen had adult (18+) participants. Seven studies (37%) enrolled students from college undergraduate courses, and no studies assessed participants aged 65 or above. Not all studies reported the mean age of participants, for those who did, mean age was 29 years. The number of participants per study ranged from 35 to 11 555, with a mean of 1659  The percent of female participants ranged from 41 to 79%, excluding two studies that involved only one gender.  Thirteen studies reported participant race/ethnicity, all but one had a majority of Caucasian participants. | Eight studies (42%) used overall regular viewing habits as the exposure, and seven studies (37%) asked participants whether or not they saw specific episode(s) as the exposure. Three (16%) studies exposed respondents to a specific clip or episode in a classroom setting, and one study (5%) used both regular viewing habits and viewership of specific episodes to define the exposure. The scope was limited to shows that feature health professionals in a professional setting. | USA (19) | Not reported |
| Ketelaar 2011 | To estimate the effects of publicly releasing performance data on changing the behavior of three target groups: healthcare consumers (patients), providers of healthcare (health professionals) and purchasers of healthcare. |  | Total 4 studies (Cluster-randomized controlled trial; cluster quasi-randomized trial; interrupted time series and Cluster-randomized trial) | 2011 | Publicly releasing regarding performance data about any aspect of the healthcare organizations or individuals. Three target groups: healthcare consumers (patients), providers of healthcare (health professionals) and purchasers of healthcare. In written or electronic form, with varying degrees of accessibility, such as a report in a publicly accessible library or more active dissemination directly to consumers in newspapers, leaflets, personal mailings, broadcasting media, etc. | Medicaid beneficiaries Medicaid beneficiaries: 5878 (Age: unclear and gender: men and women). Patients: treated for CABG in New York, and for AMI and post discectomy complications in California (Number of patients: unclear, age: unclear -children younger than 18 years were excluded- and gender: men and women). 15997 patients treated for AMI or CHF (Age: no restriction and gender: men and women) | Two studies were set in health plans. One study set in hospitals in California and New York and one study set in hospitals in Canada.  One study was conducted in 35 of the 99 Iowa counties. The second study was based on the New Jersey Medicaid programme. The third study was based in California. In Canada, evaluated the public release of performance data of 12 process-of-care indicators for AMI and six indicators for congestive heart failure (CHF) in 86 hospitals. | Three studies were conducted in the USA and one study was conducted in Canada. | None |
| Kinnersley 2007 | To assess the effects on patients, clinicians and the healthcare system of interventions which are delivered before consultations, and which have been designed to help patients (and/or their representatives) address their information needs within consultations. |  | 33 RCT with 8244 patients. | September 2006 | Interventions before consultations | Patients and/or their representatives (or caregivers) of all ages before ’one-to-one’ consultations with doctors or nurses in healthcare settings. Excluded: Individuals or groups attending activities such as health promotion clinics (for example, antenatal classes) or in-patients for whom there were not specific subsequent identifiable consultations. Individuals consulting other healthcare professionals. | Primary care and hospital settings among patient with various conditions (number of studies): cancer (9), diabetes (2), cardiac problems (2), obstetric or gynecological problems (2), mixed outpatients (1), women attending family planning clinics (1), women attending a well-baby clinic (1), children attending a pediatric clinic (1) and one on patients with peptic ulcers. In the study conducted in a pediatric setting, both children and their parents received interventions. Thirty studies reported on patients consulting physicians, two on patients consulting either physicians or nurses, and one on family planning care providers. | USA (18), Netherlands (2), Australia (4), Canada (2), UK (7), Indonesia (1) | Grant from the PPP Foundation (now known as the Health Foundation), Australia. Internal sources: Cardiff University, UK. |
| Laranjo 2014 | Effectiveness of interventions using social networking sites (SNSs) to change health behaviors | Yes | 12 studies, 7411 participants. Three quasi-experimental and 9 RCT. | March 2013 | Social networking (Facebook, Twitter), websites, + email. 21 days to 18 months | Not all studies reported age data, 3 recruited students and 2 involved young adults | The health domains covered were fitness, sexual health, food safety, smoking, and health promotion. | Australia (1), UK (1) and USA(10) | The Harvard Medical School-Portugal program (HMSP-ICJ/0005/2010; National Health and Medical Research Council (NHMRC) Centre of Research Excellence in Informatics and E-Health and the Portuguese Foundation for Science and Technology. |
| Loudon 2014 | Identify and synthesize evidence of the public’s attitudes towards clinical practice guidelines and evidence-based recommendations written for providers or the public, together with their awareness of guidelines. |  | 26 studies (10 qualitative studies, 13 cross sectional and 3 RCT) | January 2013 | CPG and evidence-based written recommendations including symbols and words | 24887 participants. The age of participants ranged from 30 to over 76 years and one study on 11–15-year-old adolescents. Most studies included both genders although some included only women because of the topic (e.g. breast cancer). | Primary care, patient groups or with a special condition, websites, including caregivers. For instance: Canadian office workers, female careers in Maryland, USA, Londoners attending drop-in centers in the UK for patients with mental health problems, visitors to a welfare center in Seoul, women attending secondary care for menstrual abnormalities in Leicestershire, UK, and patients with Diabetes in Australia. | Canada (6), UK (4), Seoul (1), USA (10), Australia (4), Israel (1) | European Community’s Seventh Framework Programme (FP7/2007-2013) under grant agreement n° 258583 (DECIDE project). |
| Maher 2014 | The effectiveness of online social network health behavior interventions. | Yes | 10 Studies. Three key study: (1) large-scale evaluations of “live” interventions, with >1000 participants (four studies with sample sizes ranging from 545 to 107,907), (2) medium-scale, tightly-controlled randomized controlled trials, with approximately 100 participants (four studies with sample sizes ranging from 52 to 134) and (3) small pilot studies, each with 10 participants (two studies). In all, five studies were randomized controlled trials (RCT), one was a randomized cross-over study, and four were single group pre-post studies. Of the six studies that utilized a separate control group or arm (crossover study), only one had a “true” (ie, no-intervention) control, with the others comparing the online social networking intervention with an alternative intervention (in five cases the alternative intervention was Web-based, and in three cases the alternative intervention involved an online social networking component). | December 12, 2012 | Social network and equipment (scales) | 113,988 participants. Adults or children regardless of health status (healthy or participants with specific health conditions or diseases). The studies typically reported high rates of female participation: on average 83.3% of participants were female. | Live trials (4), Community (2), University (2), Hospital nursing staff (1), and 1 not reported. The targeted health behaviors were diet/weight loss (n=2), physical activity (n=3), or a combination of diet/weight loss and physical activity (n=5). No eligible studies targeted smoking or alcohol consumption. Interventions included commercial online health social network websites (n=2), research health social network websites (n=3), and multi-component interventions delivered in part via pre-existing popular online social network websites (Facebook n=4 and Twitter n=1) | Australia (2), USA (5), Japan (2), UK (1) | University of South Australia Fellowship Support |
| Mc Cormack 2010 | Communication and dissemination strategies to facilitate the use of health-related evidence. The review focused on three primary objectives—comparing the effectiveness of: (1) communicating evidence in various contents and formats that increase the likelihood that target audiences will both understand and use the information (KQ 1); (2) a variety of approaches for disseminating evidence from those who develop it to those who are expected to use it (KQ 2); and (3) various ways of communicating uncertainty-associated health-related evidence to different target audiences (KQ 3). A secondary objective was to examine how the effectiveness of communication and dissemination strategies varies across target audiences, including evidence translators, health educators, patients, and clinicians. | Yes | 61 articles that directly (i.e., head to head) compared strategies to communicate and disseminate evidence. Nine articles (representing 7 RCT) were to KQ 1; 42 articles (representing 38 RCT) were relevant to KQ 2 and 21/38 relevant to public/patients; 10 articles (representing 2 RCT,4 factorial RCT, 2 quasi-experimental and 1 noncontrolled trial) were relevant to KQ 3. | March 2013 | Communication and dissemination strategies. Face to Face, electronic sources and equipment (Scales). Narrative. Framing, Tailored and targeted strategies | Adults (≥19 years), general public and patients, clinicians, children (<19 years), incarcerated populations, federal and state policymakers KQ1: Total 10,145 participants, KQ2: 14970 participants from 21 studies addressed to public/patients. KQ3: 5405 participants from 3 studies that included public/patients. | KQ1: Participants from community health clinics, public housing, university classrooms, low income neighborhoods, health care practice, women veterans and lay health workers.KQ2: participants from community-based, parents or primary caregivers, veterans, members of participating church. KQ3: participants from Community-based setting, academic and community internal medicine practices. | KQ1: From United States and Hong Kong. KQ2: from United States, Canada, England, Germany, Australia. KQ3: from United States, Canada, and Switzerland. | RTI International–University of North Carolina Evidence-based Practice Center (EPC) under contract to the Agency for Healthcare Research and Quality (AHRQ), Rockville, MD |
| Moorhead 2013 | Uses, benefits, and limitations of social media for health communication among the general public, patients, and health professionals. |  | 98 studies, 40 quantitative, 48 qualitative (including studies with content analysis presenting data with descriptive statistics), and 10 mixed methods. Quality was evaluated with the Downs and Black instrument, the maximum total score that could be achieved was 32, but the scores of the studies in this review ranged from 3 to 26. Overall, the studies scored low using this scale as they were mainly exploratory and descriptive with three intervention studies and one RCT. | February 2012 | Social media for health communication | The characteristics of users of social media were diverse, covering a range of different population groups. The age of the social media users ranged from school children to older adults aged 65 years and up, but the majority of the reported ages were 11-34 years. Some studies reported that there were more female than male users of social network sites. A few studies found that social media users were disproportionately from lower-income households. Studies within the United States reported that more social media users were African Americans than no Hispanic Whites. It is difficult to have the exact number of participants and their settings. | It was difficult to have the exact number of participants and their settings. Many studies reported participants but in other cases they reported number of Tweets, questions, comments, etc. Social media tools/applications (considered as "settings") within the 98 studies (Some studies included more than one social media tool/application).There was a wide range of health topics, but the most frequently reported on were sexual health, diabetes, flu/H1N1, and mental health issues such as stress or depression. Facebook (n=13), Blogs (n=13), Twitter (n=8), YouTube (n=7), MySpace (n=5), Wikipedia (n=3) , Wiki (n=2), Quitnet / online smoking cessation support group (n=2), Physician rating website specified) (n=2), Second Life (n=1), Daily Strength (n=1) , ArboAntwoord (n=1) , Social media (tool not specified) (n=30), Web 2.0 application (not specified) (n=11) | Scarcely reported. It is difficult when the object of study are the social media | Not reported |
| Pires 2015 | To review the readability of package leaflets of medicinal products for human use. |  | Twenty-two studies and comprised 16 full papers, three brief communications, and three indexed abstracts. The studies were distributed as follows: two exploratory studies enrolling health professionals, 12 exploratory studies enrolling patients or potential patients who will use the medicines, and 8 descriptive studies. | February 2013 | Package leaflets organized in clear and comprehensive way about medicines, adverse effects, information in different formats | Exploratory studies with the participation of health professionals (2 studies): one study included potential users and physicians and the other study included 40 patients, 6 physicians, 11 pharmacists and 13 from associations of patients and health professionals. Exploratory studies with the participation of potential users of medicines studies on patients’ comprehension of drug adverse reactions two studies with a total of 321 participants.  Exploratory studies with the participation of users or potential users of medicines comprehension (10 studies): With a total of approximately 4000 participants including: healthy men and with health problems, potential users, clients of community pharmacies and low-literate participants. | Descriptions were insufficient. In some cases, were clients of community pharmacies in others, were from associations of patients. Exploratory studies enrolling health professionals, patients or potential users of medicines: the majority of the reviewed readability studies used package leaflets of specific medicines, and highly utilized medicines such as acetaminophen. | Not described | Research supported by the Fundação para a Ciência e Tecnologia, Ministério da Educação e Ciência, Portugal (Process SFRH /BD/76531/2011 – Doctoral grant). |
| Revere 2001 | Effectiveness of patient-interactive computer-generated health behavior interventions— clinical encounters “in absentia”—as extensions of face-to-face patient care. This review specifically examines the state of computer-generated or computer-operated therapeutic communications | Yes | 37 studies: 3 Quasi-experimental, 28 RCT, 1 Feasibility study, 1 report, 4 other trials. | 1999 | patient-interactive computer-generated or computer operated health behavior interventions—clinical encounters “in absentia”—as extensions of face-to-face patient care | Mobile Communications: 50; Computer Systems: 961, Automated Telephone Communications: 1850 and Print Communications: 43868 | Not reported | Not reported | Not reported |
| Ryan 2014 | To assess the effects of interventions which target healthcare consumers to promote safe and effective medicines use, by synthesizing review-level evidence | Yes | Overview of 28 Cochrane reviews and 47 DARE reviews. 32/75 reviews (43%) included only randomized controlled trials (RCTs), other studies: quasi-randomized controlled trials (CCTs), controlled before-and-after studies (CBAs), interrupted time series (ITS) or before-and-after (BA) studies | March 2012 | Interventions to promotes safe and effective medicines use. Eight categories defined the interventions according the developed taxonomy (but 2 specific not were considered):  1. Providing information or education 2.Facilitating communication and/or decision making 3.Acquiring skills and competencies 4.Supporting behavior change 5. Support 6.Consumer system participation. **Interventions:** 1) Written information, fact sheets, booklets, newsletters, educational videos, support, counselling 2) Written action plans, on-to-one consultation, written question lists for providers; provider- patient communication skills training 3) Skills training, self-management  4) provider-led services for patients, reminders, appointment cards, medicines charts, alarms, memory aids, patient reminder or recall systems (such as letters, postcards, telephone follow-up, reminders with or without outreach) 5) Counselling (group or individual, structured) and support , therapy (family intervention, psychological therapy, cognitive behavioral therapy, motivational interviewing, group programs (peer support and shared identification)  6) Consumer involvement in developing patient medicines information, medicines policy or guideline committee involvement | Participants were consumers and providers. Most studies involved adult participants. Seven reviews focused on older adults (60 years or older), while another nine reviews incorporated a wide range of ages that explicitly included older with younger adults, and one review included children alone. Most studies focused on people with a condition and/or taking medicines, as opposed to careers, 19 reviews included studies with careers. Healthcare professionals were included as recipients of the intervention alongside consumers in 22 reviews. Twenty-one reviews included children together with older participants. | The reviews included cross-disease populations, and there was a large spread of acute and chronic conditions represented, as well as interventions specifically addressing immunization uptake, contraceptive use, post-surgical pain relief and medicines for infectious diseases. 46 reviews evaluated interventions for medicines use in relation to a particular medical condition. | No mentioned | Cochrane Infrastructure Grant provided by the National Health and Medical Research Council (NHMRC). Different government agencies at Australia and Canada paid researcher salaries |
| Sawesi 2016 | To determine (1) the impact of information technology (IT) platforms used to promote patient engagement and to effect change in health behaviors and health outcomes, (2) behavioral theories or models applied as bases for developing these interventions and their impact on health outcomes, (3) different ways of measuring health outcomes, (4) usability, feasibility, and acceptability of these technologies among patients, and (5) challenges and research directions for implementing IT platforms to meaningfully impact patient engagement and health outcomes. | Yes | 170 articles included, dominated by RCT (65.9%, 112/170). Case study (4 %), Cohort study (11,2%), Cross-sectional (8,8%), Quasi-experimental (10%) | Publication years ranged from 2000 to 2014, with an overall increase in articles published more recently (21.8%, 37/170 in 2014). | Different categories of IT platforms (including Internet-based interventions, mobile-based interventions, social media , video game technology , and telemonitoring). 1 week to 48 months | Sample sizes ranged from 1-22,337 subjects. | Not reported | The majority of studies were implemented in high income countries corresponding to United States the 54.7% (93/170). Just 1 study from Kenya and 1 from Chile. | Not reported |
| Sawmynaden 2012 | To assess the effects of email for the provision of information on disease prevention and health promotion, on outcomes for healthcare professionals, patients and caregivers, and health services, including harms. |  | Nine articles reporting six studies. Six RCT were included in qualitative synthesis, and 2 of them included in meta-analysis | 2010 | e-mail vs standard mail or standard care. Encrypted or Web messaging. For prevention, promotion or information. Weekly to six months | 8372 participants. All participants were adults, in five studies they were patients and in one, they were caregivers.  All participants needed to have e-mail access. | Primary care (1); secondary or tertiary care studies - hospital-owned health and fitness facility (1); outpatient clinics (4). | USA (5); UK (1). | Internal sources: eHealth Unit, Imperial College, UK, the NIHR (CLAHRC) Scheme, Department of Family Medicine, University of Ljubljana, Slovenia, NHS Education for Scotland, UK, NHS Connecting for Health Evaluation Programme (NHS CFHEP 001). External sources: Medical Research Council, UK. |
| Sharma 2017 | The impact of interventions involving patient advisory councils on clinical care outcomes, patient safety, and patient satisfaction, compared to care that doesn’t involve patient advisors. The secondary aim was to survey the impact patient advisors have on healthcare changes such as priority-setting, patient materials, and impacts on patient advisors themselves. |  | N=32, including: 1 cRCT, 4 describe a set of quasi-experimental quality improvement initiatives from one practice group, 1 systematic review, 1 cross-sectional survey, 9 qualitative or ethnographic studies, and 16 case studies | August 2015 | Patient advisory council (consumer working with healthcare staff): meetings 2 to 4 times per year. Face-to-face meetings, virtual meetings, focal groups, e-mail, communications) i.e.: Patients advisory, Community advisory council, AD-Hoc Committee, experience-based, co-design | Mostly patients (and families), including in: Patient Advisory Council (11 studies), Community Advisory Council (4), Ad-hoc Patient Committee (8), Experience-based co-design (4), Other (5). | Inclusion criteria spanned all healthcare settings, including primary care, ambulatory specialty care, inpatient care, emergency department and long-term care. Patient engagement at the clinic or organizational level was included. | 15 UK, 8 USA, 4 Canada, 4 Australia/NZ, and 1 Sweden | NRSA Award T32HP19025 & Kaiser Permanente National Community Benefit Fund of the East Bay Community Foundation #20152632. |
| Shipper 2016 | To assess what dissemination strategies are feasible to inform and educate patients about recommendations or guidelines. |  | 22 studies: 12 expert opinion 4 qualitative research 1 RCT | February 2016 | Guideline dissemination consists of a combination of active and passive methods: Using Websites, email notices, interactive internet-based lectures, telephone-based coaching, personal stories of patients in media, electronic point-of-care tools, templates, laboratory prompts and a communications campaign, interactive decision support algorithms. Training and support with learning tools (newsletters, brochures, posters, summaries, handouts, pocket cards, standardized slide sets), support groups, workshops, events, seminars, annual conferences, local or regional events, events for professionals and/or patients, press releases, print-ready ads, flow sheets didactic educational meetings, availability of cross-cultural adaptations, providing lay versions. Also, the establishment of permanent groups, networks or ‘virtual panels’ of patients to disseminate guidelines. The use of knowledge brokers (KB) as other strategy. | Not described | Different settings, diseases mainly about the field of rheumatology, asthma/COPD and diabetes and, participants including in the developing clinical practice guidelines. | Canada 7 Europe 7 USA 5 Russia 1 Africa 1 | EULAR European League Against Rheumatism funds |
| Smaihodzic 2016 | The effects of social media use for health related reasons on patients and their relationship with healthcare professionals | Yes | 22 Articles, nine quantitative, seven qualitative and six mixed methods studies. No experimental studies. | March 2015 | Social media | Patients users of social media. 4509 participants (adult and children) and 5400 posts (4 studies). | Social media platform and Condition: Blogs - Unknown (1), Blogs - Chronic(1), Facebook/Twitter - Obesity (1), Facebook - Mental (2), Facebook - Contraceptives (1), YouTube - Chronic (1), Online support community - Unknown (2), Online support community - Mental (1), Online support community - Chronic (10), Forum - Unknown (1), Forum - Mental (1), Virtual reality - Chronic (1) | 1556 participants were from 5 countries: Australia, Germany, Japan, Korean, USA, Taiwan. The remained 2953 came from online communities, networks, support groups and social media. | None |
| Stacey 2012 | To explore characteristics and effectiveness of decision coaching evaluated within trials of patient decision aids (PtDAs) for health decisions. |  | 10 RCT | 2015 | Decision coaching, some interventions included PtDAs. Median of individual coaching: 45 min, group coaching median 90 min for 3 sessions | 2625 participants of (women and men).The mean number of participants per trial was 242 (range 43–625). | Hospital, inpatients, Primary care, Specialty care, community | US, Canada, UK, Germany | Authors received funding for  research from the not-for-profit Foundation for Informed Medical Decision  Making (FIMDM). |
| Stacey 2017 | To assess the effects of decision aids (PtDA) in people facing treatment or screening decisions.  (This update compared only decision aids with usual care, being removed the comparation between single or multiple decision aids.) |  | 105 RCT involving 31,043 participants (this update added 18 studies and removed 28 previously included studies comparing detailed versus simple decision aids). | 2015 | PtDAs with: information, probabilities, steps for decision-making, clarify values, examples of other´s experiences. Included booklet, pamphlet, counselling, brochures, leaflet and electronic resources | Adults aged 18 years or older who made decisions about screening or treatment options. | The PtDA were set for screening or treatment of health conditions. | Australia (10 studies) Canada (15 studies) China (1 study) Finland (2 studies) Germany (6 studies) Netherlands (2studies) Spain (1 study) Sweden (1 study) UK (16 studies) USA (50 studies) Australia plus Canada (1 study) | The authors had financial support from the not-for-profit Informed Medical  Decisions Foundation (IMDF) |
| Sustersic 2016 | Summarize the diverse reviews, both general and specific to given conditions that use PILs (literature reviews, systematic reviews and meta-analyses), made to date. Clarify the impact of PILs by evaluating their effect on main outcomes, and specify their prescription according to condition and terms of use. Propose a checklist for writing, designing and using PILs with recommendations, for the standardization of research protocols that assess PILs. | Yes | This is an overview with 24 studies: 17 systematic reviews; 6 literature reviews; 1 meta-analysis. | August 2015 | PILs before consultation, screening or surgery, medication information. Web-pages, compute-linked, audiotapes, interactive videos, AIDs, counselling, teaching interventions. | Patients undergoing treatment for general conditions, cancers, acute diseases, chronic diseases and before an intervention. | The principle targets of PILs were drug treatments, invasive procedures (such as surgery or colposcopy, screening and cancer). Very few articles concern acute pathologies or general medicine. | Not informed | None |
| Vernooij 2016 | To characterize effective types of self-management interventions that could be packaged as resources in (i.e., appendices) or with guidelines (i.e., accompanying products). The secondary purpose of this review was to assess whether single-component self-management interventions were as effective as multifaceted self-management interventions. |  | Seventy-seven studies were included | February 2015 | Packaged resources or CPG: educational sessions, self-directed guides, or counseling (brief in-person or virtual interaction during which providers and/or lay leaders provided patients with recommendations, reminders or encouragement). | Not described | Eligible reviews addressed a wide range of clinical topics that were categorized as metabolic conditions which were all related to diabetes (23, 29.9 %); musculoskeletal conditions such as arthritis and back pain (12, 15.6 %); reviews of a variety of chronic conditions (12, 15.6 %); cardiovascular conditions such as angina, hypertension, heart disease, and stroke (11, 14.3 %); pulmonary conditions such as asthma and chronic obstructive pulmonary disease (7, 9.1 %); other conditions such as cancer pain, irritable bowel syndrome, epilepsy, multiple sclerosis, and kidney disease (7, 9.1 %); and mental illness including anxiety and depression (5, 6.5 %). | China(1)Netherlands(1)Not reported(75)United States(1) | None |
| Vodopivec 2012 | To assess the effects of mobile phone messaging interventions as a mode of delivery for preventive health care on health status and health behavior outcomes. |  | RCT 4 | June 2009 | SMS and Multimedia Messaging Service (MMS) for preventive and health behavior. Daily to every three days; six to 12 weeks of follow-up | Pregnant women (2), Children (1), Mixture of Smokers (1). The use of SMS messaging was applied to four different clinical areas: adherence to preventive medication, prenatal support, smoking cessation, and health behaviors. Participants in three studies were people in the community and in one study healthy pregnant women attending an ambulatory antenatal clinic. The target group for the intervention varied. The age and gender of participants were variable. One study included only women. Participants came from different income levels and ethnicities. | guidelines---- University students, healthy pregnant in antenatal care, smokers from community and children from community. | Canada, Thailand, New Zealand, USA | Ministry of Higher Education, Science and Technology, Slovenia. |
| Wantland 2004 | To provide further information on patient/client knowledge and behavioral change outcomes after Web-based interventions as compared to outcomes seen after implementation of non-Web-based interventions. | Yes | 22 studies (16 Randomized study - 3 convenience sample - 1 longitudinal randomized case control pre-post study - 1 descriptive study - 1 longitudinal study) | December 2003 | Web-based. 2.6 logons per person per week. Three to 78 weeks of follow-up. One-time Web-participant health outcome studies compared to non-Web participant health outcomes, self-paced interventions | Aggregation of data from the 22 selected studies showed a total of 11,754 participants in both the Web-based and non-Web-based interventions. Of this total, 5,841 were women and 5,729 were men. The average age of participants was 41.5 years. | Clinic and clinic/home based studies across many clinical areas or disorders of interest. | The selected studies were performed in the United States, France, Japan, Italy, Spain, Netherlands, Sweden, and Germany. | Not described |
| Wilson 2012 | Evaluate the evidence regarding the relative effectiveness of multimedia and print as modes of dissemination for patient education materials; examine whether development of these materials addressed health literacy. |  | 30 RCT | November 2010 | Multimedia and print as modes of dissemination for patient education materials. Electronic sources and oral communication, prints, booklets | Patients from diverse context and with many conditions. Sample sizes not reported. | Settings were diverse, ranging from primary care to specialty practice and from home to community-based care. | Not reported | This comparative analysis of print and multimedia health materials were funded, in part, by a grant from the Foundation for Informed Medical Decision Making (PI: Dr. Makoul). |
| Yamada 2015 | Evaluate the effectiveness of toolkits as a knowledge translation (KT) strategy for facilitating the implementation of evidence into clinical care. Toolkits include multiple resources for educating and/or facilitating behaviour change. |  | All study designs were included. Total 39 (11 RCTs, 13 cohort studies, 15 others. We just included the description of the 2 studies (RCTs) addressing toolkits to patients/caregivers | November 2013 | Toolkits: self-test, sheets, books, CDs, Audio CDs, booklets, mail | Participant of the two studies described, with focus on recipient health care. Patients with arthritic conditions: 921 adults with osteoarthritis, rheumatoid arthritis, fibromyalgia or chronic joint symptoms Caregivers of patients with Alzheimer's: 108 dyads of patients with progressive dementia of Alzheimer’s type/caregiver. | Among all of the toolkits, 20 were developed for a specific disease context, most commonly for cancer (n=8) and diabetes (n=3). The remaining toolkits were developed for disease prevention (n=5), infection prevention (n=2), postoperative pain (n=1), smoking cessation (n=1), care in the geriatric population (n=8), patient safety (n=1) and general hospital quality improvement (n=1). Two studies targeted to patients/caregivers: a) patients with Alzheimer and their caregivers were enrolled from geriatric specialty clinics, b) the arthritic patients receipt the toolkit by mail. | USA (2) | None |
| Zhao 2016 | To examine the effectiveness of mobile phone apps in achieving health-related behavior change in a broader range of interventions. | Yes | 23 studies used RCT design, except one case-control study. | June 2015 | Mobile-phone based app, video, SMS, Computer Groups, bibliotherapy. 19 days to 12 months, follow up between 3 weeks to 6 months | Adult population | Health care settings without specification. | All studies were conducted in high-income countries, 10 in the United States, 3 in Australia, 2 in the United Kingdom and Sweden, respectively, and 1 each in South Korea, Italy, New Zealand, Spain, Switzerland, and the Netherlands. | Not reported |

* For more details about interventions see Tables 2 and 3 in Additional file 5

### Table 2. Quality of included systematic reviews (AMSTAR)

| **Reference** | **Q1** | **Q2** | **Q3** | **Q4** | **Q5** | **Q6** | **Q7** | **Q8** | **Q9** | **Q10** | **Q11** | **Total of**  **'Yes' scores** |
| --- | --- | --- | --- | --- | --- | --- | --- | --- | --- | --- | --- | --- |
| Abu Abed 2014 | No | Yes | Yes | No | No | Yes | Yes | No | Yes | No | No | 5/9 |
| Akesson 2006 | No | No | Yes | No | No | Yes | Yes | No | NA | No | No | 4/10 |
| Akl 2011 a | Yes | Yes | Yes | No | Yes | Yes | Yes | Yes | Yes | Yes | No | 9/11 |
| Akl 2011 b | Yes | Yes | Yes | Yes | Yes | Yes | Yes | Yes | Yes | Yes | No | 10/11 |
| Ammentorp 2013 | No | Yes | Yes | Yes | No | Yes | Yes | Yes | NA | No | No | 6/10 |
| Ammenwerth 2012 | No | Yes | Yes | Yes | Yes | Yes | Yes | No | NA | No | No | 6/10 |
| Atherton 2010 | Yes | Yes | Yes | Yes | Yes | Yes | Yes | Yes | Yes | Yes | No | 10/11 |
| Bekker 2013 | No | Yes | Yes | Yes | No | Yes | No | No | Yes | No | No | 5/11 |
| Berkman 2011 | No | Yes | Yes | Yes | Yes | Yes | Yes | Yes | NA | No | No | 7/10 |
| Büchter 2014 | No | Yes | Yes | Yes | No | Yes | Yes | Yes | Yes | No | No | 7/11 |
| Car 2011 | Yes | Yes | Yes | Yes | Yes | Yes | Yes | Yes | NA | NA | No | 8/9 |
| Cole-Lewis 2010 | Yes | Yes | Yes | No | No | Yes | Yes | Yes | NA | NA | No | 6/9 |
| Edwards 2000 | No | Yes | Yes | Yes | No | No | Yes | Yes | Yes | Yes | No | 7/11 |
| Faber 2009 | No | Yes | Yes | No | No | Yes | Yes | Yes | NA | NA | No | 5/9 |
| Finkelstein 2012 | Yes | Yes | Yes | No | Yes | Yes | Yes | Yes | NA | No | Yes | 8/10 |
| Fjeldsoe 2009 | No | Yes | Yes | No | Yes | Yes | No | No | NA | No | No | 4/10 |
| Gagliardi 2016 | Yes | Yes | Yes | No | No | Yes | Yes | Yes | NA | No | No | 6/10 |
| Gibbons 2009 | Yes | Yes | Yes | Yes | Yes | Yes | Yes | Yes | NA | NA | No | 8/9 |
| Health Quality Ontario 2013 | No | No | Yes | Yes | No | Yes | Yes | Yes | Yes | NA | No | 6/10 |
| Hoffman 2017 | Yes | Yes | Yes | No | No | Yes | Yes | Yes | Yes | No | No | 7/11 |
| Ketelaar 2011 | Yes | Yes | Yes | Yes | Yes | Yes | Yes | No | NA | NA | Yes | 7/9 |
| Kinnersley 2007 | Yes | Yes | Yes | Yes | Yes | Yes | Yes | Yes | Yes | Yes | No | 10/11 |
| Laranjo 2014 | Yes | Yes | Yes | Yes | Yes | Yes | Yes | Yes | Yes | Yes | No | 10/11 |
| Loudon 2014 | No | Yes | Yes | Yes | No | Yes | Yes | Yes | NA | NA | No | 6/9 |
| Maher 2014 | No | Yes | Yes | Yes | No | Yes | Yes | Yes | Yes | Yes | No | 8/11 |
| Mc Cormack 2010 | Yes | Yes | Yes | No | Yes | Yes | Yes | Yes | NA | No | Yes | 8/10 |
| Moorhead 2013 | No | Yes | Yes | No | Yes | Yes | Yes | Yes | NA | No | No | 6/9 |
| Pires 2015 | No | No | Yes | No | No | Yes | No | Yes | Yes | No | No | 4/11 |
| Revere 2001 | No | No | Yes | Yes | No | Yes | Yes | No | No | No | No | 4/11 |
| Ryan 2014 | Yes | Yes | No | No | Yes | Yes | Yes | Yes | Yes | NA | Yes | 8/10 |
| Sawesi 2016 | No | Yes | No | No | No | Yes | Yes | Yes | Yes | No | No | 5/11 |
| Sawmynaden 2012 | Yes | Yes | Yes | Yes | Yes | Yes | Yes | Yes | Yes | Yes | No | 10/11 |
| Sharma 2017 | Yes | Yes | Yes | Yes | No | Yes | Yes | Yes | Yes | No | No | 8/11 |
| Shipper 2016 | Yes | Yes | Yes | Yes | No | Yes | Yes | Yes | Yes | Yes | Yes | 10/11 |
| Smaihodzic 2016 | No | Yes | Yes | No | No | Yes | Yes | Yes | Yes | No | No | 6/11 |
| Stacey 2012 | Yes | Yes | Yes | Yes | Yes | Yes | Yes | Yes | Yes | Yes | No | 10/11 |
| Stacey 2017 | Yes | Yes | Yes | Yes | Yes | Yes | Yes | Yes | Yes | Yes | No | 10/11 |
| Sustersic 2016 | Yes | Yes | Yes | No | Yes | Yes | Yes | Yes | Yes | No | Yes | 9/11 |
| Vernooij 2016 | Yes | Yes | Yes | No | No | Yes | Yes | Yes | NA | No | Yes | 6/10 |
| Vodopivec 2012 | Yes | Yes | Yes | Yes | Yes | Yes | Yes | Yes | Yes | Yes | No | 10/11 |
| Wantland 2004 | No | No | Yes | Yes | No | Yes | Yes | Yes | Yes | No | No | 6/11 |
| Wilson 2012 | No | Yes | Yes | Yes | No | Yes | No | NA | No | No | No | 4/10 |
| Yamada 2015 | No | Yes | Yes | Yes | No | Yes | Yes | Yes | NA | No | No | 6/11 |
| Zhao 2016 | No | Yes | Yes | Yes | Yes | Yes | Yes | Yes | NA | No | Yes | 8/10 |

AMSTAR Questions: Q1) Was an ‘a priori’ design provided?, Q2) Was there duplicate study selection and data extraction?, Q3) Was a comprehensive literature search performed?, Q4)Was the status of publication (i.e. grey literature) used as an inclusion criterion?, Q5) Was a list of studies (included and excluded) provided?, Q6) Were the characteristics of the included studies provided?, Q7) Was the scientific quality of the included studies assessed and documented?, Q8) Was the scientific quality of the included studies used appropriately in formulating conclusions?, Q9) Were the methods used to combine the findings of studies appropriate?, Q10) Was the likelihood of publication bias assessed?, Q11) Was the conflict of interest stated?.

1. Akl EA, Oxman AD, Herrin J, Vist GE, Terrenato I, Sperati F, et al. Framing of health information messages. Cochrane Database of Systematic Reviews. 2011(12):CD006777. [↑](#footnote-ref-1)
2. Akl EA, Oxman AD, Herrin J, Vist GE, Terrenato I, Sperati F, et al. Using alternative statistical formats for presenting risks and risk reductions. Cochrane Database of Systematic Reviews. 2011(3):1-90. [↑](#footnote-ref-2)
