# Supplementary material for: Knowledge translation strategies for dissemination with a focus on healthcare recipients: an overview of systematic reviews
Source: Implement Sci. 2020 Mar 4;15:14. doi: 10.1186/s13012-020-0974-3 (PMC7057470; doi:10.1186/s13012-020-0974-3)
Supplement: Supplementary file 5 — Additional file 5. Strategies and types of communication or dissemination. Table 1 Strategies/Interventions (adapted from Lavis et al. 2015 [13]). Table 2 Types of communication or dissemination. Table 3 Characteristics of interventions (details). [file 13012_2020_974_MOESM5_ESM.docx]

## Additional file 5. Strategies and types of communication or dissemination

### Table 1. Strategies/Interventions (adapted from Lavis et al. 2015^[[1]](#footnote-1)^)

| **Author** | **AMSTAR** | **Providing Information or education** | **Behavior change support** | **Acquiring skills and competencies** | **(personal) Support** | **Communication and decision-making facilitation** | **Consumer system participation** |
| --- | --- | --- | --- | --- | --- | --- | --- |
| Abu Abed 2014 | Medium | X | X |  | X |  |  |
| Akesson 2006 | Medium | X | X | X | X | X | X |
| Akl 2011 a | High | X | X |  |  |  |  |
| Akl 2011 b | High | X | X |  |  |  |  |
| Ammentorp 2013 | Medium |  | X | X | X |  |  |
| Ammenwerth 2012 | Medium | X | X | X | X | X | X |
| Atherton 2010 | High | X | X |  |  | X | X |
| Bekker 2013 | Medium | X | X | X | X | X | X |
| Berkman 2011 | Medium | X | X | X | X | X | X |
| Büchter 2014 | Medium | X |  |  |  |  |  |
| Car 2011 | High | X | X | X | X |  | X |
| Cole-Lewis 2010 | Medium |  | X | X | X |  |  |
| Edwards 2000 | Medium | X | X | X | X | X |  |
| Faber 2009 | Medium | X |  |  |  |  |  |
| Finkelstein 2012 | High | X | X | X | X | X | X |
| Fjeldsoe 2009 | Medium | X | X | X | X | X | X |
| Gagliardi 2016 | Medium | X | X | X | X | X | X |
| Gibbons 2009 | High | X | X | X | X | X | X |
| Health Quality Ontario 2013 | Medium | X | X |  |  |  | X |
| Hoffman 2017 | Medium | X |  |  |  |  |  |
| Ketelaar 2011 | Medium | X |  |  |  |  | X |
| Kinnersley 2007 | High | X | X |  |  |  |  |
| Laranjo 2014 | High | X | X |  | X |  |  |
| Loudon 2014 | Medium | X | X | X | X | X | X |
| Maher 2014 | High | X | X | X | X | X |  |
| Mc Cormack 2010 | High | X | X | X | X | X | X |
| Moorhead 2013 | Medium | X | X | X | X | X | X |
| Pires 2015 | Medium | X | X |  |  |  |  |
| Revere 2001 | Medium | X | X | X | X | X | X |
| Ryan 2014 | High | X | X | X | X | X | X |
| Sawesi 2016 | Medium | X | X | X | X | X | X |
| Sawmynaden 2012 | High | X | X | X | X | X |  |
| Sharma 2017 | High |  | X |  |  | X | X |
| Shipper 2016 | High | X |  |  |  |  | X |
| Smaihodzic 2016 | Medium | X | X | X | X | X | X |
| Stacey 2012 | High | X | X |  |  | X | X |
| Stacey 2017 | High | X | X |  | X | X | X |
| Sustersic 2016 | High | X | X |  | X | X | X |
| Vernooij 2016 | Medium | X | X | x |  |  | x |
| Vodopivec 2012 | High | X | x | X | X | X |  |
| Wantland 2004 | Medium | X | X | X | X | X | X |
| Wilson 2012 | Medium | X | X |  | X |  |  |
| Yamada 2015 | Medium | X | X | X | X | X | X |
| Zhao 2016 | High | X | X | X | X | X | X |

### Table 2. Types of communication or dissemination

| **Reference** | **Written** | **Verbal** | **Electronic** | **Internet -based** | **Type of electronic communication or dissemination** | **Type of no electronic communication or dissemination** | **Individual** | **Groups** |
| --- | --- | --- | --- | --- | --- | --- | --- | --- |
| Abu Abed 2014 |  |  | x |  | Video |  |  |  |
| Akesson 2006 |  | x | x | x | Computer, telecomunication, telemedicine, TV. |  | x | x |
| Akl 2011 a | x |  | x |  | Videos, multimedia | Booklet | x |  |
| Akl 2011 b | x |  | x | x | 2/35 studies used Internet | Paper, leaflet | x |  |
| Ammentorp 2013 |  | x | x | x | Telephone, Internet |  | x | x |
| Ammenwerth 2012 |  |  | x | x | Portal, e-mail |  | x | x |
| Atherton 2010 |  |  | x | x | e-mail, virtual groups |  | x |  |
| Bekker 2013 | x | x | x |  | Computer linked | PtDAs | x |  |
| Berkman 2011 | x | x | x | x | Video, computer, slide show | Alternative document design | x | x |
| Büchter 2014 | x | x |  |  |  | Leaflet | x |  |
| Car 2011 |  | x | x | x | Website |  |  | x |
| Cole-Lewis 2010 |  |  | x | x | SMS |  | x |  |
| Edwards 2000 | x | x | x |  | Video | Leaflet |  |  |
| Faber 2009 | x |  | x |  | Website | Report cards | x |  |
| Finkelstein 2012 | x | x | x | x | IT applications (clinical decision aids , IT-guided disease management , and  telemedicine or telemonitoring systems ) | E.g. written asthma  diary + instructions for  self-management | x | x |
| Fjeldsoe 2009 | x | x | x | x | SMS, e-mail, interactive Website, telemedicine | Brochures | x | x |
| Gagliardi 2016 | x | x | x | x | Video, Website, computer program, multimedia | Print material | x |  |
| Gibbons 2009 | x | x | x | x | Health risk assessment, decision aids, phones, laptops, CD-ROMs, PDA/smartphones, SMS, Chat groups, discussion | Written information / questionnaire | x | x |
| Health Quality Ontario 2013 |  |  | x |  | E-tools: ePHRs |  | x |  |
| Hoffman 2017 |  |  | x |  | Television |  | x | x |
| Ketelaar 2011 | x |  | x | x | Personal mailings, broadcasting media | Newspapers, leaflets | x | x |
| Kinnersley 2007 | x |  | x |  | Video, audiotape, computer program | Question checklists | x |  |
| Laranjo 2014 |  |  | x | x | Twitter, Facebook, Websites, e-mail |  | x | x |
| Loudon 2014 | x |  | x | x | Website | Written recommendations | x |  |
| Maher 2014 |  |  | x | x | Facebook, Twiter, You Tube, blogs, SMS, forum, podcasts, e-mail, virtual reality |  | x | x |
| Mc Cormack 2010 | x | x | x | x | Blogs, Facebook,Twitter, You Tube, SMS, e-mail, Forum | Non-numeric, numeric, visual, narrative, framed, or postal presentations | x | x |
| Moorhead 2013 |  |  | x | x | Blogs, Facebook, Twitter, You Tube, My Space, Patients Like Me, Wikipedia, Wiki, QuitNet, Second Life, etc. Video |  | x | x |
| Pires 2015 | x |  |  |  |  | Leaflets | x |  |
| Revere 2001 | x |  | x | x | Computer , telephone | Print materials | x |  |
| Ryan 2014 | x | x | x |  | Video, telephone follow-up, Mass mailings | Postcard, letters, reminders  booklets, newsletters, written action plans or question lists | x | x |
| Sawesi 2016 |  |  | x | x | Internet-based and mobile-based interventions, social media, video game technology , and telemonitoring |  | x | x |
| Sawmynaden 2012 | x |  | x | x | e-mail, Webpages | Standard mail | x |  |
| Sharma 2017 |  | x | x |  | e-mail, virtual groups |  |  | x |
| Shipper 2016 | x | x | x | x | Websites, e-mail, interactive internet-based lectures, telephone, electronic point-of-care tools, communications campaign, interactive decision support algorithms, press releases, networks or ‘virtual panels’ of patients | Newsletters, brochures, posters, summaries, handouts, pocket cards | x | x |
| Smaihodzic 2016 |  | x | x | x | Blogs, Forum, Virtual reality Facebook/Twitter, YouTube, Online support community |  | x | x |
| Stacey 2012 | x | x | x |  | Telephone | PtDAs paper-based | x | x |
| Stacey 2017 | x | x | x | x | Computer software, video, educational tools, interactive Web, DVD, online program | paper-based information, pamphlet, booklet | x |  |
| Sustersic 2016 | x | x | x | x | Web-pages, compute-linked, audiotapes, interactive videos, AIDs | Leaflets | x | x |
| Vernooij 2016 | x | x | x | x | Computer, Internet, or IT if more than one electronic delivery mechanism was used. | Print material | x |  |
| Vodopivec 2012 |  |  | x | x | SMS and MMS |  | x | x |
| Wantland 2004 |  | x | x | x | Web-based, multimedia, class room, internet support, help seeking strategies, interactive tools, home-based computer network, computer assisted clinic, Kiosk |  | x | x |
| Wilson 2012 | x | x | x | x | Video or DVD, computer, film, slides, html, audiotape only or multiple videos | Leaflets and booklets | x | x |
| Yamada 2015 | x |  | x |  | CDs, Audio CDs | Booklets, Written information | x |  |
| Zhao 2016 | x |  | x | x | Mobile phone APP, video, SMS | Written information | x | x |

### Table 3: Characteristics of interventions (details)

| **Author** | **Intervention/strategy** |
| --- | --- |
| Abu Abed 2014 | The videos were very different in length, content, and structure. Three different formats to present the information: didactic, practice, and narrative presentations. No information about the development (video-design) of the video in six articles. In other six used the video to optimize a single maneuver. In eight articles there were detailed information, with a mix of theoretical and empirical rationales. About the application of the video, more than half (12/20) described the application, but did not monitor the video use. |
| Akesson 2006 | Three strategies/interventions grouped as following:  (1) support and help: internet and telemedicine; telematics; audio conferencing; simple-to-use-telephone. (2) education and information: internet lessons; information delivered from medical office; telemedicine; email discussion group. (3) telecommunication instead of on-site visiting: home visiting by cable tv network; telecommunication system; computer-linked outpatient clinic. |
| Akl 2011 a | Interventions consisted of positively versus negatively framed messages (attribute framing) or gain-framed versus loss framed messages (goal framing). The two messages being compared should describe the same health information. Were excluded studies of risk choice framing. The 51 comparisons were: 13 related to attribute framing and 38 of goal framing. The messages used in these comparisons were about: • screening (n = 19), • prevention (n = 19), • treatment (n = 8), and • other (n = 5: 2 harm, 1 diagnosis, 1 public health, 1 on abortion); 1 study used 2 types of messages. They used videos, information, messages, pamphlet, brochure, reminder letters framed, information about condition, multimedia, booklet, articles, hypothetical situations. |
| Akl 2011 b | Interventions consisted of presentations of a risk (eg frequencies, percentages, and probabilities) or risk reduction (eg relative risk reduction (RRR), absolute risk reduction (ARR), number needed to treat (NNT)) of the same evidence about health. Were alternative formats for presenting risks focused on either diagnostic or screening tests. Were focused on four comparisons: a comparison of statistical presentations of a risk (eg frequencies versus percentages) and three comparisons of statistical presentation of risk reduction: RRR versus ARR, RRR versus NNT, and ARR versus NNT. The different studies covered a number of chronic diseases (mainly cancer, cardiovascular), genetic testing, and vaccination. 2/35 RCT used Internet. |
| Ammentorp 2013 | The coaching only included methods that were in accordance with the description of life coaching. It included studies in which the coaching was as follows: i) based on the agenda of the patient and reflecting the present wishes and needs. The dialogue was holistic, individualized, and non-programmatic. ii) conducted by professional coaches or healthcare professionals with special training in coaching; iii) conducted as face-to-face, telephone, or internet coaching or a combination of these methods; and i) individual or group sessions or a combination of both methods.  Two studies used telephone only coaching, and three used both face-to-face and telephone coaching, with one of these studies also using group coaching. The number of coaching sessions ranged from 6–14; the coaching sessions were conducted over a period of 3 to 12 months. In three of the studies, the coaching was conducted by health professionals trained in coaching without any certified coach training. The other two projects used professional certified coaches. |
| Ammenwerth 2012 | (1) patients undergoing IVF (in-vitro fertilization) treatment, were offered access to web-site with general information about infertility, IVF and the fertility clinic; own medical record with all available information concerning the patient’s IVF or ICSI treatment; tailored, context-sensitive clarification of clinical information; and communication options. Duration of intervention was unclear; (2) diabetes mellitus patients, who had at least one visit with their designated primary care provider in the prior year, and who had logged in at least once in PatientGateway, had access to medication module to review medications and edit inaccuracies; most recent results and current treatments (glucose, blood pressure, LDL-C, preventive care); therapy concerns and requests; answer short questions on therapy adherence and adverse effects; and generate a diabetes care plan based on patients’ responses to the questions, to be used at the next clinical visit. Control group had access to limited information on the PatientGateway. Duration of intervention: 12 months; and (3) patients with congestive heart failure were offered access to web-interface of SPPARO (“System Providing Patients Access to Records Online”) with possibility of online access to clinical notes, laboratory test results; patient information packet; and send messages to the clinic and receive messages. Duration of intervention: 12 months. Finally, (4) general patient that used KP Connect Online for longer than 13 months and that had used at least one feature were invited to access the portal offering parts of their individual health record; health summary with problem list, medications, allergies; health record with immunizations; secure provider messaging; administrative requests (update medical record, appointments etc.); visit-related inquiries such as after-visit summary, future appointments; and educational materials. Duration of intervention was 2 to 13 months. |
| Atherton 2010 | Five studies used some form of web-messaging. In the remaining four studies the type of email was not specified. Comparators: a) Email with usual care compared to usual care alone (standard methods of communication), b) Email compared to telephone for delivery of counselling, c) Communication protocol. |
| Bekker 2013 | Interventions were: a) evaluating the effects of a personal story component of a PtDA intervention on people’s healthcare decision making, b) involving individuals making real or hypothetical decisions; c) presenting personal stories in the first or third person. The personal story types were: first-person scripted narrative communications tailored to the characteristics of the decision makers; third person scripted narrative describing other patients’ experiences; documentary illustrating the illness state and types of care; and conversations illustrating the interaction between patients and others. The personal stories varied in their content, delivery, and length, and provided information about the narrator’s perception of making the decision and the health context that reinforced aspects of the PtDA information of importance to the narrator. Some stories included additional information regarding the disease, others included additional information on engaging with health professionals and services, and interactive exercises from the PtDA. |
| Berkman 2011 | To the Q2: 21 studies used one specific strategy to mitigate the effects of low health literacy and 21 used a mixture of strategies combined into one intervention. Studies that used one specific low-literacy strategy: two focused on alternative document design, three on alternative numerical presentation, eight on additive or alternative pictorial representations, four on alternative media, and seven on a combination of alternative readability and document design. Additionally, one intervention focused on the effects of physician notification about patients’ literacy status on health outcomes. Mixed interventions included a combination of the strategies noted above and other strategies to promote improvements in patient knowledge, self-efficacy, behavior, adherence, disease, quality of life, and health care services use. |
| Büchter 2014 | All studies tested short information leaflets on drugs for a particular condition, which only differed in whether the information on the frequency of the adverse effects of the drug were presented verbally or numerically. One study examined a combination of a verbal and numerical description. The interventions of the studies were very similar. The verbal descriptors were: very common, common, uncommon, rare and very rare. All outcomes were measured shortly after distribution of the information leaflets, and none of the studies had a follow-up. |
| Car 2011 | The interventions were broadly similar in both studies, consisting of ’adult education style’ discussion, instruction and practice in groups of six to ten participants. The RCT provided a higher intensity intervention, with eight 120-minute sessions delivered over four weeks; 9-month follow-up for all outcomes except ’health information evaluation skills’, which was only measured at baseline and the 3 month follow-up. The CBA study provided four 90-minute sessions over four weeks. The CBA intervention was focused on locating trustworthy online health information and evaluating the quality of web sites, while the RCT intervention had a broader focus, and included using online health information (e.g. by taking it to a health professional), gaining social support online, and strategies for getting personal access to the internet in the future, as well as locating and evaluating online health information. |
| Cole-Lewis 2010 | Intervention length ranged from 3 months to 12 months, and none had long-term follow-up beyond completion of the intervention. Frequency and intensity of text messaging varied greatly. Some studies allowed participants to dictate the frequency of messaging, other used tailored messages. Most studies had an interactive component that requested input via text messaging from the participant; only 2 were unidirectional. In all studies, text messaging was initiated by the researcher with the exception of 3 studies, where researchers communicated with participants only after the participant sent a text message. All disease prevention studies used automated messaging, and provided tailored messages except for 2. All disease management studies used messages written by a medical professional upon chart review except one that provided automated, tailored messages. In only one disease management study could a participant reply to physicians’ medical advice with questions. Text messaging was the only intervention component in 5 studies, whereas others included supplementary components such as e-mail and the Internet. Only one study provided an additional tool for patient self-monitoring; all disease management studies required an additional tool for patient self-monitoring. All but 3 of the disease management studies provided participants with new innovations as opposed to the standard of care (i.e., a new glucose monitoring tool vs. the traditional finger-stick blood testing for diabetes). Three studies provided phones to patients. |
| Edwards 2000 | One-to-one communication (not necessarily face-to-face) in the health care setting or address relevant health topics to the subjects. Written information, counselling, video. Definition of risk communication: seeking to alter knowledge, perceptions, attitudes, or behaviors relating to risk and the communication should include a stimulus to patients to weigh up the risks and benefits of a treatment choice or behavioral risk-reducing change. |
| Faber 2009 | Quality-of-care information was either actual (based on real performance) or hypothetical (arranged for study purposes only); the latter often was the case in laboratory settings. Health care providers, intermediary organizations, insurance companies, and health plans (HP) all could be responsible for dissemination of the information to the public. The information was classified as: Type A covered all standard assessed consumer or patient experiences, like CAHPS (Consumer Assessment of Health care Providers and Systems) ; Type B comprised clinical performance- based quality information, like the AHRQ Quality Indicators, and type C included expert- or peer-assessed measures, like certification, accreditation, or quality ratings given by colleagues. |
| Finkelstein 2012 | There were a wide range of interventions (delivered at system, provider, and patient levels). KQ1a: The studies most commonly employed the following health IT applications: clinical decision aids (34 studies), IT-guided disease management (17 studies), and telemedicine or telemonitoring systems (20 studies. KQ1b: IT applications were clinical decision aids (23 studies), IT-guided disease management (19 studies), and telemonitoring systems (18 studies). KQ1c:IT applications most commonly addressed in these studies were telemonitoring (18 studies), clinical decision aids (16 studies), and IT-guided self-management (16 studies). KQ1d: several types of health IT, including clinical decision aids, IT-guided disease management tools, and shared decision-making tools. KQ1e: The studies most frequently used clinical decision aids (6 studies), shared decision-making tools (7 studies), and telemedicine or telemonitoring systems (7 studies). KQ2: Several studies design (experimental and non-experimental, descriptive, qualitative, and others) about barriers or facilitators for utilization of health IT applications. KQ3: very few studies addressed the cost or sustainability of using health IT to promote PCC. |
| Fjeldsoe 2009 | Tailored SMS delivered by Mobile Telephone for Behavior Change (4 studies used SMS for preventive health behavior change and 10 used SMS to support ongoing clinical care behavior change)  The mode of intervention initiation varied among studies: initiated by a face-to-face meeting with a health professional, SMS to initiate the program and gain participant consent, or an interactive website.  The initiation of SMS dialogue was a) researcher-initiated technique or participant-initiated technique.  The frequency of SMS transmission reflected the expected frequency of the targeted behavior (e.g., smoking [5/day], physical activity [5/week]). |
| Gagliardi 2016 | PKT interventions delivered immediately before, during or upon conclusion of clinical encounters to individual patients. The type of PKT intervention for patients including print material in 10 studies (brochures 5, booklets 1, variety of print material 2, list of websites 2), electronic material in 10 studies (video 4, computer program 5, website 1) and counselling in 2 studies. Interventions were characterized by type of patient engagement (inform, activate, collaborate). They were offered before, during and after consultation in 4, 1 and 4 studies, respectively; as single or multifaceted interventions in 10 and 6 studies, respectively; and by clinicians, health educators, researchers or volunteers in 4, 3, 5 and 1 study, respectively. |
| Gibbons 2009 | Consumers Health Information (CHI) applications:  55% of studies evaluated interactive Website–based applications or Web-based tailored educational Web sites. Another 15% of studies evaluated computer-generated tailored feedback applications. Interactive computer programs and personal monitoring devices were evaluated in approximately 8% of studies each. Finally, health risk assessments, decision aids, cell phones, laptops, CD ROMs, personal digital assistants (PDA/smartphones), short message system texting (SMS/text), discussion/chat groups and computer-assisted imagery were evaluated in less than 5% of studies each. |
| Health Quality Ontario 2013 | Strategies were focused on communication between health service's staff, but in some cases included patients. Were based on Electronic Data Interchange Systems (1); Electronic Health Records (4); software to automatically generate personalized discharge summaries (1); clinical use of computer (1); information system (1); support system (1); email system (1); and diabetes electronic management system (1). Follow up varied from 6 months to 5 years. |
| Hoffman 2017 | Intervention: To involve an exposure to fictionalized United States (U.S.) medical television programming premiering in 1994 or later. Fictional television programming according to the Academy of Television Arts & Sciences Emmy awards was defined as a primetime television drama or comedy series. The most commonly assessed programs were ER (73%), Grey’s Anatomy (58%) and House M.D. (37%Only one study used a delayed follow-up assessment. |
| Ketelaar 2011 | Performance data about any aspect of the healthcare organizations or individuals, including process measures, healthcare outcomes, structure measures, consumer or patient experiences and/or expert or peer-assessed measures. The data presented might or might not provide comparisons with similar providers or quality standards and might or might not be adjusted for case mix. Performance data might be prepared and released by any organization, such as the government, insurers or consumer organizations. The release of performance data into the public domain were in written or electronic form, with varying degrees of accessibility, such as a report in a publicly accessible library or more active dissemination directly to consumers in newspapers, leaflets, personal mailings, broadcasting media, etc. |
| Kinnersley 2007 | The most common interventions were question checklists and patient coaching. Most interventions were delivered immediately before the consultations but in 6 were some time before the consultation. 26 studies had single interventions and 7 multiples. Of the single interventions, 20 had only one component (written materials or coaching or audiotape) and 6 had multiple components (coaching combined with written materials, computer programs or video). The 7 studies assessing multiple interventions included different combinations among written materials, coaching, brief advice or message on question asking and also, studies compared two different forms of written materials or two different forms of coaching. Comparisons were usual care or a dummy intervention. 5 studies were for clinicians. |
| Laranjo 2014 | Facebook was the most utilized SNS (7 studies), either isolated or as part of a more complex intervention with other components. Twitter was used in one study and health-specific SNSs in 4 studies. The SNS component was primarily used as a means of providing education and social support. Only one study used for data sharing, with the goal of promoting accountability and social competition. Intervention components other than the SNS were primarily used for educational and self-monitoring purposes and were most often web-based. Studies duration varied from 21 days to 18 months. Five studies mentioned a health behavior theory or model underlying the intervention. Retention rates were above 80% in 4 studies, and between 65% and 75% in 2 others. Four studies did not report. |
| Loudon 2014 | The main intervention was to present or communicate the information included in clinical practice guidelines to public or patients. Specifically, the 3 RCTs included also included interventions used to communicate guideline information like use of symbols and words to present information on the strength of recommendations, specific tools and behaviorally specific language. |
| Maher 2014 | Online intervention delivered either wholly or in part, using an online social network (OSN) to deliver a health behavior change intervention. The OSN intervention could be delivered using an existing OSN platform (e.g., intervention delivered via either a “generic” pre-existing social networking website such as Facebook or Twitter, or a health-specific pre-existing social networking website, such as FatSecret) or a purpose-built intervention website incorporating social networking capabilities. In the case of purpose-built websites, studies had to explicitly describe their website as using social networking to be included. Participation rates varied widely, ranging from 33% to 89%. Interventions ranged from 5 days to 6 months in duration. No studies reported follow-up of outcomes and maintenance of behavior change beyond the end of the intervention itself. The Social media platform were: Blogs(2), Facebook+Twiter (1), Facebook (2), Youtube (1), Online support community (3), Forum (2), Virtual reality (1) |
| Mc Cormack 2010 | KQ1 strategies: Tailoring the message, targeting the message, using narratives, framing the message and more than one of the above strategies. Key points for communication strategies were as follows: Framing (gain/loss) versus narratives (yes/no), Framing (gain/loss) versus targeting (yes/no), Targeting (yes/no) versus tailoring (yes/no) and Targeting (yes/no) and tailoring (yes/no) versus targeting only. Some trials compared two strategies directly with each other; others used a combination of strategies. KQ2 strategies: included dissemination strategies and outcomes for clinicians and patients: a) Improve reach of evidence: distributing evidence widely to many audiences and across many settings extends the numbers and types of recipients ( i.e.: Postal, electronica and digital media, social media, mass media, Interpersonal verbal group or individual outreach). b) Motivate recipients to use and apply evidence: Using a variety of authoritative experts or spokespersons to increasing interest in or acceptability of the evidence or related recommendations may promote enthusiasm or action on the part of clinicians or patients (e.g. Champions, opinion leaders (frequently has an endorsing or persuasive element).c) More than one of the above strategies: combining multiple dissemination strategies, including ways to increase reach, motivation, or ability, may be more effective than single strategies. KQ3 strategies: examined alternative ways to communicate the precision, directness, and net benefit of evidence, and overall strength of recommendations through: Non-numeric, numeric, visual, tailoring, targeting, narrative, framed presentations, and more than one of the above strategies. For the KQ3 the key points for conveying uncertainty were as follows: Communicating precision, directness, net benefit and strength of recommendations. |
| Moorhead 2013 | Social Media for Health Communication: Social media allows information to be presented in modes other than text and can bring health information to audiences with special needs; for example, videos can be used to supplement or replace text and can be useful when literacy is low. Sites such as PatientsLikeMe , MySpace, YouTube, Blog sites, Facebook, can be used to collect data on patient experiences and opinions such as physician’s performance. Social media have been used for health promotion and health education and for delivering a health intervention by providing social support/influence. Social media can reduce stigma about certain conditions such as epilepsy. |
| Pires 2015 | Package leaflets that must be organized in pre-defined sections and written in a clear and comprehensible way. |
| Revere 2001 | Focus in two specific areas: the health behavior models used in interventions and the devices used for patient education, counseling, and reminder systems aimed at improving patient health behaviors. Items were classified by intervention type, delivery device, and use of synchronous vs. asynchronous interaction. Three intervention types according to features accepted in the literature: personalized, targeted, and tailored. They grouped intervention delivery devices into categories adapted from Balas et al.: 1) mobile communication systems (use of a pager, mobile telephone, or other wireless system for delivery), 2) computerized communication systems (use of a computer, modem, touch-sensitive screen, or other interfacing equipment for delivery), 3) automated telephone communication (usually computer-generated messages using a regular telephone line and telephone), and 4) print communication (use of a letter, bulletin, fax transmission, newsletter, postcard, or manual delivery). |
| Ryan 2014 | Eight categories defined the interventions according the developed taxonomy: 1.Providing information or education 2.Facilitating communication and/or decision making 3.Acquiring skills and competencies 4.Supporting behavior change 5. Support 6.Minimising risks or harms 7.Improving quality 8.Consumer system participation. Strategies directly targeting consumers ranged from the very simple (such as reducing the frequency of dosing, changing the medicine formulation, sending a postcard reminder) to the very complex (different combinations of education, medicines reconciliation and/or review, counselling and self-monitoring, or self-management programs). |
| Sawesi 2016 | Different categories of IT platforms were identified including Internet-based interventions (50.6%, 86/170), mobile-based interventions (25.9%, 44/170), social media (9.4%, 16/170), video game technology (3.5%, 6/170), and telemonitoring (10.6%, 18/170). With respect to the different targeted disorders, hormonal disorders were most frequently targeted (22.4%, 38/170 studies, e.g., diabetes). The duration of these studies ranged from 1 week to 48 months. |
| Sawmynaden 2012 | E-mails to provide information on disease prevention (3); e-mails to provide health promotion information by delivering tips (2); e-mail to promote and educational website (1), compared to standard mail or usual care. Interventions that used email in any of the following three forms: 1. Unsecured standard email to/from a standard e-mail account. 2. Secure e-mail which is encrypted in transit and sent to/from a standard e-mail account with the appropriate encryption decoding software. 3. Web messaging, whereby the message is entered into a proforma which is sent to a specific email account, the address of which is not available to the sender. |
| Sharma 2017 | Patient advisory council activity or intervention, defined for the purpose of this study as a group of patients or consumers working with healthcare staff in order to provide input on healthcare services or delivery. Of the 32 studies included: the primary mode of patient advisor intervention was a patient advisory council, referring to a group of patients who met with staff on a regular basis to discuss healthcare improvement activities. Patient engagement was also described in studies as community advisory councils (4 from one institution), experience-based co-design projects, ad-hoc patient committees who met for a single project, and “other” activities such as “mental health user groups” composed of patients who advise healthcare trusts in the UK. |
| Shipper 2016 | Guideline dissemination consists of a combination of active and passive methods: Using Websites, e-mail notices, interactive internet-based lectures, telephone-based coaching, personal stories of patients in media, electronic point-of-care tools, templates, laboratory prompts and a communications campaign, interactive decision support algorithms. Training and support with learning tools (newsletters, brochures, posters, summaries, handouts, pocket cards, standardized slide sets), support groups, workshops, events, seminars, annual conferences, local or regional events, events for professionals and/or patients, press releases, print-ready ads, flow sheets didactic educational meetings, availability of cross-cultural adaptations, providing lay versions. Also the establishment of permanent groups, networks or ‘virtual panels’ of patients to disseminate guidelines. The use of knowledge brokers (KB) as other strategy. |
| Smaihodzic 2016 | Six categories of patients’ use of social media were identified: a) social support emotional, esteem, information, network supports; b) Other types of use (emotional expression and social comparison). |
| Stacey 2012 | Decision coaching compared to another intervention and/or usual care. Specific terms used to describe the decision coaching included counseling, consultation, preference-elicitation interview, planning talk, coached-care, empowerment, and decision education session. Decision coaching was provided to patients in preparation for their consultation with practitioners in specialty care (n = 7), primary care (n = 2), or the community (n = 1). The length of time for the coaching intervention reported in 7 trials was a median of 45 minutes (range, 20– 270 minutes). The trial reporting 270 minutes provided coaching during a series of 3 group sessions of 90 minutes each. The typical decision coaching intervention included providing information, clarifying values, and facilitating progress in decision making. 6 trials used a PtDA. |
| Stacey 2017 | Patient decision aids included: information on the clinical problem (90,5%); outcomes probabilities (89,5%); guidance on steps of decision making (65,7%); explicit method to clarify values (57,1%); examples of other's experience (41%). All the studies included information about options and outcomes and provided clarification of values. |
| Sustersic 2016 | Patient Information Leaflets compared to none information or other forms of information. Seven reviews looked at information on drugs (including one on contraceptives and one on drugs for psychiatric disorders), three reviews concerned PILs for cancer patients, three were on PILs intended to be given before a screening examination or surgery, three were about common acute conditions and three on chronic diseases. |
| Vernooij 2016 | Packaged resources or CPG: educational sessions (single or multiple provider and/or lay leader sessions during which information was conveyed to individuals or groups in-person or by virtual means), self-directed guides (print material, computer, Internet, or information technology if more than one electronic delivery mechanism was used), or counseling (brief in-person or virtual interaction during which providers and/or lay leaders provided patients with recommendations, reminders or encouragement). Fifty-four focused on single (38 educational, 16 self-directed) and 21 on multifaceted interventions. Taxonomy of self-management components: Inform Information that provides patients with knowledge about their condition and an understanding of how to manage it (i.e. Lifestyle advice). Activate Information or tools to prompt action for actively managing the condition and enhancing quality of life (i.e. Support for condition). Collaborate Information or mechanisms that lead to interaction and engagement (i.e. Communication with clinicians) |
| Vodopivec 2012 | Interventions using SMS or MMS as a mode of delivery for any type of preventive health care between care provider and participants. The mobile phone messaging interventions were delivered using different platforms. |
| Wantland 2004 | Web-based interventions to achieve the specified knowledge and/or behavior change for the studied outcome variables. Longitudinal studies ranged from 3 weeks to 78 weeks in duration. |
| Wilson 2012 | Subjects were randomly assigned to an intervention group in 28 of the 30 studies. Interventions: A variety of multimedia formats were represented, including video (such as tape or DVD), computer, film, and slides accompanied by audiotape. A number of studies tested other modalities as well, such as oral communication with the provider, html, audiotape only, or multiple videos. Print materials included leaflets and booklets of various length and content. |
| Yamada 2015 | Intervention of the two studies described with focus on recipient health care: Patients with arthritic conditions: Toolkits contents (available in English and Spanish): ‘self-test’ to self-tailor the toolkit; information sheets on arthritic-related health issues and on key process components of the Arthritis Self-Management Program (e.g., decision-making); Arthritis Help Book; audio relaxation and exercise CDs; audio CD of all material from information sheets.  Caregivers of patients with Alzheimer's: Toolkit contents: ‘Keep the Home Safe for a Person with Memory Loss’ booklet; low-cost sample items to reduce risky behaviors and accidents. |
| Zhao 2016 | 3 mechanisms were employed to promote behavior change: behavior change theories (BCTs) in 6 studies, and specific behavioral therapies. The most commonly used theory was the theory of planned behavior, followed by social cognitive theory. The top 3 most commonly used BCTs were self-monitoring (12 interventions), feedback provided on performance (8 interventions), and tailoring messages (8 interventions). Apps related to mental health or alcohol addiction were usually based on a specific behavioral therapy, such as motivational enhancement therapy, behavioral activation therapy, and cognitive behavior therapy. Retention rates were between 60-100%. |

1. Lavis JN, Wilson MG, Moat KA, Hammill AC, Boyko JA, Grimshaw JM, et al. Developing and refining the methods for a 'one-stop shop' for research evidence about health systems. Health Research Policy and Systems. 2015;13(1):10. [↑](#footnote-ref-1)
