# Supplementary material for: Knowledge translation strategies for dissemination with a focus on healthcare recipients: an overview of systematic reviews
Source: Implement Sci. 2020 Mar 4;15:14. doi: 10.1186/s13012-020-0974-3 (PMC7057470; doi:10.1186/s13012-020-0974-3)
Supplement: Supplementary file 6 — Additional file 6. Types of outcome measures. [file 13012_2020_974_MOESM6_ESM.docx]

## Additional file 6. Types of outcome measures

| **Reference** | Access | Adherence/Compliance | Attitude /Motivation | Awareness | Behavoir/Intention | Beliefs | Clinical results | Comunication with physician | Costs | Coverage | Decision -making | Empowerment/ Engagement | Knowledge /understanding | Less suffering/ Anxiety | Persuasion | Safety | Satisfaction | Skill and competence (Self efficacy) | Social support | Use of services | Health literacy | Perception | Quality of life | Use of information | Health status and wellbeing | Hospitalizations | Length of consultation | Participation in health | Adiction to media | Sustainability | Choice |
| --- | --- | --- | --- | --- | --- | --- | --- | --- | --- | --- | --- | --- | --- | --- | --- | --- | --- | --- | --- | --- | --- | --- | --- | --- | --- | --- | --- | --- | --- | --- | --- |
| Abu Abed 2014 |  |  |  |  | x |  |  |  |  |  |  |  |  |  |  |  |  |  |  |  | X |  |  |  |  |  |  |  |  |  |  |
| Akesson 2006 | x |  |  |  | x |  | x | x | x |  |  | x | x | x |  |  | x | x | x |  | x |  |  |  |  |  |  |  |  |  |  |
| Akl 2011a^[[1]](#footnote-1)^ |  |  |  |  | x |  |  | x |  |  |  |  | x |  |  |  |  |  |  |  |  | X |  |  |  |  |  |  |  |  |  |
| Akl 2011b^[[2]](#footnote-2)^ |  |  |  |  | x |  |  | x |  |  |  |  | x |  |  |  |  |  |  |  |  | x |  |  |  |  |  |  |  |  |  |
| Ammentorp 2013 |  |  |  |  |  |  |  |  |  |  |  | x |  |  |  |  |  | x |  |  |  |  |  |  |  |  |  |  |  |  |  |
| Ammenwerth 2012 | x |  |  |  | x |  |  | x |  |  |  | x | x |  |  |  |  |  | x | x |  |  |  |  |  |  |  |  |  |  |  |
| Atherton 2010 | x |  |  |  | x |  |  | x |  | x | x |  | x |  | x |  | x |  |  | x |  |  |  |  |  |  |  |  |  |  |  |
| Bekker 2013 |  | x | x |  | x |  |  | x |  |  | x | x | x | x | x | x | x | x |  | x |  |  |  |  |  |  |  |  |  |  | x |
| Berkman 2011 |  | x |  |  | x |  | x |  | x |  | x |  | x |  |  |  |  | x | x | x |  |  | x |  |  |  |  |  |  |  |  |
| Büchter 2014 |  | x |  |  | x |  |  |  |  |  |  |  |  |  |  |  | x |  |  |  |  |  |  |  |  |  |  |  |  |  |  |
| Car 2011 |  |  |  |  | x |  |  | x |  |  |  |  | x |  |  |  |  | x |  |  |  |  |  | x |  |  |  |  |  |  |  |
| Cole-Lewis 2010 | x |  |  |  | x |  | x | x |  |  | x |  | x |  |  |  |  | x |  | x |  |  |  |  |  |  |  |  |  |  |  |
| Edwards 2000 |  |  |  |  |  |  |  |  |  |  |  |  |  |  |  |  |  |  |  |  |  |  |  |  |  |  |  |  |  |  | x |
| Faber 2009 |  |  | x | x | x |  |  |  |  |  |  | x | x |  |  |  |  |  |  | x |  |  |  |  |  |  |  |  |  |  | x |
| Finkelstein 2012 | x | x | x |  | x |  | x | x | x | x | x | x | x |  |  | x | x | x |  | x |  |  |  |  |  |  |  |  |  |  |  |
| Fjeldsoe 2009 | x | x |  |  | x |  | x | x |  | x | x | x | x |  |  |  |  | x |  | x |  |  |  |  |  |  |  |  |  |  |  |
| Gagliardi 2016 |  |  | x |  | x |  |  | x |  |  | x | x | x |  |  |  | x | x |  |  |  |  |  |  |  |  |  |  |  |  |  |
| Gibbons 2009 | x | x | x |  | x | x | x | x | x | x | x | x | x |  |  | x | x | x | x | x |  |  | x |  |  |  |  |  |  |  |  |
| Health Quality Ontario 2013 |  |  |  |  |  |  |  |  |  |  |  |  |  |  |  |  |  |  |  | x |  |  |  |  |  |  |  |  |  |  |  |
| Hoffman 2017 |  |  |  |  | x |  |  | x |  |  |  |  | x |  |  |  |  |  |  |  |  | x |  |  |  |  |  |  |  |  |  |
| Ketelaar 2011 |  |  | x | x |  |  |  |  | x |  |  |  | x |  |  |  |  |  |  | x |  |  |  |  |  |  |  |  |  |  |  |
| Kinnersley 2007 |  |  | x |  |  |  |  | x |  |  |  |  | x | x |  |  | x |  |  | x |  |  |  |  |  |  | x |  |  |  |  |
| Laranjo 2014 |  | x |  |  | x |  |  |  |  |  | x | x | x |  |  |  |  | x | x |  |  |  |  |  |  |  |  |  |  |  |  |
| Loudon 2014 |  | x | x | x |  |  |  | x |  |  | x | x | x |  |  |  |  | x |  | x |  |  |  |  |  |  |  |  |  |  |  |
| Maher 2014 |  |  | x | x | x |  | x |  |  |  |  | x | x |  |  |  |  | x | x |  |  |  |  |  |  |  |  |  |  |  |  |
| Mc Cormack 2010 |  | x | x | x | x |  | x | x |  |  | x | x | x | x |  | x | x | x | x | x |  | x |  |  |  |  |  |  |  |  |  |
| Moorhead 2013 | x |  |  |  |  |  |  | x |  |  |  |  | x | x |  |  |  | x |  |  |  |  |  |  |  |  |  |  |  |  |  |
| Pires 2015 |  |  |  |  |  |  |  |  |  |  |  |  | x |  |  |  |  |  |  |  | x |  |  |  |  |  |  |  |  |  |  |
| Revere 2001 |  | x | x | x | x |  | x | x |  |  | x | x | x |  |  |  |  | x | x | x |  |  |  |  |  |  |  |  |  |  |  |
| Ryan 2014 |  | x | x |  | x |  | x | x |  |  | x | x | x | x |  | x | x | x |  | x |  |  |  |  |  |  |  |  |  |  |  |
| Sawesi 2016 |  | x | x | x | x | x | x | x |  |  | x | x | x | x |  |  | x | x | x | x |  |  | x |  |  | x |  |  |  |  |  |
| Sawmynaden 2012 |  | x |  |  | x |  | x | x |  |  | x |  |  |  |  | x |  |  | x |  |  |  |  |  | x |  |  |  |  |  |  |
| Sharma 2017 |  |  |  |  |  |  | x |  |  |  |  | x |  |  |  | x | x |  |  |  |  |  |  |  |  |  |  | x |  |  |  |
| Shipper 2016 |  |  |  |  |  |  |  | x |  |  |  | x | x |  |  |  |  |  | x |  |  |  |  |  |  |  |  |  |  |  |  |
| Smaihodzic 2016 |  | x | x |  | x |  |  | x |  |  | x | x | x |  |  |  |  | x | x |  |  |  |  |  |  | x |  |  | x |  |  |
| Stacey 2012 |  | x |  |  | x |  |  | x | x |  | x | x | x |  |  |  | x |  |  |  |  |  |  |  |  |  |  |  |  |  | x |
| Stacey 2017 |  | x |  |  | x |  |  | x | x |  | x | x | x | x |  |  | x | x |  |  |  |  |  |  |  |  | x |  |  |  |  |
| Sustersic 2016 |  | x | x |  | x |  |  | x |  |  | x | x | x | x |  | x | x | x | x | x |  |  |  |  |  |  |  |  |  |  |  |
| Vernooij 2016 |  | x |  |  | x |  |  | x |  |  | x | x | x |  |  |  |  |  | x |  |  |  |  |  |  |  |  |  |  |  |  |
| Vodopivec 2012 |  | x |  |  | x |  |  | x |  |  |  |  |  | x |  |  | x | x |  |  |  |  | x |  |  |  |  |  |  |  |  |
| Wantland 2004 |  | x |  | x | x |  |  |  |  |  |  |  | x |  |  |  | x | x | x | x |  |  |  |  |  |  |  | x |  |  |  |
| Wilson 2012 |  | x | x | x |  |  |  | x |  |  | x | x | x | x |  |  |  |  |  |  |  |  |  |  |  |  |  | x |  |  |  |
| Yamada 2015 |  |  |  |  | x |  | x |  | x |  | x | x | x |  |  |  |  | x |  | x |  |  |  |  |  |  |  |  |  | x |  |
| Zhao 2016 |  | x | x |  | x |  | x | x |  |  | x | x | x | x |  | x | x | x | x |  |  |  |  |  |  |  |  |  |  |  |  |

1. Akl EA, Oxman AD, Herrin J, Vist GE, Terrenato I, Sperati F, et al. Framing of health information messages. Cochrane Database of Systematic Reviews. 2011(12):CD006777. [↑](#footnote-ref-1)
2. Akl EA, Oxman AD, Herrin J, Vist GE, Terrenato I, Sperati F, et al. Using alternative statistical formats for presenting risks and risk reductions. Cochrane Database of Systematic Reviews. 2011(3):CD006776. [↑](#footnote-ref-2)
