# Supplementary material for: Knowledge translation strategies for dissemination with a focus on healthcare recipients: an overview of systematic reviews
Source: Implement Sci. 2020 Mar 4;15:14. doi: 10.1186/s13012-020-0974-3 (PMC7057470; doi:10.1186/s13012-020-0974-3)
Supplement: Supplementary file 7 — Additional file 7. Strategies categorized as having insufficient evidence. [file 13012_2020_974_MOESM7_ESM.docx]

## Additional file 7. Strategies categorized as having insufficient evidence

These statements correspond to category 3 of the evidence rating scheme of Ryan et al. [1]: “insufficient evidence” (Additional file 2).

1. **Providing information or education**

**Single strategies**

- Package leaflets in different formats (qualitative vs quantitative) for improving understanding of adverse effects of medicines [2].
- Patient Information Leaflets use before consultation, screening or surgery or medication information – effect on patient (less) anxiety [3].
- Patient Information Leaflets that are well written and used at an appropriate time – effect on improving knowledge and satisfaction [3].
- Provision of education or communication as a single component – effect on reducing adverse effects from drugs [1].
- Health literacy strategies using alternative numerical presentations, alternative pictorial representations, or alternative media delivered as a single strategy or when compared with other strategies (e.g., video, computer, or slide show presentations) – effect on comprehension and/or intent to seek health care [4].
- Evidence-based written recommendations (clinical practice guidelines) may increase awareness [5].

**Combined strategies**

- Dissemination and communication strategies using different approaches – effect on understanding and use of information [6].
- Communicating precision using different approaches [6].
- Use of social media for health communication [7].
- Online health information delivered using an "adult education style" discussion, instruction and practice in small groups – effect on health literacy [8].
- One to one risk communication (not necessarily face to face) – effect on treatment choices [9].
- Quality of care information (real or hypothetical performance) – effect on choice of higher quality-rated health plan (Faber 2009).
- Public release of performance data regarding any aspect of healthcare organizations or healthcare individuals – effect on change in service selection [10].
- Better dissemination strategies (active or passive) for guidelines or recommendations [11].

1. **Communication and decision-making facilitation**

**Single strategies**

- Use of tailored SMS for dialogue initiation may increase interaction (communication) between researchers and patients [12].

**Combined strategies**

- Consumer health informatics applications – effect on relationship-centered outcomes [13].
- Use of social media – effect on improving the professional and patient relationship and patient empowerment [14].
- Interventions focused on promoting communication about medicines between patients and professionals [1].
- Mobile phone messages between care provider and participant to deliver preventive health care – effect on satisfaction or anxiety [15].
- Delayed prescribing – effect on antimicrobial resistance [1].

1. **Acquiring skills and competencies**

**Single strategies**

*None identified*

**Combined strategies**

- Toolkits (self-test, information sheets, book, CDs, Audio CDs) may improve health status, behavior, and self-efficacy (patients with arthritis) [16].
- Other types of health literacy interventions – effect on health outcomes (knowledge, self-efficacy, behavioral intent, medication adherence, disease prevalence and severity, quality of life and costs) [4].
- Self-management and self-monitoring of antithrombotic medicine – effect on major hemorrhages and thromboembolic events or mortality, which may be because these events are rare thus studies are likely to have insufficient power to detect a clinical difference [1].
- Provision of training by pharmacists to improve medication adherence [1].
- Medicine self-administration programs – effect on medicines adherence, knowledge, errors or satisfaction [1].
- Life coaching interventions to improve self-efficacy and self-empowerment – effect on health-related outcomes. Note: the life coaching could be in the form of: individual telephone coaching, individual face-to- face-to-face, telephone, or internet coaching or a combination of these methods. The studies including disadvantaged patients showed the most convincing results [17].

1. **Behavior change support**

**Single strategies**

- Email vs standard mail or usual care may change behaviour or understanding for preventive health actions [18].

**Combined strategies**

- Electronic resources such as the internet and telecommunications systems – effect on any of the measured outcomes. However, it may improve the nurse-patient relationship [19].
- Alternative statistical formats – impact on health behaviour [20].
- Adding personal stories to patient decision aids – impact on support for people’s informed decision-making [21].
- When email counselling was compared to telephone counselling for the majority of measures on patients there was no difference between groups [22]. Where there were differences these showed that telephone counselling leads to greater change in lifestyle modification factors than email counselling.

1. **(Personal) support**

**Single strategies**

*None identified*

**Combined strategies**

- Structured counselling or compliance therapy, or of group or home-based visits – to promote vaccination [1].

1. **Consumer system participationn**

**Single strategies**

*None identified*

**Combined strategies**

- Use of a “patient advisory council” for patient engagement in health care delivery – impact on clinical results, priority setting, patient safety and/or patient satisfaction [23].
- Nursing care through telemedicine – impact on access to healthcare, satisfaction and use of resources [19].
- Use of patient portals allowing patients to access their personal health information – effect on health or proxies for health (mortality, emergency room visits, hospitalizations, heart failure practice visits or risk factors) or empowerment [24].
- Electronic tools for health information exchange (e.g. electronic medical records) – impact on hospital readmission and length of stay [25].
- Information Technology applications implemented to support patient-centered care – impact on intermediate health outcomes (patient or provider satisfaction, health knowledge, behavior and cost) [26].

**References**

1. Ryan R, Santesso N, Lowe D, Hill S, Grimshaw J, Prictor M, et al. Interventions to improve safe and effective medicines use by consumers: An overview of systematic reviews. Cochrane Database Syst Rev 2014;4: CD007768.

2. Pires C, Vigário M, Cavaco A. Readability of medicinal package leaflets: a systematic review. Rev Saude Publica 2015;49: 1-13.

3. Sustersic M, Gauchet A, Foote A, Bosson JL. How best to use and evaluate Patient Information Leaflets given during a consultation: a systematic review of literature reviews. Health Expect 2017;20: 531-42.

4. Berkman ND, Sheridan SL, Donahue KE, Halpern DJ, Viera A, Crotty K, et al. Health literacy interventions and outcomes: an updated systematic review. Evid Rep Technol Assess (Full Rep) 2011: 1-941.

5. Loudon K, Santesso N, Callaghan M, Thornton J, Harbour J, Graham K, et al. Patient and public attitudes to and awareness of clinical practice guidelines: a systematic review with thematic and narrative syntheses. BMC Health Serv Res 2014;14: 321.

6. McCormack L, Sheridan S, Lewis M, Boudewyns V, Melvin CL, Kistler C, et al. Communication and dissemination strategies to facilitate the use of health-related evidence. Evidence report/technology assessment 2013: 1-520.

7. Moorhead SA, Hazlett DE, Harrison L, Carroll JK, Irwin A, Hoving C. A new dimension of health care: Systematic review of the uses, benefits, and limitations of social media for health communication. J Med Internet Res 2013;15: e85.

8. Car J, Lang B, Colledge A, Ung C, Majeed A. Interventions for enhancing consumers' online health literacy. Cochrane Database Syst Rev 2011: CD007092.

9. Edwards A, Hood K, Matthews E, Russell D, Russell I, Barker J, et al. The effectiveness of one-to-one risk communication interventions in health care: a systematic review. Med Decis Making 2000;20: 290-7.

10. Ketelaar NA, Faber MJ, Flottorp S, Rygh LH, Deane KH, Eccles MP. Public release of performance data in changing the behaviour of healthcare consumers, professionals or organisations. Cochrane Database Syst Rev 2011: CD004538.

11. Schipper K, Bakker M, De Wit M, Ket JC, Abma TA. Strategies for disseminating recommendations or guidelines to patients: a systematic review. Implement Sci 2016;11: 82.

12. Fjeldsoe BS, Marshall AL, Miller YD. Behavior change interventions delivered by mobile telephone short-message service. Am J Prev Med 2009;36: 165-73.

13. Gibbons MC, Wilson RF, Samal L, Lehman CU, Dickersin K, Lehmann HP, et al. Impact of consumer health informatics applications. Evidence Report/Technology Assessment 2009: 1-546.

14. Smailhodzic E, Hooijsma W, Boonstra A, Langley DJ. Social media use in healthcare: A systematic review of effects on patients and on their relationship with healthcare professionals. BMC Health Serv Res 2016;16: 442.

15. Vodopivec-Jamsek V, de Jongh T, Gurol-Urganci I, Atun R, Car J. Mobile phone messaging for preventive health care. Cochrane Database Syst Rev 2012;12: CD007457.

16. Yamada J, Shorkey A, Barwick M, Widger K, Stevens BJ. The effectiveness of toolkits as knowledge translation strategies for integrating evidence into clinical care: a systematic review. BMJ Open 2015;5.

17. Ammentorp J, Uhrenfeldt L, Angel F, Ehrensvard M, Carlsen EB, Kofoed PE. Can life coaching improve health outcomes?--A systematic review of intervention studies. BMC Health Serv Res 2013;13: 428.

18. Sawmynaden P, Atherton H, Majeed A, Car J. Email for the provision of information on disease prevention and health promotion. Cochrane Database Syst Rev 2012;11: CD007982.

19. Akesson KM, Saveman BI, Nilsson G. Health care consumers' experiences of information communication technology--a summary of literature. Int J Med Inform 2007;76: 633-45.

20. Akl EA, Oxman AD, Herrin J, Vist GE, Terrenato I, Sperati F, et al. Using alternative statistical formats for presenting risks and risk reductions. Cochrane Database Syst Rev 2011: 1-90.

21. Bekker HL, Winterbottom AE, Butow P, Dillard AJ, Feldman-Stewart D, Fowler FJ, et al. Do personal stories make patient decision aids more effective? A critical review of theory and evidence. BMC Med Inform Decis Mak 2013;13 Suppl 2: S9.

22. Atherton H, Sawmynaden P, Sheikh A, Majeed A, Car J. Email for clinical communication between patients/caregivers and healthcare professionals. Cochrane Database Syst Rev 2012;11: CD007978.

23. Sharma AE, Knox M, Mleczko VL, Olayiwola JN. The impact of patient advisors on healthcare outcomes: a systematic review. BMC Health Serv Res 2017;17: 693.

24. Ammenwerth E, Schnell-Inderst P, Hoerbst A. The impact of electronic patient portals on patient care: A systematic review of controlled trials. J Med Internet Res 2012;14: e162.

25. Health Quality Ontario. Electronic tools for health information exchange: an evidence-based analysis. Ontario Health Technology Assessment Series [Internet]. 2013; 13(11):[1–76 pp.]. Available from: <http://www.hqontario.ca/en/documents/eds/2013/full-report-OCDM-etools.pdf>.

26. Finkelstein J, Knight A, Marinopoulos S, Gibbons MC, Berger Z, Aboumatar H, et al. Enabling patient-centered care through health information technology. Evid Rep Technol Assess (Full Rep) 2012: 1-1531.
