# Supplementary material for: Knowledge translation strategies for dissemination with a focus on healthcare recipients: an overview of systematic reviews
Source: Implement Sci. 2020 Mar 4;15:14. doi: 10.1186/s13012-020-0974-3 (PMC7057470; doi:10.1186/s13012-020-0974-3)
Supplement: Supplementary file 8 — Additional file 8. Research gaps. [file 13012_2020_974_MOESM8_ESM.docx]

## Additional file 8: Research gaps

The research gaps correspond to category 4 of the evidence rating scheme of Ryan et al. (1): “insufficient evidence to determine” (Additional file 2).

Health risk-communication

Rigorous studies are needed to evaluate the different modes of risk communication in different clinical settings or conditions (2). Regarding use of alternative statistical presentations future research should be conducted in real life settings and address some populations of interest (e.g. individuals with low numeracy, non-English speaking countries), unstudied presentations (e.g. odds ratio, number needed to treat for an additional harmful outcome), and more relevant outcomes (e.g. actual behaviour and consistency of decisions with values) (3). To evaluate the effects of attribute framing (positive versus negative) and of goal framing (gain versus loss) of the same health information future research should use high quality randomised controlled trial designs, be conducted in real life settings, and assess outcomes such as actual behavior and the consistency of choices and behaviours with preferences and values. Future systematic reviews should aim to explain the unexplained heterogeneity between studies; that is, through meta-regression analyses exploring potential effect modifiers such as different perceived consequences (avoiding a bad health state as opposed to attaining a good health state), type of heath message, level of baseline risk, level of involvement, and perceived susceptibility (4). When comparing the effects of words versus numbers in communicating the probability of adverse effects or harms in written health information, more studies should focus on the impact of personal and contextual factors, including the setting, disease, and the numeracy and educational level of the participants. Research should be conducted in real life settings and measure more relevant outcomes, such as actual behavior (including decisions and medication adherence for example) and whether decisions are in line with personal values. A further unanswered question is how different formats for describing the frequency of adverse effects are interpreted when they are presented together with treatment benefits, since these are also often overestimated by patients. Qualitative research methods may be able to shed some light into how people come to assign probabilities to words ((5).

Decision Aids and Coaching

The coaching methods used should be described more comprehensively, and research in this should be supplemented by a more qualitative approach (6). Given the small number of trials and variability in results, further research is required to determine the effectiveness of decision coaching over and above patient decision aids (PtDAs) for health decisions. Further research is also needed on the effectiveness of PtDAs and/or coaching in disadvantaged populations, e.g. the very sick, children, the very old, people facing treatment or screening decisions, individuals who have the most difficulty accessing health care, or those of lower socioeconomic status, lower numeracy, or lower literacy. Research is required to determine the cost-effectiveness of decision coaching and to evaluate long-term outcomes (7, 8). More research is necessary to determine whether the addition of personal stories to PtDAs is more likely to encourage people to make decisions based on another’s judgments or choice or on more deliberative reasoning than the PtDA alone (9).

Clinical practice guidelines and package leaflets

Guideline producers need to make clear how the information is relevant to the reader and how it can be used to make healthcare improvements. The awareness of guidelines is generally low and guideline producers cannot assume that the public has a more positive perception of their material than of alternative sources of health information (10). More research about the effects, efficiency and barriers of existing dissemination strategies and the role of patient organizations in that process, are also needed (11). Further research is needed to establish the association between the characteristics of self-management support and outcomes and to optimize the design of self-management tools included in or with guidelines for both health care providers and patients (12).

For package information leaflets of medicinal products, more studies on patient comprehension of adverse drug reactions and specific topics, such as precautions, interactions, and contraindications, are necessary. More multicenter studies are also needed to study intra- and inter-cultural differences, such as dialectal differences, larger and more varied samples of package leaflets, enrolling more participants, and development of new metrics and legibility formulas for specific languages (e.g. Portuguese) (13).

Multimedia

More research is needed to examine the degree of influence of fictional medical television on the relationship between users and providers as well as the long-term effect of behavior change. Further, research of its impact in more diverse and older populations, and on other leading causes of morbidity and mortality, such as drug and alcohol use, tobacco use, obesity and heart disease is needed (14). More research is needed to evaluate the best practices regarding particular media for patient education experiences and to assess the reading level of text in print and multimedia tools (15).

Consumer Health Informatics (CHI) applications

More research is necessary to understand the role of CHI applications targeting children, adolescents, the elderly, and specifically nontraditional patient caregivers (family members, friends, allied health workers) (16). This research should be done to understand consumer desires and needs versus provider perceptions of patient desires and needs in terms of emerging CHI applications and tools. It will be important to explain the effect of CHI applications on health outcomes among racial and ethnic minority populations, low-literate populations, and the potential effect of these applications on health care disparities. More research is also needed to understand how social determinants may impact on CHI access, utilization, efficacy, costs, and/or outcomes at the individual level and healthcare disparities at the population level. The role of Web 2.0, social networking, and health gaming technology in CHI should be evaluated. Also, the role of other technological platforms including cell phones, PDA’s, TV, satellite, on Demand, Health Gaming platforms (Wii, XBOX, Gamecube, etc.) needs to be understood, as well as the potential role of CHI applications exploring the dynamics and potential utility of using social networking applications (Skype, Twitter, MySpace, Facebook, You Tube, blogs, Second life, Yoville and Farmville etc.) to support behavior change or improve health outcomes (16). It will be important that different professions in software engineering and health providers work together to create useful applications for health care (17).

Health IT that supports patient-centered care (PCC)

More studies are needed on health IT focused on the needs and preferences of individual patients and improved shared decision making between patients, their families, and providers; patient-clinician communication; and access to medical information (18). The impact of health IT on health care across populations remains unclear. For instance, how patients with cognitive or physical impairments interact with health IT or what is the impact that racial and ethnic backgrounds, education, and socioeconomic levels have on the effectiveness of health IT that address components of patient-centered care (PCC). With more care being delivered in the home and community-based setting, more research is also necessary to elucidate the impact of community, environment, and culture on the health care utilization and health outcomes associated with health IT. Other important areas of study are the development of integrative measures for gauging compliance of health IT applications with PCC principles, integrating PCC components into electronic health record systems used in routine clinical practice, how different types of outcomes interact when health IT supports PCC, and how to use principles of PCC in a systematic way. More studies addressing cost or sustainability are also necessary. For caregivers more research regarding the effectiveness of health IT interventions for improving the quality of care, coordination of care, and costs, are needed (18). Future research should also exploit the full potential of IT platforms in health care, selecting appropriate ways to measure health behavior change and develop a common framework to analyze and understand their different components. Assessment of the safety, effectiveness, efficiency, usability, and user satisfaction with these platforms are needed (19).

Social networking sites to change health behaviors

Future research should focus on identifying the features that increase the engagement and retention of the target audience, as well as the specific characteristics that promote long-term behavior change and improve cost-effectiveness (20). Single-component interventions, factorial design methods, and adaptive designs should be considered more often, so that the effectiveness of each of the social networking sites components can be clearly evaluated. More experimental studies are needed and the type of comparison group should be considered carefully. Study duration should also be thoughtfully planned so that engagement and retention are optimized and enough time is allowed for the specific type of behavior change to occur. An interesting hypothesis that remains untested, is that social networking sites may be used in a synergistic way with personal health records and mobile devices, allowing consumers to continuously benefit from the daily knowledge, accountability, support and influence that their social connections (20). More research will be important to examine interventions delivered via existing popular social network websites, such as Facebook, given their proven ability to attract and retain participants and potential for mass dissemination. Such interventions should be responsive to the way people use online social networks (predominantly with existing friends and for entertainment). In addition to high-quality efficacy studies, pragmatic randomized trials are also required to determine the interventions’ ability to mass disseminate in a real-world setting (21). It will also be important to research the effects of patients’ use of different types of social media on the relationship and communication between patients and healthcare professionals, both in the short and long term (22, 23).

Electronic patient portals

The available evidence does not support the assumption that patient portals are a way to empower patients and improve patient care. Further studies of larger sample size and with harmonized outcome indicators are needed (24).

Email

Qualitative and quantitative research methods could be utilized to explore in more detail the factors that are important for providers and patients when using email to communicate with each other, and for disease prevention and health promotion. Changes to technology are often rapid and care is needed to choose outcomes that remain applicable in the face of such changes. This may also involve concentrating on those elements that make email different from other methods of communication (e.g. lack of vocal cues, asynchronous nature, stability of email address versus other personal details). Such factors do not change with time as the technology changes. Further, policymakers may wish to know whether email is more expensive or cost-effective than other methods, as well as how effective it is. The use of theoretical frameworks in evaluating complex interventions would be a valuable addition to any future research (25, 26) .

Mobile phones

Mobile phone apps are seen as a potential low-cost way to deliver health interventions to both the general and at-risk populations. Many mobile phone apps exist, however, rigorous research to test their effectiveness and acceptability is lacking. They are currently being used without a thorough understanding of their associated risks and benefits (27). With respect to text messaging for behavior change, future research should take into consideration the way that the message is framed to ensure that it is written in the most appropriate way for the population. Future studies should also report on process measures associated with intervention delivery, such as the number of SMS messages sent, the number of SMS replies, how participants received the SMS messages, and how stored SMS messages are treated. Studies should also explicitly describe the theoretical constructs being targeted in interventions. Researchers should address ethical concerns that may arise from delivering health care via a mobile phone. If text message intervention studies are built on evidence and theory, the potential impact of these studies is likely to be much greater. Cost-benefit analyses should be considered as well (28). Future research should also include different age groups that may benefit from preventive health interventions and should have a longer duration. Future studies should also compare effects in different contexts, for example in low and middle income countries with weaker health systems and significant shortages in the health workforce (29).

Health literacy

Regarding enhancing consumers’ online health literacy (skills to search, evaluate and use online health information) more research of quality, including different settings is necessary to analyze the extent to which online health literacy reduces a barrier to using the internet for health information (30). Future research priorities should include justifying appropriate cutoffs for health literacy levels prior to conducting studies; developing tools that measure additional related skills, particularly oral (spoken) health literacy; and examining mediators and moderators of the effect of health literacy. Priorities in advancing the design features of interventions include testing novel approaches to increase motivation, techniques for delivering information orally or numerically, “work around” interventions such as patient advocates; determining the effective components of already-tested interventions; and determining the cost-effectiveness. Future research could enhance the confidence in the more universal applicability of results by including more broadly based and representative samples (31).

Patient advisory councils

Research on the impact of interventions involving patient advisory councils on clinical care outcomes, patient safety, and patient satisfaction are needed to support patient-centered care (32).

Communication and dissemination strategies

Future research on communication and dissemination strategies to facilitate the use of health-related evidence should rely more on accepted theoretical constructs and models. When designing interventions researchers should conduct some prior-needs assessments with target audiences, focusing on the audience subgroups with the greatest needs. For future research, research teams should focus on: designing robust trials or observational studies; describing and defending choices of intermediate and ultimate outcomes; applying modeling or other advanced statistical and analytic techniques to account for confounders, interactions, and similar complications in data, and addressing temporal aspects of outcomes; and thoroughly describing all aspects of study design and conduct, especially for interventions (33). Further research should also evaluate Patient-mediated Knowledge Translation (PKT) interventions in more patients, or patients with different conditions; different types of PKT interventions for patients and for providers; and potential harms associated with interventions (34).

**References**

1. Ryan R, Santesso N, Lowe D, Hill S, Grimshaw J, Prictor M, et al. Interventions to improve safe and effective medicines use by consumers: An overview of systematic reviews. Cochrane Database of Systematic Reviews. 2014;4:CD007768.

2. Edwards A, Hood K, Matthews E, Russell D, Russell I, Barker J, et al. The effectiveness of one-to-one risk communication interventions in health care: a systematic review. Med Decis Making. 2000;20(3):290-7.

3. Akl EA, Oxman AD, Herrin J, Vist GE, Terrenato I, Sperati F, et al. Using alternative statistical formats for presenting risks and risk reductions. Cochrane Database of Systematic Reviews. 2011(3):1-90.

4. Akl EA, Oxman AD, Herrin J, Vist GE, Terrenato I, Sperati F, et al. Framing of health information messages. Cochrane Database of Systematic Reviews. 2011(12):CD006777.

5. Buchter R, Fechtelpeter D, Knelangen M, Ehrlich M, Waltering A. Words or numbers? Communicating risk of adverse effects in written consumer health information: a systematic review and meta-analysis. BMC Med Inform Decis Mak. 2014;14:76.

6. Ammentorp J, Uhrenfeldt L, Angel F, Ehrensvard M, Carlsen EB, Kofoed PE. Can life coaching improve health outcomes?--A systematic review of intervention studies. BMC Health Serv Res. 2013;13:428.

7. Stacey D, Kryworuchko J, Bennett C, Murray MA, Mullan S, Legare F. Decision coaching to prepare patients for making health decisions: a systematic review of decision coaching in trials of patient decision AIDS. Med Decis Making. 2012;32(3):E22-33.

8. Stacey D, Legare F, Lewis K, Barry MJ, Bennett CL, Eden KB, et al. Decision aids for people facing health treatment or screening decisions. Cochrane Database Syst Rev. 2017;4:CD001431.

9. Bekker HL, Winterbottom AE, Butow P, Dillard AJ, Feldman-Stewart D, Fowler FJ, et al. Do personal stories make patient decision aids more effective? A critical review of theory and evidence. BMC Med Inform Decis Mak. 2013;13 Suppl 2:S9.

10. Loudon K, Santesso N, Callaghan M, Thornton J, Harbour J, Graham K, et al. Patient and public attitudes to and awareness of clinical practice guidelines: a systematic review with thematic and narrative syntheses. BMC Health Serv Res. 2014;14:321.

11. Schipper K, Bakker M, De Wit M, Ket JC, Abma TA. Strategies for disseminating recommendations or guidelines to patients: a systematic review. Implement Sci. 2016;11(1):82.

12. Vernooij RW, Willson M, Gagliardi AR, members of the Guidelines International Network Implementation Working G. Characterizing patient-oriented tools that could be packaged with guidelines to promote self-management and guideline adoption: a meta-review. Implement Sci. 2016;11:52.

13. Pires C, Vigário M, Cavaco A. Readability of medicinal package leaflets: a systematic review. Rev Saude Publica. 2015;49:1-13.

14. Hoffman BL, Shensa A, Wessel C, Hoffman R, Primack BA. Exposure to fictional medical television and health: a systematic review. Health Educ Res. 2017;32(2):107-23.

15. Wilson EA, Makoul G, Bojarski EA, Bailey SC, Waite KR, Rapp DN, et al. Comparative analysis of print and multimedia health materials: a review of the literature. Patient Educ Couns. 2012;89(1):7-14.

16. Gibbons MC, Wilson RF, Samal L, Lehman CU, Dickersin K, Lehmann HP, et al. Impact of consumer health informatics applications. Evid Rep Technol Assess (Full Rep). 2009(188):1-546.

17. Akesson KM, Saveman BI, Nilsson G. Health care consumers' experiences of information communication technology--a summary of literature. Int J Med Inform. 2007;76(9):633-45.

18. Finkelstein J, Knight A, Marinopoulos S, Gibbons MC, Berger Z, Aboumatar H, et al. Enabling patient-centered care through health information technology. Evid Rep Technol Assess (Full Rep). 2012(206):1-1531.

19. Sawesi S, Rashrash M, Phalakornkule K, Carpenter JS, Jones JF. The Impact of Information Technology on Patient Engagement and Health Behavior Change: A Systematic Review of the Literature. JMIR Med Inform. 2016;4(1):e1.

20. Laranjo L, Arguel A, Neves AL, Gallagher AM, Kaplan R, Mortimer N, et al. The influence of social networking sites on health behavior change: a systematic review and meta-analysis. J Am Med Inform Assoc. 2015;22(1):243-56.

21. Maher CA, Lewis LK, Ferrar K, Marshall S, De Bourdeaudhuij I, Vandelanotte C. Are health behavior change interventions that use online social networks effective? A systematic review. J Med Internet Res. 2014;16(2):e40.

22. Moorhead SA, Hazlett DE, Harrison L, Carroll JK, Irwin A, Hoving C. A new dimension of health care: Systematic review of the uses, benefits, and limitations of social media for health communication. J Med Internet Res. 2013;15(4):e85.

23. Smailhodzic E, Hooijsma W, Boonstra A, Langley DJ. Social media use in healthcare: A systematic review of effects on patients and on their relationship with healthcare professionals. BMC Health Serv Res. 2016;16:442.

24. Ammenwerth E, Schnell-Inderst P, Hoerbst A. The impact of electronic patient portals on patient care: A systematic review of controlled trials. J Med Internet Res. 2012;14(6):e162.

25. Atherton H, Sawmynaden P, Sheikh A, Majeed A, Car J. Email for clinical communication between patients/caregivers and healthcare professionals. Cochrane Database Syst Rev. 2012;11:CD007978.

26. Sawmynaden P, Atherton H, Majeed A, Car J. Email for the provision of information on disease prevention and health promotion. Cochrane Database Syst Rev. 2012;11:CD007982.

27. Zhao J, Freeman B, Li M. Can Mobile Phone Apps Influence People's Health Behavior Change? An Evidence Review. J Med Internet Res. 2016;18(11):e287.

28. Cole-Lewis H, Kershaw T. Text messaging as a tool for behavior change in disease prevention and management. Epidemiol Rev. 2010;32:56-69.

29. Vodopivec-Jamsek V, de Jongh T, Gurol-Urganci I, Atun R, Car J. Mobile phone messaging for preventive health care. Cochrane Database Syst Rev. 2012;12:CD007457.

30. Car J, Lang B, Colledge A, Ung C, Majeed A. Interventions for enhancing consumers' online health literacy. Cochrane Database Syst Rev. 2011(6):CD007092.

31. Berkman ND, Sheridan SL, Donahue KE, Halpern DJ, Viera A, Crotty K, et al. Health literacy interventions and outcomes: an updated systematic review. Evid Rep Technol Assess (Full Rep). 2011(199):1-941.

32. Sharma AE, Knox M, Mleczko VL, Olayiwola JN. The impact of patient advisors on healthcare outcomes: a systematic review. BMC Health Serv Res. 2017;17(1):693.

33. McCormack L, Sheridan S, Lewis M, Boudewyns V, Melvin CL, Kistler C, et al. Communication and dissemination strategies to facilitate the use of health-related evidence. Evid Rep Technol Assess (Full Rep). 2013(213):1-520.

34. Gagliardi AR, Legare F, Brouwers MC, Webster F, Badley E, Straus S. Patient-mediated knowledge translation (PKT) interventions for clinical encounters: A systematic review. Implementation Science. 2016;11(1):26.
